# A two-strata energy flux system driven by a stress hormone prioritizes cardiac energetics

**DOI:** 10.1038/s41392-025-02402-9

**Published:** 2025-09-26

**Authors:** Zhiheng Rao, Zhichao Chen, Yuxuan Bao, Zhenzhen Lu, Yuli Tang, Jiamei Zhu, Jianjia Ma, Siyang Dong, Jiawei Shi, Suhui Sheng, Yajing Chen, Jiaojiao Wang, Alan Vengai Mukondiwa, Ziyue Li, Xulan Wang, Zibo Huang, Chi Li, Wumengwei Ding, Mengjie Chen, Ziyi Han, Cong Wang, Xuebo Pan, Xiaojie Wang, Hong Zhu, Li Lin, Zhifeng Huang, Weiqin Lu, Xiaokun Li, Yongde Luo

**Affiliations:** 1 School of Pharmaceutical Sciences, Wenzhou Medical University, Wenzhou, Zhejiang China; 2 The Second Affiliated Hospital and Yuying Children’s Hospital, Wenzhou Medical University, Wenzhou, Zhejiang China; 3 Oujiang Laboratory (Zhejiang Lab for Regenerative Medicine, Vision and Brain Health), Wenzhou, Zhejiang China; 4 Department of Endocrinology, The First Affiliated Hospital of Wenzhou Medical University, Wenzhou, Zhejiang China; 5 Department of Pharmaceutical Sciences, School of Pharmacy, The University of Texas at El Paso, El Paso, TX USA

**Keywords:** Physiology, Endocrine system and metabolic diseases, Cardiology, Molecular biology, Biologics

## Abstract

The heart, an organ with a continuously high demand for energy, inherently lacks substantial reserves. The precise mechanisms that prioritize energy allocation to cardiac mitochondria, ensuring steady-state ATP production amidst high-energy organs, remain poorly understood. Our study sheds light on this process by identifying a two-strata flux system driven by the starvation hormone FGF21. We demonstrate that systemic disruptions in interorgan metabolite mobilization and transcardiac flux, arising from either adipose lipolysis or hepatic ketogenesis due to FGF21 deficiency, directly impair cardiac energetic performance. Locally, this impairment is linked to compromised intracardiac utilization of various metabolites via ketolysis and oxidation pathways, along with hindered mitochondrial biogenesis, TCA cycle, ETC flow, and OXPHOS. Consequently, the heart shifts to a hypometabolic, glycolytic, and hypoenergy state, with a reduced capacity to cope with physiological stressors such as fasting, starvation, strenuous exercise, endurance training, and cold exposure, leading to a diminished heart rate, contractility, and hemodynamic stability. Pharmacological or genetic restoration of FGF21 ameliorates these defects, reenergizing stress-exhausted hearts. This hierarchical energy-prioritizing mechanism is orchestrated by the LKB1-AMPK-mTOR energy stress response pathways. Disrupting cardiac LKB1 or mTOR pathways, akin to stalling mitochondrial energy conduits, obstructs the FGF21-governed cardiac energetic potential. Our findings reveal an essential two-strata energy flux system critical for cardiac energetic efficiency regulated by FGF21, which spatiotemporally optimizes interorgan and transcardiac metabolite flux and intracardiac mitochondrial energy sufficiency. This discovery informs the design of strategies for treating cardiac diseases linked to mitochondrial or energy deficiencies.

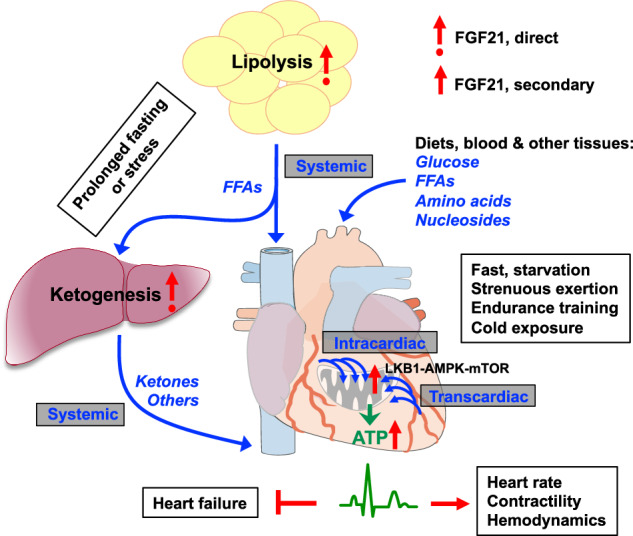

## Introduction

The heart, a relentless engine, demands an exceptionally high and incessant supply of ATP to maintain functional efficiency and hemodynamic stability,^1,2^ outstripping any other high-energy organs that get to rest during sleep or inactivity. Notably, it does not store sufficient metabolic substrates but relies on a constant supply from the bloodstream, primarily sourced from the liver, adipose depot, and dietary intake. The timely availability of sufficient free fatty acids (FFAs), amino acids, ketone bodies (beta-hydroxybutyrate and acetoacetate), and sugars to the heart is critical for managing or surviving energy crises, including physiological fluctuations, nutrient shifts, fast/starvation, strenuous workload, aerobic exercise, endurance training, cold, and pathological challenges. Mitochondria enriched in cardiomyocytes are essential for processing these metabolites into a steady-state ATP pool through catabolic pathways, oxidation, glycolysis, ketosis, and ultimately, the TCA cycle and oxidative phosphorylation (OXPHOS). They also ensure metabolic flexibility and manage calcium dynamics in the heart. Mitochondrial dysfunction or insufficient energy generation underpins cardiac energy insufficiency and functional inefficiency, hallmark features of major cardiovascular diseases (such as cardiomyopathy and heart failure), metabolic disorders, and issues in fitness and longevity. Replenishing metabolites, especially ketones and short-chain FFAs, can partially energize diseased hearts.^3,4^ However, the mechanisms that prioritize cardiac energy allocation among vital organs, ensuring immense ATP production, remain elusive, hindering innovative therapeutic approaches.

Recent studies have highlighted the salutary effects of FGF21 as a stress-responsive hormone on systemic metabolic homeostasis and, particularly, as a sensitive signal for mitochondrial defects manifesting in muscular and neurological diseases.^5–13^ These findings position FGF21 as a plausible candidate regulator for optimizing energy allocation and ensuring cardiac energy priority. Metabolically, FGF21 is known to promote ketogenesis and lipolysis during fasting or energy stress. However, the liver and white adipose tissue (WAT) do not utilize these products themselves. Except for brown adipose tissue (BAT), which benefits from the action of FGF21 but only for cold adaptation, the chief beneficiary organ(s) of these FGF21-driven metabolic processes remain unidentified. Furthermore, elevated serum FGF21 levels are associated with increased cardiovascular risk and mortality, including cardiomyopathy and heart failure, particularly in patients with reduced ejection fraction and systolic or diastolic dysfunctions.^14–17^ Genetic Mendelian meta-analyses have identified major alleles of rs838133, rs739320 and rs499765 in the regulatory and exon 1 regions of the *FGF21* gene that are associated with cardiometabolic outcomes, serum lipid levels, and dietary preferences.^18–20^ FGF21 analogs or mimetics improve cardiometabolic parameters in conditions such as steatohepatitis, fibrosis, obesity, hyperlipidemia, or type 2 diabetes, which are risk factors for cardiovascular events.^21–23^ In mouse models, diseased hearts (or cardiomyocytes) secrete FGF21, and FGF21 improves pathological agent-induced cardiomyopathy, hypertrophy, ischemic damage, and heart failure by reducing lipid accumulation, oxidative stress, inflammation, and apoptosis, while promoting mitochondrial health.^24–27^ Paradoxically, unlike adipose tissues, the pancreas, and the liver, cardiomyocytes express very low, if any, levels of KLB, the obligatory coreceptor for FGF21, leaving the mechanism underlying the cardiac effects of FGF21 unclear.

In this study, we unveil a hitherto unappreciated, unified mechanism through which a hierarchal, two-pronged energy flux system driven by FGF21 governs cardiac energetic efficiency. FGF21 deficiency results in a hypometabolic, glycolytic, and hypoenergetic state, rendering the heart intolerant to stressors such as fasting, starvation, physical exertion, endurance training, and cold challenge, leading to compromised heart rate and contractility. Reinstating FGF21 reestablishes cardiac energetic performance. This is achieved by coordinating intracardiac oxidative catabolism of diverse metabolites and intermediates, ketolysis, and mitochondrial function, along with systemic hepatic ketogenesis and adipose tissue lipolysis, in a spatiotemporal manner. The control of cardiac energetics and functional performance by FGF21 under stress is indirect and mediated by the nutrient-sensing pathways LKB1-AMPK and mTOR, which are likely activated by influxing metabolites such as AICAR, AMP, arginine, and leucine. Disruption of these systemic and local metabolic processes impairs the cardiac energetics maintained by FGF21. Thus, FGF21 emerges as a critical determinant factor for sustaining heart energy sufficiency and functional efficiency by driving a systemic-to-intracardiac energy flux network, with centrality on prioritizing intracardiac mitochondrial energy production. Our findings provide significant new insights into mitochondrial function, metabolic flux, and heart physiology, as well as potential strategic designs for cardiovascular diseases associated with mitochondrial and energy deficiencies.

## Result

### Germline FGF21 deletion triggers systemic metabolic shifts linked to mitochondrial and cardiac energetic defects

**Fig. 1:**
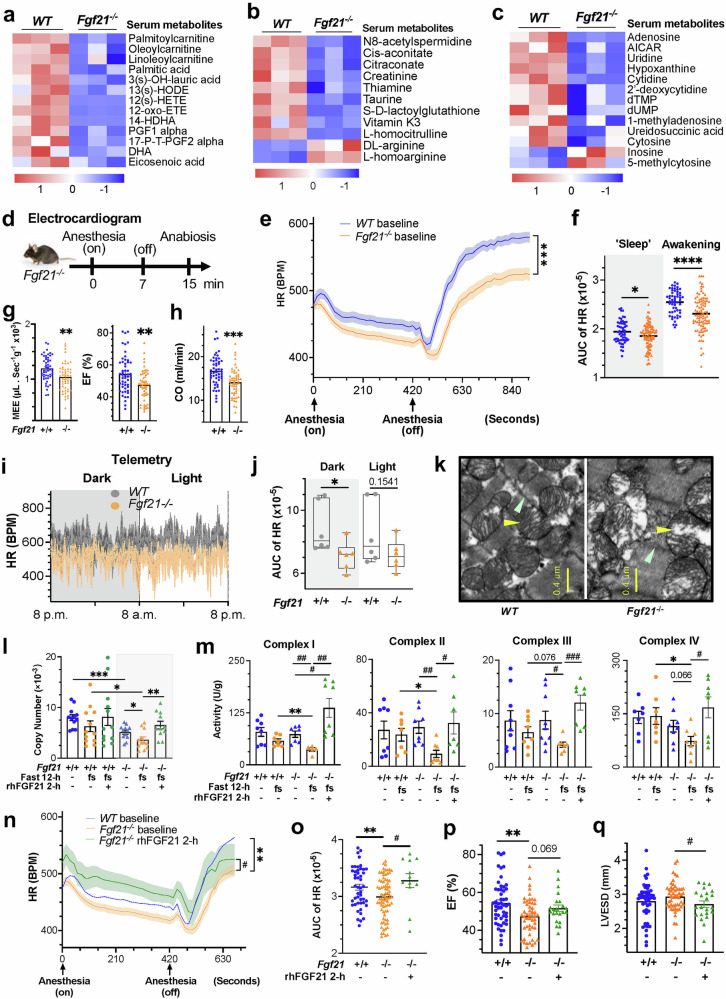
FGF21 deficiency alters systemic metabolites linked to mitochondrial and cardiac energetic defects. **a**–**c** Heatmap of significantly altered serum metabolites, including mitochondrial FAO precursors, fatty acids and oxylipins, TCA cycle intermediates, and nucleosides, in *Fgf21*^*−/−*^ (n = 3) and WT (n = 3) mice. *p* < 0.05; fold change (FC) > 1.5 or < 0.67. See also Fig. S1a–c and S1e. For changes in serum phosphocreatine levels, see Fig. S1d. S, supplementary. **d** Experimental procedure for electrocardiogram analysis under anesthesia and anabiosis simulating ‘sleep’ (anesthesia on) and ‘awakening’ (anesthesia off) stages of torpor. ‘Sleep’, a sedentary state under anesthetic inhalation; Awakening, a recovery and active state following anesthetic removal. **e** Heart rate (HR) changes in *Fgf21*^*−/−*^ (n = 95) vs WT (n = 56) mice measured in d under normal conditions. BPM, beats per minute. **f** Area-under-curve (AUC) analysis of the HR excursion curves in e. See Fig. S2a. **g**, **h** Echocardiogram analysis of the mechano-energetic efficiency (MEE), ejection fraction (EF) and cardiac output (CO) of *Fgf21*^*-/-*^ (n = 51) vs WT (n = 51) mice. See Fig. S2b-S2h for other Echo parameters. **i** Telemetry monitoring of HR changes over 24 hours in representative *Fgf21*^*-/-*^ vs WT mice under normal conditions. **j** AUC analysis of cumulative HR excursion curves of *Fgf21*^*-/-*^ (n = 6) vs WT (n = 6) mice in i. Changes in ambulatory movement and body temperature are shown in Fig. S2j-S2l. **k** Representative TEM images of left ventricle cross-sections from *Fgf21*^*-/-*^ vs WT mice under normal conditions. Yellow arrowhead, mitochondria. Cyan arrowhead, Z line. See Fig. S3e for broader, low-magnification images. The changes in cardiac morphometric parameters, mild dilatation and insignificant fibrosis are shown in Fig. S3. **l** Mitochondrial DNA content of hearts from *Fgf21*^*-/-*^ (n = 11-12) vs WT (n = 12) mice. **m** Catalytic activities of mitochondrial complexes I-IV in the hearts of *Fgf21*^*-/-*^ (n = 7-9) vs WT (n = 7-9) mice. h, hour. **n**, **o** HR Changes (n) with rhFGF21 treatment (n = 12) and AUC analysis (o) of *Fgf21*^*-/-*^ (n = 78) vs WT (n = 47) mice under normal conditions. **p**, **q** Changes in EF and LVSED with rhFGF21 treatment (n = 22) in *Fgf21*^*-/-*^ (n = 51) vs WT (n = 51) mice, as in n. See Fig. S3g-S3i for other parameters. The data are presented as the means ± s.e.m.s. (**a**–**c**, **e**–**j**) Two-tailed unpaired Student’s t-test; (**l**–**q**) ordinary one-way ANOVA followed by Tukey’s test; *p < 0.05, **p < 0.01, ***p < 0.001, ****p < 0.0001. *, *Fgf21*^*-/-*^ vs WT groups by Student’s t test or one-way ANOVA. ^$^, between treatment groups in WT mice. ^#^, between treatment groups in *Fgf21*^*-/-*^ mice. All these also apply to other figures

Although the heart is shielded from hypertrophy and dysfunction by FGF21 treatment under agent-induced pathological conditions,^24^ only minor to mild cardiovascular changes are observed under basal, unstressed conditions, alongside insignificant global morphometric and metabolic abnormalities, in mice with germline deletion of *Fgf21*.^24,28–30^ This leaves the heart’s reliance on FGF21 and its underlying mechanisms unclear. Given that the heart directly draws metabolic substrates from the circulation sourced from peripheral organs, we approached this question by first examining plasma metabolites. Among 71 out of 986 metabolites, L-palmitoylcarnitine, L-oleoylcarnitine and L-linoleoylcarnitine, which are signature precursors of mitochondrial fatty acid oxidation (FAO), decreased significantly in the FGF21-deficient mice (Fig. 1a, supplementary Fig. 1a–b and supplementary Table 1). The levels of cis-aconitate and citraconate, which are TCA cycle intermediates linked to succinyl-CoA, were decreased (Fig. 1b), suggesting defective mitochondrial FAO and TCA cycle activity. These changes resemble serum anomalies in patients with FFA dehydrogenase or carnitine-acylcarnitine translocase deficiencies, who present with myopathies.^31–33^ Coincidently, several fatty acids (e.g., palmitic acid, 3(s)-OH-lauric acid, docosahexaenoic acid, and eicosatetraenoic acid) and oxylipins were reduced, indicating defective FFA flux. Likewise, the levels of phosphatidylcholines and their precursors (choline and alpha-GPC) (supplementary Fig. 1c), which are essential for mitochondrial and neuromuscular function,^34–36^ were altered. Hydroxylated FFA changes are linked to mitochondrial FAO disorders caused by MTP (HADHA or HADHB) defects, leading to cardiomyopathy with impaired ketogenesis and energy insufficiency.^37^ In support of this finding, N8-acetylspermidine, an MTP activator and cardiomyopathy marker, was reduced (Fig. 1b).^38–41^ Additionally, oxylipins such as HETEs and alpha PGF1/2 are associated with tachycardia, muscle contraction, and vascular tone.^42–45^

The serum creatinine and L-homocitrulline levels decreased, whereas the DL-arginine and L-homoarginine levels increased (Fig. 1b and supplementary Fig. 1d). Creatinine and homoarginine are metabolically linked to creatine (a myocytic energy carrier) and polyamines and are cardiovascular disease biomarkers.^46,47^ These findings suggest potential reductions in the phospho-creatine content (supplementary Fig. 1d, lower), which may affect cardiac energetics. Several amino acids and derivatives, including arginine, glutamine, betaine, and L-citrulline, among others, were altered (supplementary Fig. 1e), impacting the mitochondrial fuel supply, complex IV activity, and peroxisome energy metabolism. Coincidentally, thiamine, taurine, and uridine were significantly reduced,^48–52^ of which thiamine is a cofactor for key TCA cycle enzymes, whereas taurine and uridine are required for electron transport chain (ETC) complex I assembly. These reductions are linked to dry/wet beriberi, cardiomyopathy or heart failure.^53–55^ Serum S-D-lactoylglutathione (SDL), a byproduct of acetone or methylglyoxal (a glycolysis product) detoxification, was reduced (Fig. 1b), indicating a potential shift to a glycolytic and/or ketotic state under stress. Serum purine and thymidine nucleosides (9%), particularly adenosine, cytosine, AICAR, uridine, and hypoxanthine, were reduced, while inosine was increased (Fig. 1c). They are important for mitochondrial turnover, energy expenditure and endurance training.^56–59^ Adenosine regulates heart rate, vasodilation, and energy via ATP, whereas cytidine is important for energy transfer via CTP and for phospholipid synthesis via CDP-DAG. AICAR is an activator of AMPK, a key player with LKB1 in energy metabolism.^60,61^ Additionally, uridine, through UTP and TPP (via thiamine), is linked to the TCA cycle and respiration.^62^ Taken together, these findings indicate that FGF21 loss causes profound changes in systemic metabolites, many of which are essential catabolic substrates, intermediates and regulators of mitochondrial energy metabolism, potentially impacting energy-dependent tissues such as the heart.

To determine whether disturbances in circulating metabolites truly reflect cardiac function changes, we examined heart energetic performance under faulty FGF21 signaling. We synchronized the mice to a torpor-like state, reducing their metabolic rate and physical activity with short-term anesthesia, followed by recovery to an active state upon anesthetic removal. An electrocardiogram (ECG) revealed a significant reduction in the basal heart rate (HR) during both the “sleep” and especially the energy-demanding awakening phases in *Fgf21*-ablated mice (Fig. 1d–f, supplementary Fig. 2a), indicating a reduction in the overall performance of the heart. Echocardiography (Echo) revealed significant decreases in myocardial mechanoenergetic efficiency (MEE), ejection fraction (EF), fractional shortening (FS), and cardiac output (CO) without significant changes in left ventricular end-systolic (LVESD) or end-diastolic (LVEDD) diameters at comparable HRs, indicating reduced contractility and blood-pumping efficiency (Fig. 1g, h, supplementary Fig. 2b–e). There was also a significant decrease in E/A without significant changes in E/E’ or E’/A’ (supplementary Fig. 2f–h), indicating a tendency toward impaired left ventricular diastolic capacity. In vivo telemetry showed a similar reduction in basal HR in conscious, unstressed *Fgf21*-deleted mice fed *ad libitum* (Fig. 1i, j, supplementary Fig. 2i), alongside trends toward statistically insignificant decreases in autonomous pedestrian locomotion and body temperature during the dark phase (supplementary Fig. 2j–l). These findings suggest that, under nonstress, physiological baseline conditions, FGF21 loss leads to mild but unison declines in HR maintenance and critical systolic and diastolic parameters, particularly during the active (dark) phase, without significantly affecting physical activity, body temperature, or behavior, especially during the inactive (light) phase. Thus, the heart is the primary organ that is functionally impacted by whole-body FGF21 deficiency.

Morphometric and anatomical assessments revealed that FGF21 deficiency led to increased heart weight and a trend toward increased size (supplementary Fig. 3a–b), which is consistent with an early report.^24^ Light microscopy revealed increased myocyte cross-sectional areas without significant fibrosis (supplementary Fig. 3c–d). Ultrastructural examination revealed typical intermyofibrillar networks intercalated with mitochondria in both mouse lines (Fig. 1k, supplementary Fig. 3e–f). However, in FGF21-deprived mice, cardiac mitochondria were enlarged by approximately 25%, more disarrayed, and frequently vacuolated, with broader but mildly fuzzy or diffuse, cardiomyocytic Z-lines. The mitochondrial DNA (mtDNA) content was significantly reduced (Fig. 1l), which was consistent with reduced serum nucleosides (Fig. 1c). Analysis of ETC complexes I-IV revealed insignificant changes in catalytical activity under normal, energy-sufficient, and resting conditions in *Fgf21*^*-/-*^ mice (Fig. 1m). However, following a 12-h fast, which limits the external energy supply, these activities significantly decreased, unlike those in wild-type (WT) mice. Considering the amplification effect of the enzymatic cascade, these stepwise reductions suggest substantial ETC impairments, which lead to ATP insufficiency due to FGF21 abolition (see later sectional Fig. 7g and supplementary Fig. 12a), undermining the energy stress response. This finding is concordant with the reduced levels of circulating metabolites as sources of myocardial energy. Contrarily, the administration of rhFGF21 (recombinant human FGF21) for 2 hours (h) acutely improved Complex I-IV activity, suggesting a correctable metabolic vulnerability rather than an irreversible myocyte development defect. Correspondingly, rhFGF21 restored HR, EF, and FS and improved LVESD and LVEDD without significantly affecting CO under normal conditions (Fig. 1n–q, supplementary Fig. 3g–i). During the later awakening stage, the HR of *Fgf21*-null mice remained lower than that of WT mice, suggesting the need for extended treatment. These findings suggest that cardiac dysfunction in FGF21-deficient mice, resembling mild mitochondrial cardiomyopathy, is attributable to impaired mitochondrial structure and function, leading to reduced energy flow rather than defects in cardiac tissue development. This finding aligns with the roles of FGF21 as a regulator of energy homeostasis and a biomarker for mitochondria deficiencies in muscle and neural diseases.^11,12^

### Systemic FGF21 deficiency impairs cardiac efficiency under physiologically reversible energy stress

The heart is highly sensitive to energy stress and relies on external energy supplies due to limited internal reserves. Our findings suggest that FGF21 loss disrupts systemic metabolite flow and stalls cardiac mitochondrial ATP production, rendering cardiomyocytes vulnerable to stress. We tested cardiac function performance under diverse mild, reversible energy stressors, namely, fast/starvation, strenuous exercise, training, cold, and mild high-fat diet (HFD) feeding. These stressors are revolutionarily relevant and physiologically adaptable. Unlike calorie restriction, a mild HFD reflects overnutrition, which is beneficial for high-energy processes.

**Fig. 2:**
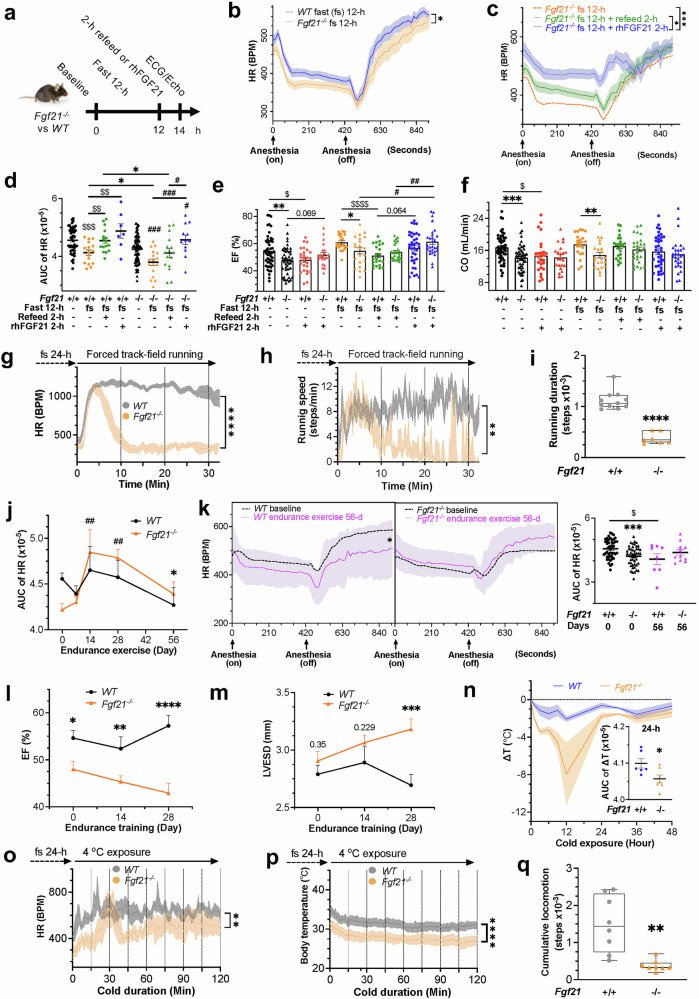
FGF21 deficiency impairs cardiac energetic performance under various physiologically relevant stress conditions. **a** Experimental outline for assessing heart function following 12 hours (h) of fasting. Note that food deprivation for both 12 and 24 hours is referred to as fast (fs) hereafter, except where time constraints are specified (fast, 12-h; fast, 24-h; starvation, 48 h or 2 days). All experiments, except refeeding, were conducted while the mice were still in fasting/starvation after the initial food-deprivation period. **b** Heart rate (HR) changes during fasting in *Fgf21*^*-/-*^ (n = 17) vs WT (n = 17) mice. **c** Comparative HR changes in fasted *Fgf21*^*-/-*^ mice after refeeding and rhFGF21 treatment (1 mg/kg, *i.p*.). For HR changes after a 24-h fast, see Fig. S4g. **d** Area-under-curve (AUC) analysis of HR changes in fasted *Fgf21*^*-/-*^ vs WT mice after refeeding and rhFGF21 treatment for 2 hours. See Fig. S4a for more detailed HR comparisons and S4b for simplified AUC plots. **e**, **f** Changes in ejection fraction (EF) and cardiac output (CO) under the same conditions as in c. *Fgf21*^*-/-*^ refeeding, n = 27; rhFGF21, n = 28; WT, rhFGF21, n = 38. See Fig. S4c–e. **g** HR changes measured by telemetry in response to involuntary running in *Fgf21*^*-/-*^ (n = 7) vs WT (n = 11) mice after 24 hours of fasting. For the experimental scheme see Fig. S5a. **h** Changes in running speed (steps per minute) in the indicated mice as in **g**. **i** Changes in running duration (total steps in 30 minutes) in the indicated mice as in g. For body temperature changes, see Fig. S5b. **j** Impacts of endurance training (2 hours/day for 56 days) on the HR (AUC) in *Fgf21*^*-/-*^ (n = 11) vs WT (n = 8) mice. For HR changes at 7, 14, 28 days, see Fig. S5c–e. **k** HR changes after 56 days of endurance training in the indicated mice. d, day. **l**, **m** Changes in EF and LVESD after 28 days of endurance training in the indicated mice. See Fig. S6a–e. **n** Changes in core body temperature in *Fgf21*^*-/-*^ (n = 7) vs WT (n = 6) mice subjected to a 4 °C challenge for 48 hours. ΔT, core body temperature change between 4 °C and room temperature, as measured by a rectum probe. See Fig. S6f. **o** HR changes measured by telemetry in *Fgf21*^*-/-*^ (n = 8) vs WT (n = 8) mice after 24 hours of fasting and then 2 hours of exposure to 4°C. See Fig. S6g-S6h. **p** Changes in core body temperature in the indicated mice as in o. See Fig. S6i. **q** Changes in cumulative pedestrian activities (total steps in 2 hours) in the indicated mice as in **o**. See Fig. S6j. Data are means ± s.e.m.s; (**b**, **g**–**q**) Two-tailed unpaired Student’s *t*-test; (**c**–**f**) ordinary one-way ANOVA followed by Tukey’s test

The heart adapts to fasting by lowering HR to conserve energy. After 12 hours of fasting, HR decreased while EF and FS increased in both the *Fgf21*^*-/-*^ and WT mice under anesthesia-induced inactivity (Fig. 2a–f, supplementary Fig. 4a–f). *Fgf21*^*-/-*^ mice presented a more pronounced drop in HR and MEE and a greater increase in EF and FS, with a minimal change in CO. These differences persisted at levels with standard caloric intake. LVESD and LVEDD decreased slightly but without statistical significance. Following 2 h of refeeding, the HR escalated in both genotypes but did not fully return to the prefast level in the FGF21-deficient mice (Fig. 2c, d, supplementary Fig. 4a, b), indicating incomplete recovery. Refeeding decreased EF and FS and increased LVESD and LVEDD in WT mice but not in *Fgf21*-null mice (Fig. 2e, supplementary Fig. 4c–e). Acute 2-h rhFGF21 treatment corrected the HR deficits observed under fasting and more effectively than refeeding in the FGF21-depleted mice, which reached the levels observed in the WT littermates under resting conditions. A similar trend of lowered efficiency was again observed during the late stage of the awakening phase following acute rhFGF21 treatment (Fig. 2c, 1n and supplementary Fig. 4a). The additive effect of rhFGF21 was less pronounced in fasted WT mice, which are expected to have increased levels of endogenous FGF21.^6^ Interestingly, rhFGF21 elevated EF and FS while reducing LVESD and LVEDD in *Fgf21*-null but not WT mice, in stark contrast to refeeding. Consequently, the large discrepancy in CO seemed to be offset (Fig. 2f). rhFGF21 was also effective at restoring HR after a 24-h fast (supplementary Fig. 4g). These findings highlight the specific roles of FGF21 in regulating heart rate and contraction. The heart is intolerant to calorie restriction without FGF21, with dysfunctions that refeeding alone cannot remedy autonomously.

Strenuous exercise or workload imposes energy stress on the heart, as much of the systemic energy is directed to skeletal muscle and pulmonary respiration. The mechanism regulating adequate cardiac energy allocation remains unknown. Amidst a 10-min session of intense, telemetry-monitored exercise, the HR surged in both genotypes (data not shown), eliminating the resting HR difference (Fig. 1e, i). Remarkably, after prefasting for 24 hours, the HR dropped sharply within 4 minutes of forced track-field running (Fig. 2g, supplementary Fig. 5a), along with reduced running speed and duration exclusively in the *Fgf21*-null mice (Fig. 2h, i). Body temperature remained unchanged (supplementary Fig. 5b). These findings suggest that without FGF21, the heart is intolerant to high-intensity exercise under energy stress. While the heart is particularly sensitive to FGF21 loss, skeletal muscle is also affected under such conditions.

Long-term endurance training typically results in a reduced HR and increased EF and CO. However, in *Fgf21*-null mice, 2 hours of daily exercise for 56 days led to negligible changes or even elevations in HR, in contrast to the reductions in WT mice (Fig. 2j, k, supplementary Fig. 5c–e). Additionally, EF and FS decreased while LVESD and LVEDD increased, contrary to the improvements noted in WT mice (Fig. 2l, m, supplementary Fig. 6a–b). Improvements in CO and LVAW;s also lagged behind those in WT mice (supplementary Fig. 6c–e). These findings suggest that FGF21 loss causes cardiac maladaptation and a significant loss of endurance, leading to resistance to functional improvement and reduced performance, which aligns with the findings showing increases in serum FGF21 after both acute and chronic exercises in humans and mice.

Cold exposure increases heat production and, concomitantly, HR to maintain body temperature and blood flow. This heightened metabolic demand can strain heart’s energy influx, leading to potential functional inefficiency. While FGF21 promotes cold tolerance by activating brown and beige adipose tissues through induced expression^63^ (supplementary Fig. 6k), its effects on cardiac function during cold conditions are unclear. Telemetry revealed that, unlike WT mice, *Fgf21*-null mice presented elevated basal body temperature under normal conditions (supplementary Fig. 6f) but experienced a significant, progressive drop in core body temperature during the initial 24 h of exposure to 4 °C, indicating early cold intolerance (Fig. 2n). After 24 hours of fasting to reduce energy reserves (supplementary Fig. 6g), cold exposure increased the HR in both the *Fgf21*-null and WT mice, but the HR was significantly lower in the *Fgf21*-null mice than that in the WT mice (Figs. 2o and 1i, j, supplementary Fig. 6h) or under conditions without prefasting (not shown). This was accompanied by significant decreases in core body temperature and ambulatory movement (Fig. 2p, q, supplementary Fig. 6i, j). These findings suggest significant cardiac inefficiency under cold and energy stress without FGF21, along with decreased body temperature and physical activity.

**Fig. 3:**
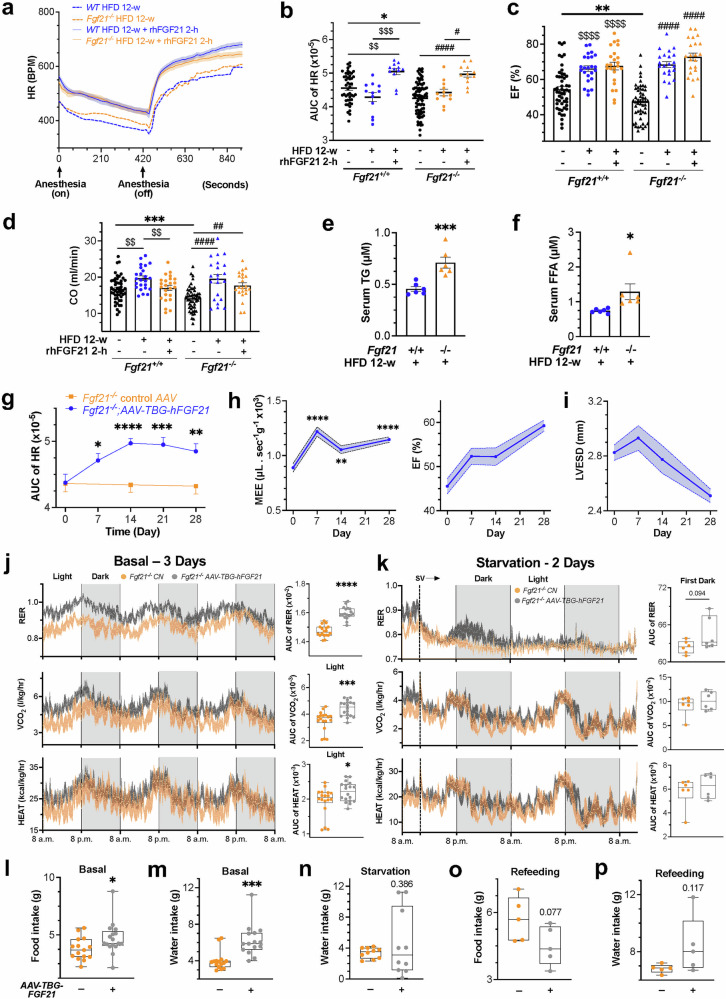
FGF21 restoration improves heart dysfunction under stress by promoting mitochondrial energy flux and macronutrient flexibility. **a** Effects of increased energy flux via a high-fat diet (HFD) and rhFGF21 on heart rate (HR) in *Fgf21*^*-/-*^ (n = 12) vs WT (n = 12) mice. w, week. See Fig. S7a. **b** Area-under-curve (AUC) analysis of HR changes in a. See Fig. S7b. **c**, **d** Changes in EF and CO in the same groups as in a. See Fig. S7c. **e**, **f** Changes in serum triglycerides (TG) and free fatty acids (FFA) in *Fgf21*^*-/-*^ (n = 6) vs WT (n = 6) mice as in a, as well as after 3-week rhFGF21 treatment. For other hepatic and serum metabolic parameters, see Fig. S8a–g. For the HFD effects on cardiac mitochondria, see Fig. S8h. For glucose intolerance status, see Fig. S8i, j. **g** Time courses of the effects of FGF21 restoration on HR via AAV-mediated overexpression in *Fgf21*^*-/-*^ (n = 12) vs AAV control (n = 12) mice. See Fig. S9a for experimental scheme and Fig. S9b for HR excursion curves. **h**, **i** Time-dependent changes in MEE, EF and LVESD after FGF21 restoration in *Fgf21*^*-/-*^ mice (n = 31). See Fig. S9c–e. **j**, **k** Energy expenditure (RER, VCO_2_, HEAT) by indirect calorimetry under basal conditions, after 48 hours of starvation, and after 24 hours of refeeding in the mouse groups as in **g**. See Fig. S10a for experimental design and Fig. S10b–i for other analyses. For changes in energy expenditure parameters between the *Fgf21*^*-/-*^ and WT mice under similar conditions, see Fig. S11a–d. CN, control vector. SV, starvation. Left, excursion curves. Right, AUC of the respective curve. The AUC plot without a title indicates total (light + dark) AUC. **l** n = 18 per group. **m** n = 6 per group. **l**–**p** Changes in food intake and water consumption in the same groups and conditions as in **j**–**k**. Data are means ± s.e.m.s; (**e**–**p**) two-tailed unpaired Student’s t test; (**a**–**d**) ordinary one-way ANOVA followed by Tukey’s test

FGF21 is increased in patients with obesity, which is detrimental to cardiovascular health. However, moderate HFD feeding could provide an energy-rich metabolic milieu for cardiac energetic functions. Following 12 weeks of HFD, the reduced HR from FGF21 loss under normal or fasting conditions normalized and became as sensitive to FGF21 replenishment as in WT mice (Fig. 3a, b, supplementary Fig. 7a, b). Interestingly, HR increased significantly during the energy-consuming awakening phase in FGF21-depleted mice but decreased drastically during the ‘sleep’ phase in WT mice, indicating an effect of fat-centric catabolism that requires more oxygen. Overall, HR tended to decrease in WT mice but increase in *Fgf21*-knockout mice, resulting in a similar profile. Echo parameters also improved and became more responsive to rhFGF21 treatment (Fig. 3c, d, supplementary Fig. 7c). Despite this, HR remained lower in *Fgf21*-null mice toward the end of the awakening phase, suggesting that FGF21 loss-caused defects are predominantly of energy origin, which can be adaptively masked by fat indulgence, rendering energy self-sufficiency. However, some cardiac defect(s) may persist due to long-term adaptation. Notably, *Fgf21*-null mice exhibited greater sensitivity to rhFGF21 treatment for improving EF, FS, LVESD, and LVEDD. The changes in CO were similar in both genotypes, but the overall changes were more significant in the knockout mice. These cardiac function changes concurred with mild changes in the relevant systemic metabolic parameters (Fig. 3e, f; a more detailed result description in supplementary Fig. 8). Together, these findings suggest that a moderate HFD affords heart energy self-sufficiency, masking mitochondrial defects and overcoming FGF21 deficiency-induced energy insufficiency. This finding underscores the essential role of energy supply, especially from FFAs, in maintaining heart functionality. Although FGF21 may become dispensable when the energetic threshold is met, cardiac functions could be further enhanced by rhFGF21, which is consistent with FGF21’s primary role in promoting lipid catabolism. The enhanced fat conversion into mechanical energy in the heart or muscle also accounts for FGF21’s antiobesity effects. Thus, a defective energy flux is fundamental to heart defects associated with *Fgf21* nullification across various stress conditions.

### Restoring FGF21 rectifies heart dysfunction by enhancing macronutrient flexibility and mitochondrial energy flux

To elaborate on the importance of cardiac energy flux regulated by FGF21, we restored FGF21 in *Fgf21*-null mice using a TBG promoter-driven minigene (supplementary Fig. 9a and g). This restoration resulted in time-dependent HR increases, peaking on day 14 post-AAV injection in mice fed *ad libitum* (Fig. 3g, supplementary Fig. 9b). Concurrently, MEE, EF, FS and CO increased, while LVESD and LVEDD decreased over 28 days (Fig. 3h, i, supplementary Fig. 9c–e), indicating improved cardiac energetic performance. Transmission electron microscopy (TEM) of heart sections showed that FGF21 restoration normalized mitochondrial crista density and interfibrillar array and corrected the vacuolation degeneration seen in the *Fgf21*-null hearts (Fig. 1k, supplementary Fig. 9f and 8h) or hearts with control vector (not shown). To assess the associated systemic energy status, we measured energy expenditure and substrate utilization by indirect calorimetry in FGF21-restored, FGF21-deficient, and WT mice under basal, 2-day starved, and refed conditions (Fig. 3j–p, see a detailed result description in supplementary Figs. 10 and 11). FGF21 restoration via minigene overexpression significantly increased respiratory exchange ratio (RER) under normal basal conditions compared to *Fgf21*-null mice, and even exceeded values in WT mice, which typically have low circulating FGF21 levels (Fig. 3j, supplementary Fig. 11a). During 2-day starvation, FGF21-deficient mice exhibited a marked reduction in the RER to 0.7-0.8, which was lower than the >0.8 observed in WT mice and moderately lower than that in FGF21-restored mice, especially during the first dark phase (Fig. 3k, supplementary Fig. 11b). These data suggest that FGF21 deficiency induces a mitochondria-centric hypoenergy state characterized by energy incompetency, fuel inflexibility, impaired glucose utilization, and a shift toward fat and potentially ketone utilization, reducing overall energy flux capability and fast adaptation. Thus, FGF21 signaling facilitates macronutrient utilization and efficient energy expenditure, thereby supporting heart functionality.

### Loss of FGF21 results in a hypometabolic and glycolytic state with cardiac defects in pathways for mitochondrial energy flux

**Fig. 4:**
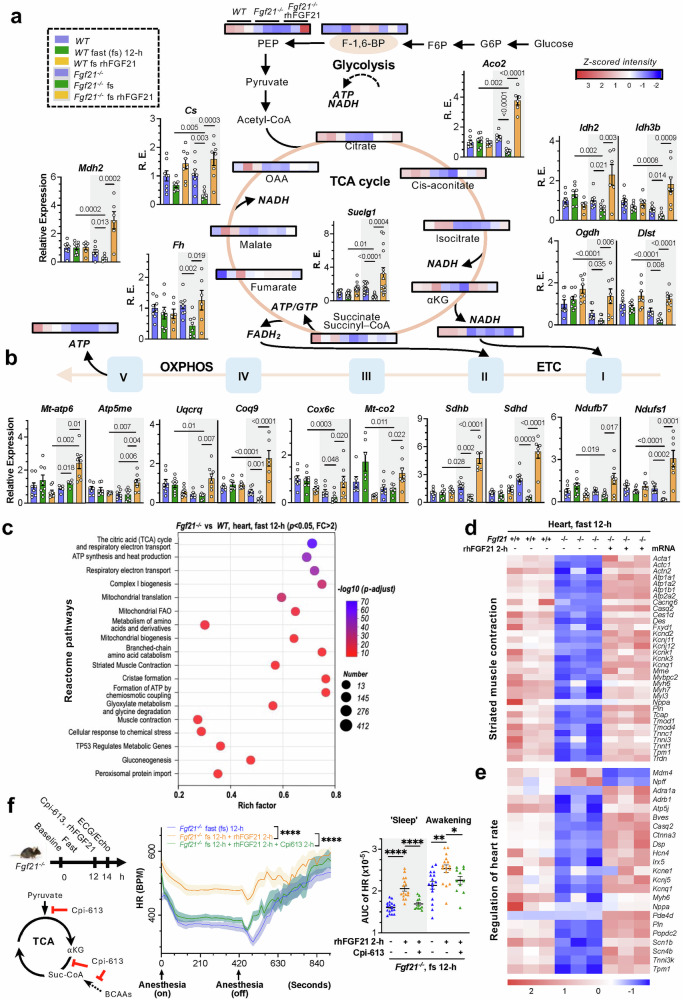
FGF21 deficiency induces a hypometabolic and mitochondrial hypoenergy state leading to cardiac dysfunction during fasting. **a**, **b** Changes in mitochondrial metabolite/energy flux and key metabolic pathway enzymes involved in the TCA cycle, ETC and OXPHOS in *Fgf21*^*-/-*^ (n = 5-15) vs WT (n = 6-14) mice after 12 hours of fasting (fs) or 2 hours of rhFGF21 treatment, as analyzed by targeted cardiac energy metabolomics and qRT-PCR. See Fig. S12a for a summary heatmap. **c** Transcriptomic and pathway enrichment in mitochondrial energy metabolism and cardiac function changes in *Fgf21*^*-/-*^ (n = 3) vs WT (n = 3) mice, with Reactome terms. For the GO-term and KEGG-term results, see Fig. S13a, b. For pathway enrichments in the Reactome term, GO term and KEGG term in *Fgf21*^*-/-*^ mice before and after rhFGF21 treatment, see Fig. S14. For mitochondrial biogenesis, TCA cycle, ETC complexes I-IV, and OXPHOS, see Figs. S15, S16d, and S17a. For 24-h fasting effects, see Fig. S16a–c. **d**, **e** Significant pathway defects associated with striated muscle contraction and heart rate regulation in the hearts of *Fgf21*^*-/-*^ (n = 3) vs WT (n = 3) mice and pathway normalization after 2 hours of FGF21 treatment (n = 3). For cardiac conduction, blood vessel diameter maintenance, and blood pressure regulation, see Fig. S13c–e and S17c. **f** Inhibiting the TCA cycle with Cpi-613 (1 mg/mouse, *i.p*.) reduced rhFGF21-promoted heart rate (HR) improvements in fasted *Fgf21*^*-/-*^ mice (same n = 6-16 mice per group). See Echo parameters in Fig. S17b. Data are means ± s.e.m.s; **c** two-tailed unpaired Student’s t-test; **a**, **b**, **d**–**f** ordinary one-way ANOVA followed by Tukey’s test. **a**, **f** images are generated in PowerPoint

To delve deeper into the regulation of cardiac energetics, we performed targeted intracardiac energy metabolomic analysis following a 12-h fast. Cardiac TPP, cis-aconitate, and AMP (another AMPK activator associated with AICAR and ATP) were reduced in the FGF21-deficient mice (Fig. 4a, supplementary Fig. 12a and Table 2), mirroring changes in the circulation (Fig. 1b, c). TPP is essential for several dehydrogenase complexes that feed into or constitute the TCA cycle. Correspondingly, a broad range of TCA cycle intermediates were reduced. The decreases in alpha-ketoglutarate and malate were also consistent with increases in serum glutamine and arginine-citrulline, likely due to their reduced entry into the TCA cycle (Fig. 1b, supplementary Fig. 1e). NADH and FMN (with succinate, precursors of FADH_2_), which feed into ETC complexes I and II respectively, decreased (Fig. 4a, supplementary Fig. 12a). Consequently, ATP, and less significantly, GTP levels were diminished. In contrast, F-1,6-BP, a central intermediate of glycolysis,^64^ was elevated, indicative of a shift toward glycolysis due to a reduced TCA cycle and respiration or at the expense of gluconeogenesis. NADPH, NAD^+^ and cAMP also tended to decrease, further suggesting a hypometabolic state. These findings align with indirect calorimetry data and serum SDL changes in *Fgf21*^*-/-*^ mice (Figs. 3j, k and 1b). Conversely, acute rhFGF21 treatment increased TPP, citrate, cis-aconitate, isocitrate, αKG, succinate, and OAA while reducing F-1,6-BP and GDP (Fig. 4a, supplementary Fig. 12a), with ATP and GTP trending upward, although longer treatment might be necessary (Fig. 4b, supplementary Fig. 12a). Taken together, FGF21 deficiency leads to a cardiac hypometabolic, glycolytic, and hypoenergetic state, while FGF21 signaling reinstates cardiac energy flux.

Furthermore, transcriptomic analyses (supplementary Fig. 12b–d, supplementary Table 3) revealed significant pathway alterations in cardiac mitochondrial structure, function, and energy conversion, including the TCA cycle, ETC, ATP synthesis, mitochondrial translation, MICOS complex, crista formation, and biogenesis, in fasted *Fgf21*-deficienct mice (Fig. 4c, supplementary Fig. 13a–b). At the tissue level, pathways/processes related to cardiac contraction rate and force, myofibril assembly, sarcomere organization, sarcoplasmic reticulum Ca^2+^ transport, muscle contraction, and blood circulation were altered most significantly (Fig. 4c–e, supplementary Fig. 13a–b). Notably, the FGF21-driven changes occurred without direct cardiac action, as indicated by negligible KLB expression^65^ and a lack of EGR1 induction (supplementary Fig. 12e). Heart rate regulation was notably affected, consistent with the observed HR alterations under various stress conditions. Pathways for cardiac conduction, blood vessel diameter maintenance, and pressure regulation were also significantly altered (supplementary Fig. 13c–e). At the metabolite level, changes were predominant in the catabolism of FFAs and branch-chain amino acids (BCAAs), mitochondrial/peroxisomal and proteasomal protein processing, and the biosynthesis of acetyl-CoA, acyl-CoAs and purine nucleotide bisphosphates. These findings are in accordance with the serum and cardiac metabolite profiles (Figs. 1a–c and 4a, supplementary Fig. 1c–e) and demonstrate cardiac versatility in the use of diverse energy substrates. These pathways were exclusively downregulated in fasted *Fgf21*-null mice but notably reinvigorated by acute rhFGF21 treatment (supplementary Fig. 14a–c). In addition, FGF21 signaling positively impacted pathways for mitochondrial calcium flux, membrane potential, vitamin and cofactor metabolism, substrate transport, heme (an ETC cofactor) synthesis, ROS production, and endothelial function (supplementary Fig. 14a, b, supplementary Fig. Table 3 - heatmaps not shown).

Confirmative qPCR analysis revealed that under normal conditions, FGF21 loss had limited effects on genes involved stepwise in TCA cycle, ETC and OXPHOS. However, a 12-h fast uniformly reduced their expression in FGF21-deficient but not WT mice, while rhFGF21 treatment reversed this effect (Fig. 4a, b, supplementary Fig. 15a–c). During prolonged fasting, this trend remained, although some genes lost response to *Fgf21* loss and rhFGF21 treatment (supplementary Fig. 16a, b, j). The mitochondrial biogenic factor PGC1a followed a similar trend (supplementary Fig. 16c). Notably, complex I (the entry site for NADH oxidation) and III/IV were the most affected (supplementary Fig. 13a, 14a, b and 15c). Correspondingly, cardiac NADH production was impaired in *Fgf21*-null mice but was recovered by acute rhFGF21 treatment (supplementary Fig. 16d), which was correlated with notable changes in cardiac ATP levels (Fig. 4a, supplementary Fig. 12a). Organelle biogenesis and ER/Golgi-associated vesicle transport were significantly impacted, highlighting FGF21’s role in energy substrate collection and flux (supplementary Fig. 17a, supplementary Table 3). Interestingly, changes in FGF21-responsive genes such as OXCT1, the SUCL complex, PDHA1, OGDH, ACO2, IDH3B, and ETC components (MT-CO2, COQ9, UQCRQ, SDHB/D and NDUFB7) are linked to mitochondrial deficiency syndromes manifesting cardiomyopathy and encephalopathy. Inhibiting DLD (an E3 component of pyruvate-, αKG- and BCAA-dehydrogenases) with Cpi-613, which undermines the TCA cycle, reduced rhFGF21’s efficiency for improving HR, EF and CO in fasted *Fgf21*-null mice (Fig. 4f, supplementary Fig. 17b). These findings suggest that FGF21 regulates TCA cycle to meet cardiac energy demand during energy stress.

Pathway enrichment revealed key susceptibilities to developing cardiomyopathies and ischemia due to FGF21 deficiency during fasting, which was potentially mitigated by rhFGF21 treatment (supplementary Fig. 13b and 14c). Under normal conditions, cardiac contraction genes showed minimal changes, but 12-h fasting disrupted *Tnni3* and *Myh7* expression (linked to cardiomyopathies), which was prevented by rhFGF21 replenishment (Fig. 4d, supplementary Fig. 17c). The levels of CKM, mitochondrial CKMT2, GATM, GAMT, and SLC6A8, which are involved in cardiac creatine synthesis and transport, exhibited similar alterations (supplementary Fig. 16e, f and 1d). GATM converts taurine and arginine into phosphagen taurocyamine, which, together with uridine, stabilizes the ETC and improves heart failure; all these metabolites were altered in FGF21-deficient serum. The levels of CDO1, CSAD, and SLC6A6, which are involved in cardiac taurine synthesis and import, were correlated with changes in TCA cycle/ETC and serum taurine levels (Fig. 1b supplementary Fig. 16g). The thiamine transporter SLC19A2 (linked to fulminant Beriberi) showed a similar pattern, correlating with changes in cardiac thiamine metabolism and serum thiamine (Fig. 1b, supplementary Fig. 16h, i). Unitedly, these findings suggest that FGF21 loss autonomously undermines cardiac mitochondrial energy metabolism, thereby impairing the energetic functions of the heart.

### FGF21 orchestrates cardiac catabolism of various substrates to ensure cardiac energy flexibility and sufficiency under stress

**Fig. 5:**
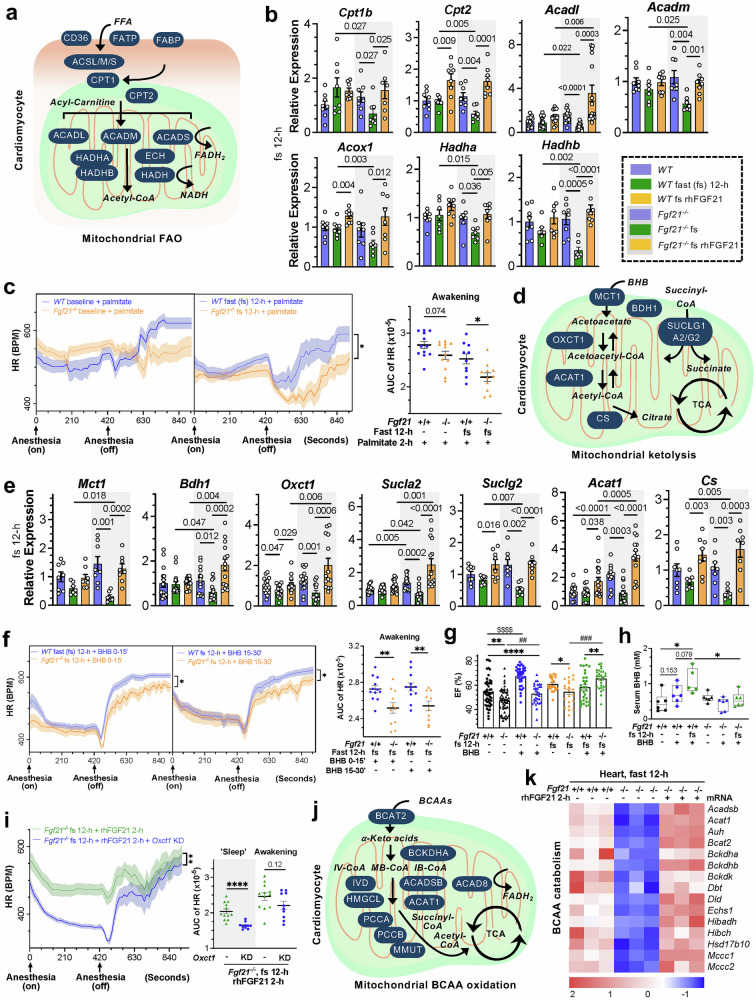
FGF21 promotes cardiac catabolic pathways for diverse metabolites to ensure cardiac energetic efficiency under stress. **a**, **b** Analysis of the expression of representative mitochondrial FAO pathway genes by qRT-PCR in the hearts of fasted *Fgf21*^*-/-*^ (n = 7-14) vs WT (n = 7-15) mice and with rhFGF21 treatment (n = 8-15). For transcriptomic enrichment of the FAO pathway, see Fig. S18a. For FAO under a 24-h fast, see Fig. S18b, c. For the metabolism of TG, phospholipids, and lipoproteins, see Fig. S18d, e. **c** Effects of palmitate supplementation (2 mmol per mouse, *i.p*.) on the heart rate (HR) of fasted *Fgf21*^*-/-*^ (n = 11) vs WT (n = 10) mice. See Fig. S19a for other related comparisons. **d**, **e** Changes in mitochondrial beta-hydroxybutyrate (BHB) ketolysis pathway determined by qRT-PCR in the hearts of fasted *Fgf21*^*-/-*^ (n = 7-15) vs WT (n = 7-14) mice and those with rhFGF21 treatment (n = 8-15). See Fig. S19b for transcriptomic enrichment of ketolysis and Fig. S19c for ketolysis after a 24-h fast. For utilization of other ketones, see Fig. S19d, e. **f** Effects of 1,3-butanediol monoester (BHB precursor, 10 mmol/mouse via tail vein) on HR in fasted *Fgf21*^*-/-*^ (n = 11) vs WT (n = 12) mice. See Fig. S20a for other related comparisons. **g** Effects of 1,3-butanediol monoester on EF in fasted *Fgf21*^*-/-*^ (n = 29) vs WT (n = 30) mice. See Fig. S20b for effects on CO. **h** Effects of 1,3-butanediol monoester on serum BHB levels in fasted *Fgf21*^*-/-*^ (n = 6) vs WT (n = 6) mice. See Fig. S20c for effects on serum FFA, TG, glucose, and total cholesterol. **i** Effects of cardiac *Oxct1* knockdown (KD) by AAV-mediated shRNA (2.5 ×10^11^ GC/mouse via tail vein) on rhFGF21-promoted HR improvement in fasted *Fgf21*^*-/-*^ mice (n = 10) vs those with control AAV (n = 11-12). See Fig. S20d for the knockdown efficiency and Fig. S20e, f for more detailed comparisons and Echo parameters. **j**, **k** Transcriptomic changes in branched-chain amino acid (BCAA) oxidation pathway in the hearts of fasted *Fgf21*^*-/-*^ (n = 3) vs WT (n = 3) mice and those with rhFGF21 treatment (n = 3). See Fig. S21b, c for detailed pathway changes under both 12-h and 24-h fasts, and Figs. S21a, S22d, e and S23a for amino acid metabolism and protein turnover. See Fig. S22a–c for leucine supplementation effects. IV-CoA, isovaleryl-CoA; MB-CoA, 2-methylbutyryl-CoA; IB-CoA, isobutyryl-CoA. Data are means ± s.e.m.s; (**c**, **f**, **i**) two-tailed unpaired Student’s t-test; (**b**, **e**, **g**, **h**, **k**) ordinary one-way ANOVA followed by Tukey’s test. (**a**, **d**, **g**) Images are generated in PowerPoint

To comprehend how FGF21 deficiency causes low energy in cardiac mitochondria, we further examined upstream energy substrate flux and utilization pathways. Under normal conditions, FGF21 loss had minimal effects on most cardiac FAO genes, except peroxisomal *Ehhadh*, *Hsd17b4*, and ER-residing *Hacd1/2* (linked to congenital cardiomyopathy) (Fig. 5a, b, supplementary Fig. 18a–c). Following a 12-h fast, FGF21 deficiency caused stepwise, uniform decreases in their expression levels, which became less pronounced with extended fasting likely due to diminished metabolite availability. Changes in lipid importer CD36 and the lipases PNPLA2 (ATGL) and LPL became more prominent after 24 h of fasting, indicating profoundly impaired lipid catabolism yet increased lipid reliance during extended energy crises (supplementary Fig. 18d). Conversely, acute rhFGF21 replenishment restored/upregulated the expression levels of these genes (Fig. 5b, supplementary Fig. 18a–d). Other genes related to TG, phospholipids, sphingolipids, peroxisome FAO, and plasma lipoprotein catabolism, including cardiac lipases for TG and phospholipids (*Plaat1/3*, *Pnpla8*, *Pla2g5/12a*, *Plbd1*, but not *Pla2g4b*), were all downregulated following FGF21 loss during fasting (supplementary Fig. 18e), exclusively signifying a lipid hypometabolic state. Mutant PNPLA2 and PNPLA8 are causal for neutral lipid storage- or mitochondria-associated cardiomyopathies.^66,67^ Supplementing palmitate, which compensates for serum deficiency due to FGF21 loss (Fig. 1a), increased HR in both genotypes; however, after 12-h fasting, *Fgf21*^*-/-*^ mice showed much less effective recovery during the awakening phase (Fig. 5c, supplementary Fig. 19a), which was consistent with reduced FAO enzymes due to FGF21 deficiency. This response differs partly from HFD effects, suggesting a need for extended treatment or mixed FFA oxidation for better fuel flexibility.

**Fig. 6:**
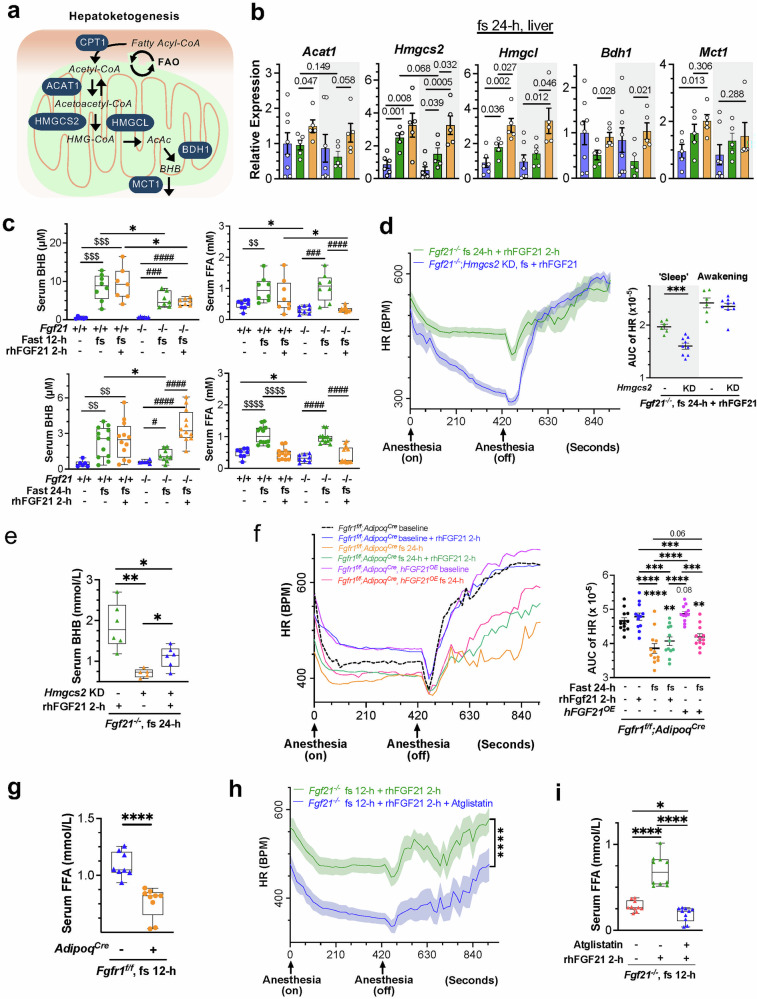
FGF21 promotes peripheral and transcardiac metabolic flux to meet cardiac energy needs during prolonged fasting. **a**, **b** Expression analysis of representative genes in hepatic ketogenesis pathway by qRT-PCR in *Fgf21*^*-/-*^ (n = 5) vs WT mice (n = 5), under basal, 24-h fast, and acute rhFGF21 treatment (n = 5) conditions. For the response to rhFGF21, see Fig. S25a. For the 12-h fast effects, see Fig. S25b. The bar symbols follow those in Fig. 5b. For pathway changes in FFAs sourced from WAT lipolysis, see Fig. S27a-S27c. **c** Changes in serum beta-hydroxybutyrate (BHB) and FFAs in *Fgf21*^*-/-*^ (n = 7-12) vs WT mice (n = 8-12) after fasting (both 12-h and 24-h) and rhFGF21 treatment (n = 7-12). For serum glucose and TG, see Fig. S26a. **d** Effects of hepatic *Hmgcs2* knockdown (KD) on rhFGF21-promoted improvement of HR in *Fgf21*^*-/-*^ mice subjected to a 24-h fast compared with control AAV (n = 6-9 per group). Right: AUC of HR excursion curves; see Fig. S26b, c for more details. For the Echo parameters, see Fig. S26d. **e** Changes of serum BHB levels upon hepatic *Hmgcs2* KD and rhFGF21 treatment in *Fgf21*^*-/-*^ mice after a 24-h fast. **f** Effects of adipocyte-specific ablation of *Fgfr1* (*Fgfr1*^*f/f*^*;Adipoq*^*CreERT*^) on the improvement of HR promoted by acute rhFGF21 treatment or AAV9-TBG*-hFGF21* mediated overexpression (*hFGF21*^*OE*^) in mice fasted for 24 hours (n = 11-12 per group). OE, overexpression. *hFGF21*, human FGF21 coding sequence. Right: AUC analysis, in which star symbols for *p*-values without underlines indicate a comparison to *Fgfr1*-ablated mice under normal conditions (baseline). See Fig. S28a for experimental scheme and S28b for the Echo parameters. **g** Changes of serum FFAs in *Fgfr1*^*f/f*^*;Adipoq*^*CreERT*^ mice. **h** Effects of the ATGL inhibitor Atglistatin (1.42 mg/mouse, *i.p*.) on the rhFGF21-promoted improvement of HR in *Fgf21*^*-/-*^ mice fasted for 12 hours (n = 11-12 per group). See Fig. S28c for experimental scheme and notes, Fig. S28d for more comparisons, and Fig. S28e for the Echo parameters. **i** Effects of Atglistatin on the rhFGF21-promoted increases in serum FFAs in *Fgf21*^*-/-*^ mice fasted for 12 hours (n = 9-10 per group), from which the heart draws FFA fuel. Data are means ± s.e.m.s; (**d**, **g**, **h**) Two-tailed unpaired Student’s t-test; (**b**, **c**, **e**, **f**, **i**) ordinary one-way ANOVA followed by Tukey’s test. (a) Image is generated in PowerPoint

Hepatic ketone bodies are crucial energy sources for fast/starvation. Ketone utilization was the most enriched pathway (supplementary Fig. 19b). Under normal conditions, FGF21 deficit minimally affected genes involved in cardiac beta-hydroxybutyrate (BHB) ketolysis and import. However, both the 12-h and 24-h fasts significantly reduced their expression exclusively in the *Fgf21*-null mice (Fig. 5d, e, supplementary Fig. 19c). Although not dramatic in individual genes, their cumulative effect within the enzymatic cascade can cause profoundly defective ketolysis. Contrarily, replenishing FGF21 restored their expression, indicating a critical role of FGF21 in regulating cardiac ketolysis throughout fasting, although prolonged fasting caused inflexibility in ketone utilization. Changes in GLO1 and HAGH (GLO2), which degrade acetone (or glycolytic methylglyoxal) via SDL to lactate, followed a similar pattern, which was consistent with altered serum SDL (Fig. 1b, supplementary Fig. 19b and 19d). The levels of AACS, which converts acetoacetate to acetyl-CoA, paralleled these changes (supplementary Fig. 19e). Notably, FGF21 loss downregulated, while rhFGF21 upregulated, cardiac ketogenesis (supplementary Fig. 19b).^68^ Whether this complements for early fasting ketolysis is an interesting question, as hepatic ketogenesis occurs during prolonged fasting (see Fig. 6b).^6^ Injection of 1,3-butanediol monoester (BHB precursor) improved HR in both the *Fgf21*^*-/-*^ and WT mice undergoing 12-h fasting (Fig. 5f, supplementary Fig. 20a). However, the HR of *Fgf21*^*-/-*^ mice still lagged behind that of the WT mice during the awakening phase. Improvements in EF and CO post-BHB injection were more pronounced in fasted *Fgf21*^*-/-*^ mice (Fig. 5g, supplementary Fig. 20b). Serum BHB levels remained low, while FFA and glucose were high post-injection, indicating increased reliance on exogeneous BHB due to impaired utilization of other metabolites in fasted *Fgf21*-null mice, compared to WT mice (Fig. 5h, supplementary Fig. 20c). Cardiac-specific knockdown of *Oxct1*, encoding the rate-limiting enzyme for BHB and acetoacetate ketolysis, significantly reduced the HR response to a 12-h fast (the median fasting point) and negated rhFGF21’s effects on improving cardiac function (Fig. 5i, supplementary Fig. 20d, e), especially in the resting phase that requires less oxygen, which was consistent with the improvements caused by exogenous BHB (Fig. 5f). Contrarily, EF, FS and CO increased while LVESD and LVEDD decreased, resisting normalization by rhFGF21, indicating elevated contractility to compensate for the severely depressed heartbeat (Fig. 5i, supplementary Fig. 20f). These findings suggest that FGF21 regulates cardiac ketolysis, a less oxygen dependent, efficient energy release process, to meet cardiac energetic demands during fasting.

Concertedly, FGF21 signaling enhanced catabolic pathways for various amino acids, particularly BCAAs, which enter the TCA cycle via succinyl-CoA, pyruvate/acetyl-CoA, and αKG (Figs. 4c and 5j, k, supplementary Figs. 13a, b, 14 and 21a). The expression of genes for BCAA catabolism, αKG and pyruvate dehydrogenases followed similar trends as those for FAO and ketolysis under both 12-h and 24-h fasts (Fig. 4a, supplementary Fig. 21b, c). Although a bolus injection of leucine alone had limited effects on HR, it reduced Echo parameters in fasted *Fgf21*^*-/-*^ mice and, notably, enhanced HR together with rhFGF21 during the awakening phase with normalized Echo parameters (supplementary Fig. 22a–c). The low efficiency may also indicate a need for a longer treatment or a BCAA mix. Interestingly, the proteasome, endopeptidases/peptidases, and lysosomal lytic activity, which recycle amino acids from unused or damaged proteins or organelles under fasting, were similarly affected (supplementary Figs. 22d, e and 23a). Consistent with serum citraconate changes, these findings highlight the critical role of FGF21 in catabolizing amino acids, *viz*., BCAAs, and proteins, to overcome the stress-induced cardiac hypometabolic/hypoenergy state.

Carbohydrates and glycogen are also key cardiac fuels, while nucleotide flux is essential for mitochondrial DNA synthesis, translation, turnover, and energy conversion. During fasting, FGF21 loss and restoration similarly impacted the cardiac metabolic flux of glucose/glycogen and nucleotides, alongside lipids, ketone bodies, and amino acids (detailed results are described in supplementary Fig. 23 and 24a-c). These changes correspond with changes in cardiac F-1,6-BP, VCO_2_/VO_2_, AMP, ATP, and NADH levels, as well as serum adenosine, AICAR, uridine and hypoxanthine levels (Figs. 1c, 3j and 4a, supplementary Fig. 12a). Moreover, during fasting but not at baseline, cardiac glycogen and phospholipids, but not triglycerides, were reduced significantly in *Fgf21*-null mice compared with WT mice, again indicating impaired fuel utilization. Following 2 h of rhFGF21 replenishment, cardiac triglycerides declined while glycogen and phospholipids moderately increased (supplementary Fig. 24d). Altogether, data from serum and cardiac metabolomes, pathway metabolons, and transcriptomes suggest that FGF21 loss induces a hypometabolic, hypoenergetic cardiac state, while FGF21 signaling promotes substrate flux to ensure efficient stress adaptation and optimal functional performance.

### FGF21 choreographies multiorgan metabolic flux to cope with dynamic cardiac energetic performance during prolonged fasting

The heart depends on peripheral metabolic organs for energy, with the liver being the source of ketones. FGF21 is a starvation hormone promoting hepatoketogenesis,^6^ but its role in directing hepatoketogenesis to cardiac ketolysis is unclear. rhFGF21 treatment acutely activated the hepatic marker gene *Egr1* (supplementary Fig. 25a). Without stress, FGF21 loss minimally impacted hepatic ketogenic and FAO gene expression (Fig. 6a, b, supplementary Fig. 25b–e). However, after a 24-h but not a 12-h fast, FGF21 deficiency reduced the expression of these genes and those for FFA transport and activation, suggesting defects in ketogenesis and FAO during protracted fast, which was correlated with spatially reduced cardiac ketolysis extending to 24 hours (supplementary Fig. 19c). Conversely, rhFGF21 treatment restored/enhanced their levels. Correspondingly, hepatic lipases and cofactors were highly induced by rhFGF21 only after 24 h of fasting (supplementary Fig. 25f), which is consistent with other reports.^6^ Given the significant impacts on cardiac ketolysis during both 12-h and 24-h fasts and the greater effects on cardiac FAO and BCAA catabolism during 12-h fasts (Fig. 5e, supplementary Fig. 21b, c), these findings suggest a spatiotemporal regulation of systemic energy flow to cardiac utilization by FGF21. The heart likely combusts available (intracardiac and blood-transcardiac) glucose, FFAs, and amino acids, but during prolonged fasting, it outsources ketones, which help reduce excessive oxygen use, as well as FFAs and amino acids.

Blood serves as a carrier and a reservoir for metabolites. Serum BHB, FFA, TG, and glucose levels exhibited complex, dynamic alterations during fasting and FGF21 alterations (Fig. 6c, supplementary Fig. 26a). Under normal conditions, FGF21 loss minimally affected serum BHB and TG but subtly reduced FFA and glucose. Fasting raised BHB and FFA levels while subtly reducing glucose without significantly changing TG. However, the increase in BHB was less pronounced in FGF21-deficient mice. rhFGF21 reduced FFAs and increased BHB in *Fgf21*^*-/-*^ mice, especially after a 24-h fast. Glucose was consumed earlier than FFAs and BHB during fasting, regardless of genotype or treatment, and TG levels remained low, particularly with rhFGF21 treatment. These findings highlight the specific role of FGF21 in the complex, dynamic mobilization of systemic metabolites, particularly FFAs and BHB. The differential changes in serum BHB levels underscore FGF21’s significant role in BHB flux during prolonged fasting. Knockdown of hepatic *Hmgcs2*, the rate-limiting enzyme in ketogenesis, prevented rhFGF21-driven HR improvements, especially during the inactivity phase, with significantly reduced serum BHB, in *Fgf21*-null mice after a 24-h fast (Fig. 6d, e, supplementary Fig. 26b–d). EF, FS and CO showed compensatory changes but did not reach the levels shown in rhFGF21-treated *Fgf21*-null mice (supplementary Fig. 4c–g). Interestingly, these change patterns, particularly those of HR, resemble those of cardiac *Oxct1* deficiency (Fig. 5i, supplementary Fig. 20e). These findings support the role of FGF21 in regulating a systemic metabolic axis from hepatic ketogenesis to meet cardiac ketolysis, sustaining cardiac energetic function during extended stress.

WAT is the primary FFA source during prolonged fast, matching the timing of hepatic ketogenesis and serum BHB elevation 24 hours postfast (Fig. 6b, c, supplementary Fig. 25f).^6^ Data from analyzing pathway gene expression involved in WAT lipolysis indicate that FGF21 promotes WAT lipolysis to meet hepatic ketogenesis under prolonged fasting (detailed description of the results in supplementary Fig. 27a–c).^6,69^ Interestingly, during a 24-h fast, FGF21 loss minimally affected BAT lipolysis, while rhFGF21 suppressed it (supplementary Fig. 27d), suggesting that under thermoneutral fasting conditions, FGF21 conserves lipid energy by limiting nonshivering thermogenesis in BAT.

To assess the impact of WAT lipolysis on heart energetic function, we generated an adipocyte-specific *Fgfr1* ablation (*Fgfr1*^*f/f*^*;AdipoQ*^*Cre*^) model. Loss of adipocyte FGFR1 had little effect on HR under unstressed conditions compared to the floxed mice. However, a 24-h fast significantly reduced the heartbeat in FGFR1-deficient mice with decreased serum FFAs, similar to those in *Fgf21*^*-/-*^ mice. Notably, the deficiency negated the beneficial effects of both acute rhFGF21 treatment and hFGF21 overexpression on improving HR, EF, CO, LVEDD, LVESD, and CO and serum BHB (Fig. 6f, g, supplementary Fig. 28a, b), suggesting a functional link from WAT to heart that is regulated by the FGF21-(adipose) FGFR1 signaling axis in response to prolonged fast or starvation. To understand the contribution of lipolysis during early fast, e.g., via myocytic lipid droplets, the circulation system, and other organs collectively,^67^ we utilized an ATGL inhibitor after a 12-h fast, not a 24-h fast (Fig. 6h, supplementary Figs. 28c, 25f and 27b). Pretreatment with Atglistatin prevented rhFGF21 from improving HR in *Fgf21*-null mice (Fig. 6h, supplementary Fig. 28d). Echo parameters were elevated in a compensatory manner by Atglistatin but resisted changes caused by rhFGF21 (supplementary Fig. 28e), alongside persistent reductions in serum FFAs (Fig. 6i), indicating that HR is also impacted by early fasting lipolysis, which directly flows to the heart (not hepatic ketogenesis) and is controlled by FGF21. This result is consistent with the reduced signature carnitine esters, which are precursors of mitochondrial FAO (Fig. 1a). These findings support that energy flux axes from lipolysis to heart function are critically regulated by FGF21 in a spatiotemporal manner during energy crises. They also underscore the importance of lipids in providing continuous cardiac energy flow.

### FGF21 governs cardiac energy flux through the coordination of the LKB1-AMPK-mTOR energy regulatory pathways

**Fig. 7:**
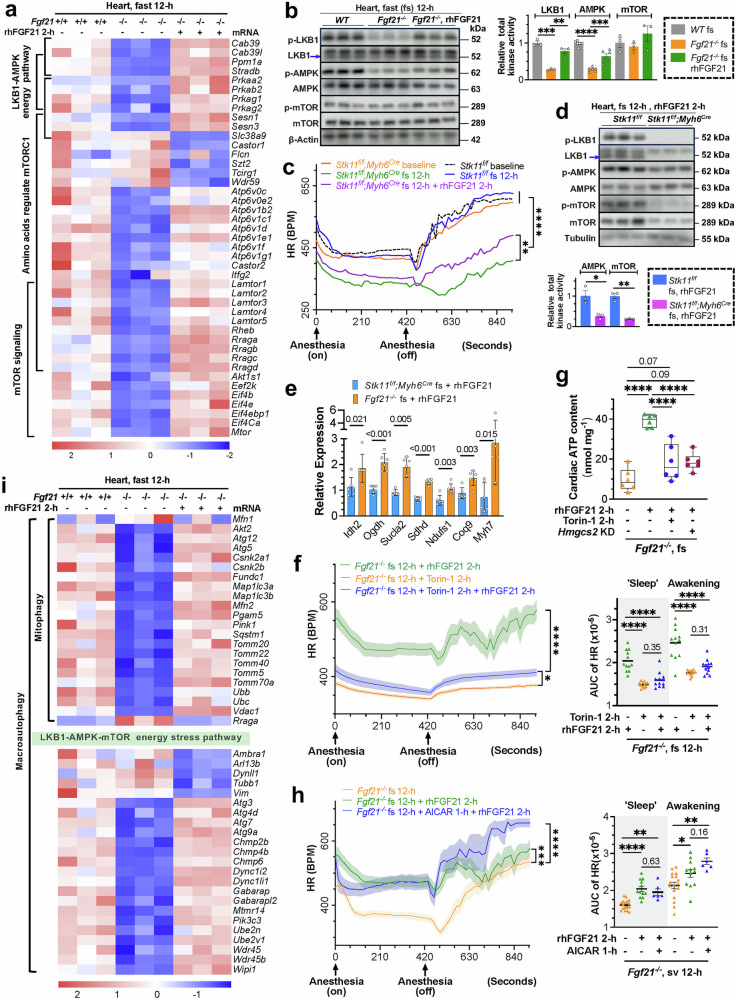
FGF21 orchestrates cardiac energy flux by engaging the LKB1-AMPK-mTOR energy stress pathways. **a** Transcriptomic enrichment of the LKB1-AMPK and mTOR energy regulation pathways in the hearts of fasted *Fgf21*^*-/-*^ (n = 3) vs WT (n = 3) mice and those with rhFGF21 treatment (n = 3). **b** Representative Western blot analysis of total kinase activities of cardiac LKB1, AMPK, and mTOR in the indicated mice (n = 3-6 for each group). Total kinase activity = (p-Kinase/Kinase) x (Kinase/Beta-actin), see also Fig. S29a. **c** Effects of cardiac-specific *Stk11* (LKB1) ablation (*Stk11*^*f/f*^*;Myh6*^*Cre*^) on HR. AUC analysis on the right. See Fig. S29b for experimental scheme, Fig. S29c for the AUC in the ‘Sleep’ and Awakening phases, and Fig. S29d for the Echo parameters. **d** Representative Western blot analysis of total kinase activities of cardiac AMPK, and mTOR in the indicated groups of mice (n = 3-6 for each group). See Fig. S29e. **e** Expression of representative genes involved in the TCA cycle, ETC, OXPHOS, and heart contraction in fasted *Stk11*^*f/f*^*;Myh6*^*Cre*^ and *Fgf21*^*-/-*^ mice 2 hours after rhFGF21 treatment. For changes in cardiac myofibrillar and mitochondrial structure, see Fig. S30a. **f** Effects of mTOR inhibitor Torin-1 (0.05 mg per mouse, *i.p*.) on rhFGF21-induced HR improvements in fasted *Fgf21*^*-/-*^ mice (n = 11-12 per group). See Fig. S29f, g for experimental scheme and total AUC. **g** Effects of Torin-1 and liver-specific *Hmgcs2* deficiency on the cardiac ATP content in acute rhFGF21-treated *Fgf21*^*-/-*^ mice (n = 6 per group) following 12-h and 24-h fasts, respectively. **h** Effects of the AMPK activator AICAR (1.25 mg/mouse, *i.p*.) (n = 6) on rhFGF21-induced HR improvements in fasted *Fgf21*^*-/-*^ mice (n = 12 and 17, respectively). See Fig. S30b-S30c for experimental scheme and total AUC, and Fig. S30d for the Echo parameters. **i** Transcriptomic enrichment of mitophagy, macroautophagy and associated LKB1-AMPK-mTOR energy stress pathways in the mice as in a. See Fig. S30e, f for mitophagy regulation by LKB1-AMPK-mTOR and autophagosome assembly. Data are means ± s.e.m.s; (d, **e**) Two-tailed unpaired Student’s t-test; (**a**–**c**, **f**-**i**) ordinary one-way ANOVA followed by Tukey’s test

Unbiased pathway enrichment identified LKB1-AMPK-mTOR as the top energy-linked regulatory pathways associated with FGF21’s cardiac effects during fasting (Fig. 7a), which were correlated with changes in serum AICAR, glucose, arginine and cardiac AMP, ADP and NAD^+^ levels (Fig. 1b, c, supplementary Fig. 12a and 26a), known activators of these kinases, and the observed hypometabolic state in fasted FGF21-deficient mice. A 12-h fast led to uniform reductions in cardiac expression of major genes in these pathways in the FGF21-deficient mice, which were reversed by rhFGF21 (Fig. 7a). Levels of cardiac pLKB1 (S431) and pAMPK (T172) were markedly reduced in *Fgf21*^*-/-*^ mice but were restored by rhFGF21 via phosphorylation and increased protein levels (Fig. 7b, supplementary Fig. S29a). While changes in pmTOR (S2448) were not statistically significant, the expression of its pathway components showed notable changes (Fig. 7a). These findings suggest that FGF21 may act through cardiac LKB1-AMPK and mTOR pathways, thereby impacting heart energetic function. Cardiomyocyte-specific knockout of *Stk11* (*Stk11*^*f/f*^*;Myh6*^*Cre*^) minimally affected HR under normal conditions but markedly reduced it after a 12-h fast, similar to FGF21 abolition. Notably, LKB1 loss abolished rhFGF21’s ability to acutely improve HR, EF, FS, and, to a lesser extent, LVESD and LVEDD, which coincided with persistent AMPK and mTOR pathway deactivation (Fig. 7c, d, supplementary Fig. 29b–e). Interestingly, chronic FGF21 overexpression promoted adaptive improvements in myocardial and mitochondrial structure (supplementary Fig. 30A). The cardiac TCA cycle and contractile gene expression remained lower than those of the *Fgf21*^*-/-*^ mice in response to rhFGF21 treatment (Fig. 7e). Pretreatment with the mTOR inhibitor Torin-1 severely reduced HR and nullified rhFGF21’s acute effects on improving HR in fasted *Fgf21*^*-/-*^ mice, alongside a reduction in cardiac ATP content similar to that caused by hepatic *Hmgcs2* loss (Fig. 7f, g, supplementary Fig. 29f-g). Echo parameters were not comparable because of large, nonadjustable HR differences. Contrarily, replenishing AICAR, which was deficit in circulation (Fig. 1c), restored heart function in FGF21-deficient mice subjected to fasting, without significant additive effects with rhFGF21 except at the later awakening phase (Fig. 7h, supplementary Fig. 30b–d), indicating that reactivation of AMPK effectively compensates for FGF21 deficiency. These findings support a critical role of the LKB1-AMPK-mTOR pathways in mediating FGF21’s effects on mitochondrial energy flux and heart rate maintenance, which is consistent with their known roles in energy stress response. However, given the negligible cardiac KLB expression and lack of EGR1 and c-FOS responses to rhFGF21, this effect is likely indirect, potentially through systemic factors or metabolites mobilized by FGF21.

Interestingly, major members of the V-ATPase, LAMTOR, and Rag GTPase families were altered significantly (Fig. 7a and i), which are involved in regulating nutrition sensing, lysosome function, and mitophagy to maintain nutrition recycling, mitochondrial health, and amino acid signaling to mTORC1 during energy crises. Macroautophagy, particularly PINK1-PRKN and Fundc1 mediated mitophagy, which clear damaged organelles, was defective in the hearts of FGF21-null mice but was normalized by rhFGF21 replenishment (Fig. 7i, supplementary Fig. S30e). These processes are known to be associated with the AMPK-mTOR pathways. This result aligns with significant defects in lysosomal lytic function, autophagosome formation (supplementary Fig. 22e and 30f), reduced serum N8-acetyl-spermidine (an autophagy regulator), and impaired mitochondrial ultrastructure, biogenesis, and energy flux due to FGF21 loss (Figs.1b, l and 4a-c, supplementary Fig. 14a), all of which were robustly reinstated by FGF21 restoration. These findings support that FGF21 promotes the mobilization of all sources of fuel under energy stress to maintain robust mitochondrial turnover, energy flux and cardiac contractility, which, at least in part, hinges on the energy-centric LKB1-AMPK-mTOR pathways.

## Discussion

**Fig. 8:**
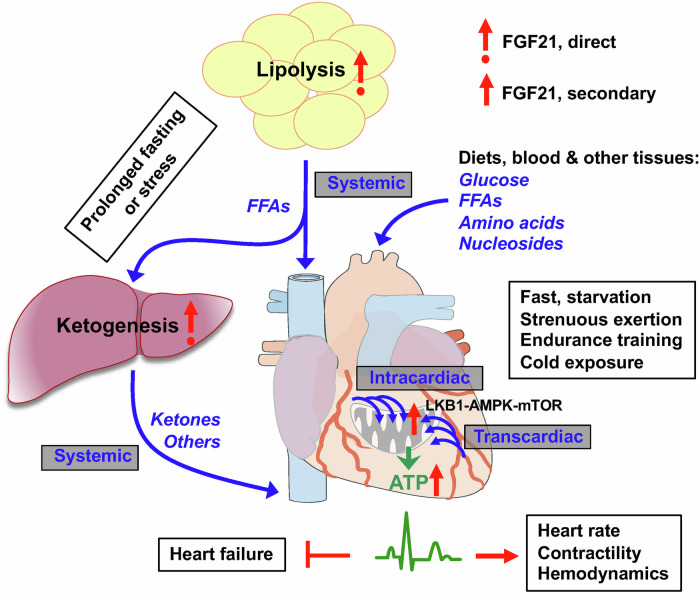
A mechanistic model for the regulation of heart energy allocation by energy stress hormone FGF21. The heart is an unrelenting bioengine that relies on a robust and uninterrupted influx of energy substates. Under physiologically relevant stressors, cardiomyocyte-derived or endocrine FGF21 acts to maintain heart rate, contractility, and hemodynamic stability by activating a dual energy flux system. Systemically, FGF21 directly promotes lipolysis in white adipose depots, releasing free fatty acids (FFAs) for liver uptake or direct transcardiac uptake via intracardiac microvascular circulation. It also promotes hepatic fatty acid oxidation and subsequent ketogenesis, supplying ketones for intracardiac ketolysis during prolonged fasting or starvation. These interorgan substrate mobilization effects ensure intracardiac energy substrate sufficiency. Locally, FGF21 signaling promotes transcardiac and intracardiac flux of various substrates for oxidative utilization, as well as cardiac mitochondrial biogenesis and respiration (the TCA cycle, ETC and OXPHOS), ensuring intracardiac ATP sufficiency. These processes/effects are mediated by the LKB1–AMPK and mTOR pathways and occur secondary to FGF21’s systemic actions. By coordinating these dual fuel systems, FGF21 signaling prioritizes incessant, robust intracardiac ATP flux, and thereby, cardiac energetic efficiency, particularly under stress. Thus, FGF21 is the first-known signaling factor for prioritizing cardiac energy needs and functional efficiency via a novel two-strata flux system. The image is generated in PowerPoint

Mitochondrial dysfunction and energy deficits are among the known causes of cardiomyopathies, heart failure, and other cardiovascular or tissue-specific diseases.^1,2^ Understanding mitochondrial function and energy flux regulation in the heart is key to uncovering underlying mechanisms and designing effective therapies. Here, we present comprehensive evidence that a spatiotemporally stratified, two-front energy flux system governed by FGF21 is an essential mechanism for ensuring cardiac energy priority and energetic efficiency. Locally, FGF21 is obligatory for robust cardiac mitochondria function, promoting intracardiac utilization of diverse metabolites and driving cellular and mitochondrial respiration, although likely in a secondary mode of action, to ensure cardiac ATP sufficiency. Systemically, FGF21 is crucial for interorgan substrate mobilization and transcardiac flux via circulation from major metabolic tissues such as the liver and adipose tissues, ensuring cardiac energy substrate sufficiency. FGF21’s cardiac action is likely mediated indirectly through nutrient-sensing LKB1-AMPK and mTOR pathways, which are activated by the increased influx of metabolites such as AMP, AICAR, arginine, and leucine under energy stress and FGF21 regulation. By coordinating the dual-fuel processes, FGF21 sustains incessant, robust intracardiac ATP flux, ensuring cardiac energetic function, particularly under stress (Fig. 8). Thus, our findings identify FGF21 as a first-known signaling factor for prioritizing cardiac energetics via an integrative two-strata flux system.

The heart is perpetually the most energy-demanding organ, which depends on clustered mitochondria for continuous ATP flux, a crucial intracardiac event. Untargeted plasma metabolomic, targeted cardiac energy metabolomic and transcriptomic analyses revealed that FGF21 is essential for this mitochondria-centric event. Whole-body loss of FGF21 results in compromised cardiac mitochondria, exhibiting intrafibrillar disarray, dilation, reduced mtDNA copy number, cristae vacuolation, and critical reductions in each catalytic step of the TCA cycle, ETC and OXPHOS. This loss also compromises each step of intracardiac catabolic pathways for FFAs, ketones, amino acids, glucose/glycogen, and even nucleosides, resulting in insufficient flux to the TCA cycle and ETC for ATP production. Consequently, the heart enters a hypometabolic, glycolytic, hypoenergetic state with compromised cardiac function efficiency, including reduced heart rate (an indicator of overall performance), contractility, and hemodynamic function. Despite normal heart development except for minor cardiac dilatation, the unhealthy mitochondria and energy insufficiency render the heart vulnerable and intolerant to even physiologically relevant stressors like fasting, starvation, strenuous exertion, endurance training, and cold. In contrast, treatments with both acute and chronic FGF21, but not metabolites alone, rejuvenate mitochondria and normalize/enhance heart energetic efficiency. Several studies highlight the cardioprotective effects of FGF21 against pathological insults and suggest several disparate mechanisms such as reducing lipid accumulation, glycolipotoxicity, oxidative damage, inflammation, fibrosis, and apoptosis, while promoting mitochondrial health.^24–27^ Our findings heretofore unify these mechanisms, advocating for a underlying physiological mechanism in which FGF21 acts as a determining factor for mitochondrial fuel sufficiency and cardiac energetic efficiency. All intracardiac pathways impacted by loss- and gain-of-function FGF21 revive around mitochondrial biogenesis, metabolite and intermediate flux, and energy generation, which aligns with the regulation of heart rate, striated muscle contraction, conduction, and hemodynamic performance. Our study thus elucidates why FGF21 is a key marker of mitochondrial damage in muscle and neural diseases,^7,10–12^ highlighting FGF21’s central role in orchestrating metabolite flux and mitochondrial function essential for cardiac efficiency and underscoring its therapeutic potential for these conditions. This finding also implies that training under a faulty FGF21 signal would fail to achieve the strength-building and longevity benefits.

Although the heart is endlessly energy dependent, it lacks significant reserves and relies on systemic and transcardiac energy substrate flux, especially under prolonged stress. Ensuring sufficient energy allocation to the heart is critical for survival, health and lifespan; however, its regulation is not clearly understood. Previous studies have highlighted FGF21’s roles in promoting hepatic ketogenesis and adipose tissue lipolysis during fasting and reducing plasma glucose, FFAs and TG in metabolic diseases.^5,6,9,30,70^ Unlike BAT, an energy consumer that benefits from FGF21’s action on mobilizing ketones and FFAs during cold exposure, the liver and WAT do not utilize these products themselves. The primary target of FGF21’s metabolic benefits remains undetermined. We demonstrate that during prolonged fasting, FGF21 loss impairs hepatic ketogenesis and associated hepatic lipolysis and FAO, whereas FGF21 replenishment restores ketogenesis and activates adipose tissue lipolysis. Blocking hepatic ketogenesis or (FGFR1-mediated) adipose lipolysis directly impairs rhFGF21’s ability to restore heart rate and contractility in FGF21-deficient mice, suggesting the obligatory role of FGF21 in regulating energy provision from the liver and adipose tissue to the heart during fasting/starvation. Furthermore, FGF21-regulated interorgan and transcardiac crosstalk occurs in a spatiotemporal manner. In the FGF21-deficient intracardiac milieu, glucose is consumed early, with continuous FAO, ketolysis, and amino acid oxidation, which are attenuated chronologically with fasting. FGF21 facilitates hepatic ketogenesis and adipose lipolysis only during prolonged fasting, meeting intracardiac ketolysis and FAO when intracardiac and plasma fuels are gradually depleted, thereby ensuring a continuous TCA cycle and efficient respiration. Interestingly, FGF21 inhibits BAT thermogenesis under prolonged fasting, strongly suggesting a specific role in ensuring energy allocation for the heart. Furthermore, FGF21 enhances intracardiac proteasome and lysosomal activities, mitophagy, and macroautophagy, which recycle unused or damaged proteins and organelles to stabilize the fuel supply and maintain the energetic performance of the heart under stress. These effects are, at least in part, mediated by the LKB1-AMPK-mTOR energy stress response pathways and aligns with the significant longevity effect of FGF21.^71^ Our findings position FGF21 as a key regulator in prioritizing systemic, transcardiac, and intracardiac substrate-energy flux, optimizing cardiac energetic efficiency under stress. This highlights the heart as the primary target of the benefits of FGF21, surpassing other high-energy organs like BAT, skeletal muscle and the CNS. Unlike skeletal muscle and the brain, which minimize energy consumption during rest and sleep, the heart continuously maintains its high-energy demand. Even under cold conditions, it is required to distribute BAT-generated heat throughout the body, ensuring survival. On the other hand, promoting fat/energy conversion to cardiac mechanical force may partly explain FGF21’s antiobesity or leptogenic effects under HFD challenge, complementing BAT-mediated heat dissipation. Of note, the physiological role of FGF21 in supporting heart rate and cardiac performance becomes evident under high-energy stress coupled with limited reserves rather than during basal, mild or energy-sufficient pathological state. Nonetheless, pharmacological FGF21 may still be effective under such conditions or in the presence of FGF21 resistance. Although its transcardiac and intracardiac action is likely indirect rather than through direct receptor-mediated signaling in cardiomyocytes or mitochondria, the coordination of these dual energy flux pathways represents a central physiological mechanism for sustaining cardiac performance and systemic energy balance under stress.

In conclusion, our study reveals a novel two-strata energy flux system pivotal for cardiac energetic efficiency, comprising intracardiac energy flux and systemic-transcardiac metabolite flux. FGF21 serves as a master regulator, strategically prioritizing fuel allocation to cardiac mitochondria in a spatiotemporally orchestrated manner under energy stress. Our findings unify the current understanding of FGF21’s metabolic roles and physiological targets, leveraging transformative insights into heart physiology, metabolic flux, and mitochondrial biology, which have profound strategic implications for (1) developing pharmacotherapies targeting cardiac diseases associated with mitochondrial and energy deficiencies, alongside MASH, hyperlipidemia, and obesity,^22,23^ and (2) designing caloric restriction and physical training interventions to improve physical strength, fitness, aging, and longevity.

We identified a previously unrecognized role of FGF21 in maintaining critical cardiac function under stress. While the heart is the primary target, although mechanistically indirect, owing to its omnivorous, relentless demand for energy, our results show that FGF21 also affects other mitochondria-rich, energy-intensive tissues, such as skeletal muscle, warranting further investigation. We observed abnormal serum serotonin and choline levels but no significant involvement of the sympathetic/parasympathetic or adrenergic systems, suggesting the need to further examine the role of serotonin. Similarly, dissecting the contributions of systemic versus intracardiac FGF21 will be insightful. The heart mitochondria act like a furnace, rapidly burning upon fuel infusion, while upstream FGF21 works to promote shoveling of the fuel from systemic and local sources. Elucidating the systemic-to-local flux pathways for each class of substrate would require targeted stable isotope tracing. Moreover, the intricate roles of the LKB1, AMPK and mTOR energy stress pathways in mediating FGF21-regulated cardiac energy flux, without the participation of cardiac FGFR-KLB, remains to be fully understood. Many FGF21-affected genes are p53 targets, raising intriguing questions about the central downstream transcriptional mechanism(s). Our study also suggests intracardiac ketogenesis during early fasting, warranting further investigation. Nevertheless, our experimental system and novel assessment approaches offer a strong platform for these investigations. Finally, we advocate for accelerating clinical trials of FGF21 mimetics for conditions related to mitochondrial and energy deficiencies.

## Materials and methods

**KEY RESOURCES TABLE**

**Table:** 

REAGENT or RESOURCE	SOURCE	IDENTIFIER
1. Antibodies		
Rabbit anti-Phospho-AMPK alpha (Thr172)	Cell Signaling Technology	2535, RRID: AB_331250
Mouse anti- AMPK alpha 1	Santa Cruz Biotechnology	sc-130394, RRID: AB_2169710
Mouse anti-Phospho-mTOR (Ser2448)	Proteintech Group	67778-1-Ig, RRID: AB_2889842
Mouse anti- mTOR	Proteintech Group	66888-1-Ig, RRID: AB_2882219
Mouse anti-Phospho-LKB1 (Ser431)	Santa Cruz Biotechnology	sc-271924, RRID: AB_10610759
Mouse anti-LKB1 (Ley 37D/G6)	Santa Cruz Biotechnology	sc-32245, RRID: AB_627890
Rabbit anti- PINK1	Proteintech Group	23274-1-AP, RRID: AB_2879244
Mouse anti- ATP5A1	Proteintech Group	66037-1-Ig, RRID: AB_11044196
Rabbit anti- OXCT1	Proteintech Group	12175-1-AP, RRID: AB_2157444
Rabbit anti- ATGL	Proteintech Group	55190-1-AP, RRID: AB_11182818
Rabbit anti-ACAT1	Proteintech Group	16215-1-AP, RRID:AB_2220210
Rabbit anti- SUCLG1	Proteintech Group	14923-1-AP, RRID: AB_2197177
Rabbit anti- Phospho-ERK1/2	Proteintech Group	28733-1-AP, RRID: AB_2881202
Rabbit anti-ERK1/2	Proteintech Group	11257-1-AP, RRID: AB_2139822
Rabbit anti- ACADL	Proteintech Group	17526-1-AP, RRID: AB_2219661
Mouse anti-Alpha Tubulin	Proteintech Group	66031-1-Ig, RRID:AB_11042766
Goat anti-mouse IgG, HRP-conjugated	Proteintech Group	SA00001-1 RRID: AB_2722565
Goat anti-rabbit IgG, HRP-conjugated	Proteintech Group	SA00001-2, RRID: AB_2722564
Goat anti-mouse IgG, CoraLite488-conjugated	Proteintech Group	SA00013-1, RRID: AB_2810983
Goat anti-rabbit IgG, CoraLite647-conjugated	Proteintech Group	SA00014-9, RRID: AB_2935614
2. Bacterial and virus strains		
pAAV9-TBG-mCherry-mir30shRNA(*mHmgcs2*)-WPRE	PackGene Biotech	N/A
pAAV9-cTnT-EGFP-shRNA(*mOxct1*)-WPRE	VectorBuilder	N/A
pAAV9-TBG-*hFGF21*-HA-IRES-EGFP-WPRE	VectorBuilder	N/A
pAAV9-TBG-shRNA-*Scramble*	VectorBuilder	N/A
pAAV9-cTnT-shRNA-*Scramble*	VectorBuilder	N/A
3. Chemicals, peptides, and recombinant proteins		
Recombinant human FGF21	This paper	N/A
Picro-Sirius red	Leagene Biotechnology	DC0041
Polyethylenimine (PEI)	Polysciences	23966
PrimeSTAR^®^ HS DNA Polymerase	Takara	R010A
DAPI	Solarbio	C0065
TRIzol	Invitrogen	15596026CN
Hieff Trans^®^ Liposomal Transfection Reagent	YEASEN	40802ES03
1,3-butanediol	MedChemExpress	HY-77490A
Sodium palmitate	Sigma-Aldrich	P9767
Atglistatin	MedChemExpress	HY-15859
AICAR	MedChemExpress	HY-13417
L-Leucine	MedChemExpress	HY-N0486
Torin 1	MedChemExpress	HY-13003
Devimistat	MedChemExpress	HY-15453
iFluor 488-Wheat Germ Agglutinin (WGA) Conjugate	Solarbio	I3300
Glucose monohydrate	Sigma-Aldrich	Y0001745
4. Critical Commercial Assays		
Modified Masson’s Trichrome Stain kit	Solarbio	G1346
EndoFree Maxi Plasmid kit	TIANGEN	DP117
HiScript III 1st Strand cDNA Synthesis Kit	Vazyme	R312
ChamQ Universal SYBR qPCR Master Mix	Vazyme	Q711
CheKine™ Micro Mitochondrial complex I Activity Assay Kit	Abbkine	KTB1850
CheKine™ Micro Mitochondrial complexⅡ Activity Assay Kit	Abbkine	KTB1860
CheKine™ Micro Mitochondrial complex Ⅲ Activity Assay Kit	Abbkine	KTB1870
CheKine™ Micro Mitochondrial complex Ⅳ Activity Assay Kit	Abbkine	KTB1880
Enhanced BCA Protein Assay Kit	Beyotime	P0010
Triglyceride assay kit	Jiancheng Bioengineering	A110-1-1
Nonesterified Free fatty acids assay kit	Jiancheng Bioengineering	A042-2-1
β-Hydroxybutyric acid (β-HB) Content Assay Kit	Solarbio	BC5085
Mouse phosphocreatine ELISA KIT	Biotopped	TOPEL30254
Phospholipid Assay Kit	CELL BIOLABS, INC	MET-5085
Liver/Muscle glycogen assay kit	Jiancheng Bioengineering	A043-1-1
Triglyceride assay kit	Jiancheng Bioengineering	A110-1-1
Mouse Fibroblast Growth Factor 21 (FGF21) ELISA Kit	JONLNBIO	JL22924
Human Fibroblast Growth Factor 21 (FGF-21) ELISA Kit	JONLNBIO	JL19322
5. Experimental models: Organisms/strains		
Mouse: C57BL/6 J	Beijing Vital River Laboratory Animal Technology	Strain code: 219
Mouse: *Fgf21*^*-/-*^	Original gift from Dr. Steven Kliewer	N/A
Mouse: *Fgfr1*^*flox/flox*^	Gift from Dr. Zhihfeng Huang	S-CKO-02414
Mouse: *Stk11*^*flox/flox*^	Cyagen Biosciences (Suzhou)	S-CKO-05368
Mouse: *Myh6*^*C*re^	Cyagen Biosciences (Suzhou)	C001009
Mouse: A*dipoQ*^*Cre*^	Cyagen Biosciences (Suzhou)	C001186
6. Recombinant DNA		
Oligonucleotides		
shRNA targeting sequence for mouse *Hmgcs2:* AGCCATGTCTGTCTACACGAAA	This paper	N/A
shRNA targeting sequence for mouse *Oxct1*: ACGGAAGGATGTCAGTAATCAA	This paper	N/A
Primers sequences for mouse genotyping	Table S4	N/A
Primers sequences for qRT-PCR	Table S5	N/A
7. Deposited data		
The raw data of cardiac RNA-Seq generated	This paper	PRJNA1189445
The raw of serum metabolomics generated	This paper	Table S1, MTBLS11726
The raw of cardiac energy metabolomics generated	This paper	Table S2, MTBLS11725
8. Software and algorithms		
GraphPad Prism 9	GraphPad	https://www.graphpad.com/scientific-software/prism/
ImageJ	NIH	https://imagej.net/Fiji
R package (v4.2.2)	The R Foundation	https://www.r-project.org
9. Other		
Standard rodent chow	Dyets	D100000
Rodent Diet With 60 kcal% Fat	Research diets	D12492

## Experimental model and subject details

### Mouse models

All animal procedures were conducted in compliance with protocols (wydw2022-0484 and wydw2022-0321) approved by the Institutional Animal Care and User Committee (IACUC) of Wenzhou Medical University and in accordance with National Institutes of Health (NIH, USA) guidelines. The mice were housed in a pathogen-free, temperature-controlled environment under a 12-h light/dark cycle, with free access to water and a standard rodent chow diet at 23 ± 3 °C and 30-70% humidity. Only age-matched male mice were used in the study. For blinding, the mice and samples were numbered independent of genotypes. The mice were sacrificed by cervical dislocation.

The mouse strain with a germline knockout of exons 1-3 of the *Fgf21* gene (*Fgf21*^*-/-*^) was generated on a C57BL/6 J background originally by Dr. Steven Kliewer’s group.^30^ Cardiomyocyte-specific knockout of *Lkb1* (*Lkb1*^*f/f*^*;Myh6Cre*) was achieved by crossbreeding the *Lkb1*^*f/f*^ line (#S-CKO-05368) and the *Myh6*^*Cre*^ (#C001009) line purchased from Cyagen Biosciences, Inc. (Suzhou, China). The exons 2-8 in the *Lkb1* gene allele were floxed. Adipocyte-specific knockout of *Fgfr1* (*Fgfr1*^*f/f*^*;Adipoq*^*Cre*^) was achieved by crossbreeding the *Fgfr1*^*f/f*^ line (#S-CKO-02414) and the *Adipoq*^*Cre*^ (#C001186) line, also from Cyagen Biosciences, Inc. These conditional knockout mouse lines were all generated on a background of C57BL/6 J via the CRISPER/Cas-mediated genome engineering approach. The genotyping of offsprings was performed by PCR amplification of genomic DNA extracted from their tails using the primers listed in Table S4. Experiments were conducted in *Lkb1*^*f/f*^*;Myh6*^*Cre*^ and *Fgfr1*^*f/f*^*;Adipoq*^*Cre*^ mice after fasted for 12 and 24 hours, respectively.

To generate the FGF21 restoration mouse model, 8-week-old *Fgf21*^*-/-*^ mice were intravenously injected with 100 μl of 2.5 × 10^11^ AAV virus containing AAV9-TBG-*hFGF21*-HA-IRES-EGFP-WPRE mini-gene (VectorBuilder, China) via tail vein, with an EGFP-only vector as the control. The coding sequence of full-length human FGF21 (hFGF21) with 209 aa starting with the first Met was used. An HA tag was inserted into the sequence after Ala-28 for expression validation by Western blotting or qRT-PCR analysis with a pair of primers F, 5’- CCATACGATGTTCCAGATTACGC and R, 5’-ATCTCCAGGTGGGCTTCTGT. Mice were monitored for heart function performance at days 0, 7, 14, 21 and 28. For indirect calorimetry, the mice were placed into metabolic cages one month after AAV injection.

To establish a compound mouse model with disrupted cardiac ketolysis, 8-week-old *Fgf21*^*-/-*^ mice were injected with 100 μl of 1.5 × 10^11^ AAV virus containing AAV9-nTnT-EGFP-shRNA(m*Oxct1*)-WPRE mini-gene (VectorBuilder, China) via tail vein, with an EGFP-only vector as the control. The shRNA target sequence was 5’-ACGGAAGGATGTCAGTAATCAA. Experiments were then performed 2 weeks post-injection after a 12-h fast.

To establish a compound mouse model with disrupted hepatic ketogenesis, 8-week-old *Fgf21*^*-/-*^ mice were injected with 100 μl of 1.5 × 10^11^ AAV virus containing AAV9-TBG-mCherry-mir30shRNA(m*Hmgcs2*)-WPRE mini-gene (PackGene Biotech, China) via tail vein, with the mCherry-alone vector as the control. The shRNA target sequence was 5’-AGCCATGTCTGTCTACACGAAA. Experiments were then performed 2 weeks post-injection after a 24-h fast.

To establish a mouse model of TCA cycle disruption, 8-week-old *Fgf21*^*-/-*^ mice were fasted for 12 hours and then injected with Cpi-613 (1.0 mg/mouse, *i.v*.) (MedChemExpress, USA). After 1.5 h, the mice were injected with rhFGF21 (1 mg/kg, *i.p*.). The functional experiments were then conducted 1.5 hours post-injection.

To establish a mouse model of adipose lipolysis disruption, 8-week-old *Fgf21*^*-/-*^ mice were fasted for 12 hours and then injected with Atglistatin (1.42 mg/mouse, *i.p*.) (MedChemExpress, USA). After 2 hours, mice were injected with rhFGF21 (1 mg/kg, *i.p*.). The functional analysis experiments were then conducted 2 hours post-injection.

To establish a mouse model with mTOR inhibition, 8-week-old *Fgf21*^*-/-*^ mice were injected with Torin-1 (0.05 mg/mouse, *i.p*.) (MedChemExpress, USA) followed by a 12-h fast. The mice were then injected with Torin-1 again at the same dose. After 2 hours, mice were injected with rhFGF21 (1 mg/kg, *i.p*.). The functional analysis experiments were then conducted 2 hours after the rhFGF21 injection.

To establish a mouse model with AMPK activation, 8-week-old *Fgf21*^*-/-*^ mice were fasted for 12 hours followed by an injection with rhFGF21 (1 mg/kg, *i.p*.). Two hours later, AICAR (1.25 mg/mouse, *i.p*.) (MedChemExpress, USA) was injected. The functional analysis experiments were then conducted 1 hour post-treatment.

To assess the effects of fatty acid supplementation on heart function, 8-week-old *Fgf21*^*-/-*^ mice were fasted for 12 hours and then injected with sodium palmitate (2 mmol/mouse, *i.v*.) (Sigma Aldrich, USA) via tail vein. Functional analysis experiments were conducted 2 hours post-injection for 15 min, compared with those of WT mice.

To assess the effects of amino acid supplementation on heart function, 8-week-old *Fgf21*^*-/-*^ mice were fasted for 12 hours and then injected with leucine (0.5 mg/mouse, *i.v*.) (Sigma Aldrich, USA). The functional analysis experiments were then conducted 2 hours post-injection for 15 min, compared with those of WT mice.

To assess the effects of ketone supplementation on heart function, 8-week-old *Fgf21*^*-/-*^ mice were fasted for 12 hours and then injected with 1,3-butanediol monoester (a racemic precursor of BHB) (10 mmol/mouse, *i.v*.) (MedChemExpress, USA). Functional analyses were then performed 2 hours post-injection for 30 min.

## Method details

### Untargeted serum metabolomics

#### Metabolite extraction

Fasting blood samples were collected in 5 ml Vacutainer tubes containing EDTA and centrifuged at 1500 g for 15 min at 4 °C. Aliquots of 150 μl of the plasma were stored at −80 °C until LC-MS analysis. For extracting metabolites, plasma samples were thawed on ice and 100 μl aliquots were mixed with 400 μl of cold methanol/acetonitrile (1:1, v/v) and vortexed to remove proteins. The mixture was centrifuged at 14000 g for 20 min at 4 °C. The supernatant was dried in a vacuum centrifuge. For LC-MS analysis, samples were redissolved in 100 μl acetonitrile/water (1:1, v/v) solvent, vortexed, and centrifuged at 14000 g for 15 min at 4 °C, and then injected into the LC system.

#### LC-MS analysis

TripleTOF 6600 Mass Spectrometer (SCIEX, USA) and Q Exactive HF-X Mass Spectrometer (Thermo, USA), both equipped with an electrospray ionization (ESI) source, were used for the analyses of untargeted serum metabolites. The mass spectrometers were coupled upstream with an Agilent 1290 Infinity UHPLC system (Agilent, USA) and a Vanquish UHPLC system (Thermo, USA), respectively. 2 μl of serum metabolite extract was injected via a 4 °C automatic injector into a HILIC column (ACQUITY UPLC BEH Amide, 150 mm×2.1 mm i.d., 1.7 µm, Waters, USA) maintained at 45 °C. In both ESI positive and negative modes, the mobile phases were as follows: phase A - 25 mM ammonium acetate and 25 mM ammonium hydroxide in water, and phase B - acetonitrile. The gradient was set as follows: 95% B for 0.5 min, linearly reduced to 65% in 6.5 min, to 40% in 1 min and kept for 1 min, then increased to 95% in 0.1 min, with a 3 min re-equilibration period. Quality control samples were set for each interval to evaluate the stability and repeatability of the system. The source conditions of ESI in both positive and negative ion modes were as follows: source temperature at 450 °C, ion source gas1 at 60, ion source gas2 at 60, curtain gas at 30 psi, ion source temperature at 600 °C, IonSpray voltage floating at 5500 V. MRM mode was used to detect ion pairs. The primary mass-to-charge ratio detection range was 80–1200 Da, with a resolution of 60000, scan accumulation time of 100 ms, scan accumulation time of 50 ms, and dynamic exclusion time of 4 s.

#### Data processing

The raw MS data were converted to MzXML files using ProteoWizard MSConvert before being imported into the XCMS software. For peak detection, the following parameters were used: centWave m/z at 10 ppm, peak width at c (10, 60), and prefilter at c (10, 100). CAMERA (Collection of Algorithms of MEtabolite pRofile Annotation) was used for annotating isotopes and adducts. In the extracted ion features, only variables with more than 50% of non-zero measurement values in at least one group were retained. Compound metabolite identification was done by comparing the accuracy of m/z value (<10 ppm) and MS/MS spectra to an in-house database established with authentic standards.

#### Statistical analysis

After sum-normalization, the processed data were analyzed using the R package (ropls) and subjected to multivariate data analysis, including Pareto-scaled principal component analysis (PCA) and orthogonal partial least-squares discriminant analysis (OPLS-DA). The robustness of the model was evaluated using 7-fold cross-validation and response permutation testing. The variable importance in the projection (VIP) value of each variable in the OPLS-DA model was calculated to indicate its contribution to classification. Student’s t-test determined the significance of differences between two groups of independent samples. Variables with VIP > 1 and *p* value < 0.05 were considered statistically significant.

### Targeted mitochondrial energy metabolomics

#### Metabolite extractions

This analysis covers important metabolites in the tricarboxylic acid cycle, glycolytic pathway, and oxidative phosphorylation. Heart samples stored at −80 °C were quickly cut without melting. 60 ± 5 mg of each sample was placed in a homogenizer tube together with 200 μl of cold methanol and 10 μl of 10 mM SUCCINIC ACID-D6 (internal standard). The samples were homogenized 3 times (20 s each) in a vertex and quenched by liquid nitrogen for 5 s. 400 μl of cold chloroform was added and mixed by vortexing for 30 s. The mixture was sonicated in an ice bath for 5 min and mixed by vortexing for 10 min. Then, 100 μl of deionized water was added and mixed by vortexing again for 10 min. The final mixture was centrifuged at 14,000 g at 4 °C for 20 min. 200 μl of the supernatant was stored at −80 °C until use or directly vacuum-dried at room temperature. The dried samples were reconstituted with 100 μl of acetonitrile-water solution (1:1, v/v), vortexed for 30 s, and centrifuged at 14000 g at 4 °C for 15 min. The supernatant was stored at −80 °Cuntil use or directly used for LC-MS analysis.

#### LC-MS analysis

The samples were separated using an Agilent 1290 Infinity LC ultra-high-performance liquid chromatography system (Agilent, USA). 2 μl samples were placed in an automatic sampler at 4 °C and injected into a HILIC column (ACQUITY UPLC BEH Amide, 150 × 2.1 mm i.d., 1.7 µm, Waters, USA) maintained at 35 °C, which was developed by the following mobile phases: phase A: 50 mM ammonium acetate aqueous solution and 1.2% hydroxide ammonia, and phase B: 1% acetone acetyl in ethanol, at a flow rate of 300 μl/min. The following mobile phase gradient was used: within 0–1 min, 70% B; within 1–10 min, B linearly changes from 70% to 60%; within 10–12 min, B changes from 60% linearly to 30%; 12–15 min, 30% B; and 15–22 min, 70% B. Quality control samples were set for each interval of experimental samples in the sample queue to detect and evaluate the stability and repeatability of the system. A mixture of standard compounds was used to correct retention time in the chromatography. Mass spectrometry was performed using a 5500 QTRAP mass spectrometer (SCIEX, USA) in both positive and negative ion modes. The 5500 QTRAP ESI source conditions were as follows: source temperature 450 °C, ion source gas 1 at 45, ion source gas 2 at 45, curtain gas at 30, IonSpray Voltage Floating at 4500 V. The MRM mode is used to detect the target ion pairs.

#### Data process

The Multiquant software was used to extract chromatographic peak area and retention time. The QCs were processed together with the biological samples. Retention times were corrected using standards of the target substances for metabolite identification. Metabolites in QCs with coefficient of variation less than 30% were denoted as reproducible measurements.

#### Data analysis

Statistical significance (*p* < 0.05) was determined using Student t test. Metabolites with *p* < 0.05, fold changes > 1.5 or fold changes < 0.67 were considered significantly changed. For hierarchical clustering, Cluster3.0 and the Java Treeview software were used. The Euclidean distance algorithm for similarity measure and average linkage clustering algorithm were selected for clustering.

### RNA-seq

#### RNA extraction

Total RNA was extracted from the heart using TRIzol® Reagent according to the manufacturer’s instruction. RNA samples quality was determined using 5300 Bioanalyser (Agilent, USA) and quantified using ND-2000 (NanoDrop Technologies, USA). Only the high-quality RNA samples (OD260/280 = 1.8 ~ 2.2, OD260/230 ≥ 2.0, RIN ≥ 6.5, 28S:18S ≥ 1.0, and >1 μg) were used to construct the sequencing library.

#### Library preparation and sequencing

RNA purification, reverse transcription, library construction and sequencing were performed at Shanghai Majorbio Bio-pharm Biotechnology Co., Ltd. (Shanghai, China) according to the manufacturer’s instruction (Illumina, USA). The heart RNA-seq transcriptome library was prepared following Illumina® Stranded mRNA Prep anLigation, with 1 μg of isolated total RNA. Messenger RNA was isolated using polyA selection by oligo(dT) beads and fragmented in fragmentation buffer. Double-stranded cDNA was synthesized using the SuperScript double-stranded cDNA synthesis kit (Invitrogen, USA) with random hexamer primers (Illumina, USA). The synthesized cDNA mix was subjected to end-repair, phosphorylation, and ‘A’ base addition according to Illumina’s library construction protocol. Libraries were size selected for cDNA target fragments of 300 bp on 2% Low Range Ultra Agarose gel, followed by PCR amplification using Phusion DNA polymerase (NEB) for 15 cycles. After quantification by Qubit 4.0, paired-end RNA-seq sequencing library was sequenced on the NovaSeq 6000 sequencer (2 × 150 bp read length) (Illumina, USA).

#### Quality control and read mapping

The raw paired end reads were trimmed and quality-controlled by fastp (v0.19.5) with default parameters. The clean reads were separately aligned to reference genome with orientation mode using HISAT2 (v2.1.0). The mapped reads of each sample were assembled by StringTie (v2.1.2) by a reference-based approach.

#### Differential expression analysis and functional enrichment

Differential expression analysis was performed using the R package DESeq2 (v1.38.0). DEGs with |log2FC| ≧ 1 and FDR ≤ 0.05 were considered significantly differentially expressed. Functional enrichment analysis, including GO, KEGG and Reactome, identified DEGs significantly enriched in GO term, KEGG pathways, and Reactome pathways at a Benjamini-Hochberg-corrected *p*-value ≤ 0.05 compared to the whole-transcriptome background. GO functional enrichment, KEGG pathways analysis and Reactome pathways analysis were carried out using Goatools (v0.6.5) and KOBAS (v2.1.1), respectively.

### Electrocardiogram

All mice were anesthetized with isoflurane gas inhalation and positioned supine on a thermostat. Three electrodes were attached to the upper limbs and right lower limb and connected to the PowerLab (High-performance data acquisition hardware, ADInstruments, USA) to acquire the ECG data. Mice were placed onto monitoring platform under anesthesia. Once a stable ECG wave was achieved, anesthesia administration was discontinued, and the mice were allowed to revive. At the start of the experiment, mice were given isoflurane gas at a rate of 2.4 mg/h, and ECG recordings were initiated to capture ECG data from the anesthetized mice. After 7 minutes of recoding under the ‘sleep’ phase, anesthesia administration was discontinued and ECG recording continued until the mice regained full consciousness and spontaneous movement, which lasted about 8 min. Heart rates of each experimental mouse were analyzed and plotted as XY curve and the corresponding area-under-curve (AUC) values were calculated in GraphPad 9 software.

### Echocardiogram

Mouse echocardiographic parameters were obtained using a Vevo 3100 system with a 30 MHz probe (Fujifilm VS, Canada). Mice were anesthetized with isoflurane for echocardiography. Left ventricular (LV) end systolic and diastolic diameters were calculated in 2D M-mode and the cardiac parameters were analyzed in a double-blind manner. For comparative analysis between different genotype groups or conditions, HR was maintained at equal levels unless otherwise specified (impossible to adjust to an equal HR level). LV fraction shortening (LVFS%) and LV ejection fraction (LVEF%) parameters were calculated using LV end-systolic diameter (LVESD) and LV end-diastolic diameter (LVEDD). Cardiac Output (CO) was calculated based on stroke volume (SV) and heart rate (HR). Left ventricular anterior wall end-systolic and end-diastolic dimensions (LVAW;s and LVAW;d), and left ventricular posterior wall end-systolic and diastoli dimensions (LVPW;s and LVPW;d) were also calculated in 2D M-mode and analyzed in a double-blind manner. In the apical three-chamber view, the pulsed Doppler sample volume was placed at the center of the mitral annulus to obtain the peak trans-mitral flow velocities in early diastole (E) and during atrial contraction (A), as well as trans-mitral early (E’) and atrial (A’) peak filling flow rates, which were used to calculate the E/A, E/E’, and E’/A’ ratios. Imaging data were processed with speckle tracking-based strain analysis on parasternal short- and long-axis B-mode loops using the Vevo Strain Software (Vevo LAB, Fujifilm VS, Canada).

### Cardiac efficiency

The assessment of cardiac efficiency is based on two calculations. In all conditions, the ejection fraction (EF) obtained from echocardiography analysis serves an accepted surrogate for cardiac efficiency, which is calculated as [(Stroke volume) / (End-diastolic volume) x 100%]. In some conditions, we also further calculated myocardial mechano-energetic efficiency (MEE) using the formula: (CO x HR^-2^), as described,^72^ which integrates cardiac functional output (CO) measured by echocardiogram relative to heart rate (HR) measured by electrocardiogram to better reflect energy efficiency of cardiac work. Since *exo vivo* Langendorff perfusion analysis of oxygen consumption in isolated heart would not recapitulate FGF21’s systemic effects, we use it as a simplified, noninvasive surrogate estimate of myocardial external efficiency.

### Telemetry (Heart rate, body temperature, and locomotive activity)

#### Animal preparation and HR E-Mitter implantation

All mice were anesthetized prior to preparation, with the ventral surface of the abdomen and the thorax to the area of the axilla shaved. Skin incisions were made along the abdominal midline below the diaphragm, approximately 1–2 cm in length. Mouse abdomen was then incised along the linea abla (the “white line” of fascia where the abdominal muscles join on the midline) for 2 cm. The E-Mitter implant (STARR Life Sciences Corp, USA) was positioned in the abdominal cavity along the sagittal plane and in front of the caudal arteries and veins but dorsal to the digestive organs. The negative and positive leads of the implanted device were secured in place within the right subclavian pectoralis superficialis and the left 8th intercostal pectoralis superficialis. The abdominal opening was closed with 5-0 Vicryl® absorbable sutures using a continuous interlocking stitch. The skin was then closed with 34-gauge stainless steel sutures using interrupted mattress stitches. Mice were allowed to recover for three weeks, with daily monitoring for the first week, then every three days for the following two weeks.

#### Acquisition and analysis of Telemetry data

After recovery, mice were placed individually into groups corresponding to their assigned receivers (E-Mitter Telemetry System, Starr Life Sciences, USA). Following a 3-day acclimation period, heart rate, body temperature, and locomotive activity were measured and recorded based on experimental group conditions. Telemetry data were then exported and analyzed using VitalView software.

### Transmission electron microscopy (TEM)

Longitudinal-section (along the direction of muscle filament) cubes ( ~ 1 mm^3^) were freshly isolated from mouse hearts and fixed in 2.5% glutaraldehyde solution at 4 °C overnight, followed by 1% osmic acid solution for 2 h. After wash with 0.1 M phosphoric acid buffer (pH7.0), the heart sample was dehydrated through a gradient of alcohol and acetone, then embedded in resin overnight. Embedded samples were sectioned into 70–90 nm ultrathin sections using LEICA EM UC7 microtome (Leica Microsystems, Germany), stained in lead citrate in dioxane acid solution, and dried overnight at room temperature. The sections were imaged using an FEI Tecnai G2 Spirit TWIN system (Thermo Fisher Scientific, USA). Heart structure, especially mitochondria cristae structure, shape, and array, in each sample was determined from a randomly selected pool of 10 to 20 image fields. The number of mitochondria, average mitochondrial cross-sectional area, and cristae number were evaluated with ImageJ.

Mitochondrial size was quantified as the cross-sectional area of individual mitochondria in randomly acquired TEM images (n = 10 per mice) from random heart sections (n = 11 mice per group).

### Mitochondrial respiratory complex activity

The catalytical activities of ETC complexes I-IV in heart mitochondria were measured using CheKine^TM^ kits according to manufacturer’s instructions (Abbkine, USA). Briefly, 0.1 gram of heart tissues were isolated freshly from model mice, rinsed, and homogenized in cold isolation buffer. Mitochondrial fraction was obtained by sequential centrifugation at 600 g for 5 min and 11,000 g for 10 min at 4 °C. The functional activity of each ETC complex was assessed in a 96-well plate using colorimetry on BioTek Synergy NEO2 (Agilent, USA) plate reader. A Bradford Protein Assay Kit (Beyotime, Shanghai, China) was used to quantify the mitochondrial samples. The results were expressed as U/g total protein.

### Mitochondrial DNA copy number

Total DNA was isolated from fresh or snap-frozen heart tissues. Relative mitochondrial DNA copy number was determined by qPCR using a primer pair for the mitochondria DNA-encoded gene *Mt-nd1*: F, 5’-GAGCTTTACGAGCCGTAGCC and R, 5’-GAATGGGCCGGCTGCGTA, yielding a 271 bp amplicon. This was normalized to the nuclear gene beta-2-microblobulin detected using a primer pair of F, 5’-ATGGGAAGCCGAACATACTG and R, 5’-CAGTCTCAG TGGGGGTGAA, yielding a 177 bp amplicon. The qPCR conditions and procedures followed those used for qRT-PCR in determining gene expression levels.

### Fast/starvation challenge

Mice were given *ad libitum* access to food during the feeding period. 12-h fast (fs) was initiated by removing food at the beginning of the dark cycle at 8:00 PM, which lasted for 12 hours overnight. 24-h fast was initiation at the beginning of the light cycle at 8:00 AM and continued through the dark cycle, which lasted for 24 hours. 48-h starvation (sv) followed the same time schedule but lasted for 2 days. Mice were given free access to water with bedding materials removed. Except refeeding, all experiments were conducted while still in fasting or starvation after the initial 12-h, 24-h or 48-h food-deprivation period.

### Acute involuntary physical exertion

To assess FGF21’ role in regulating heart function under acute strenuous activity or workload, involuntary running frequency and duration in model mice were monitored in conjunction with telemetry method as described above. Mice were individually housed in treadmill belt in each channel and forced to run by electric stimuli with defined exercise parameters (belt speed, pulse intensity, channel incline) controlled by computer in ZH-PT/5S Small Animal Treadmill (Zhenghua Biological Apparatus, China). Running time, distance and stimulus frequency were recorded and used for assessing exercise capacity, which was linked to real-time changes in heart rate, body temperature and other physical parameters.

### Endurance training

To assess FGF21’s role in regulating heart function under chronic body fitness exercise, model mice were grouped and placed on the belts for forced daily exercise for 2 hours for a total of 56 days in treadmill as described above. Telemetry, electrocardiogram and echocardiogram analyses were conducted on day 0, 7, 14, 28, and 56.

### Cold exposure

To assess FGF21’s role in regulating heart function under cold challenge, model mice were placed in a 4 °C cold room for 2 hours or 2 days in conjunction with telemetry as described above. Changes in heart rate, body temperature, and pedestrian locomotive activity were recorded. In addition, body temperature was also measured with rectum probe (KW-8 Multi-channel Body Temperature Monitor, KEW Basis, China).

### Indirect calorimetry

To assess the association of FGF21-regulated energy expenditure with heart energetic function under normal diet, 24-h fast, 48-h starvation, and 1-day refeeding conditions, telemetry-monitored *Fgf21*^*-/-*^ mice and *Fgf21*^*-/-*^ mice with AAV9-mediated overexpression of human FGF21 two-month post injection were placed in metabolic cages (PhenoMaster, TSE Systems, Germany) and allowed to acclimate for 2 days. Experiments were then conducted according to manufacturer’s instruction. Changes in RER, VO2, VCO2, HEAT, food intake, and water consumption were recorded and analyzed in PhenoMaster program.

### Metabolite measurements

Total lipids were extracted from ∼50 mg of liver. Triglyceride contents of the liver and plasma were measured using an L-type TG H triglyceride kit (Jiancheng Bioengineering, China). Plasma free fatty acids were measured using a NEFA C kit (Jiancheng Bioengineering, China). Plasma β-hydroxybutyrate concentrations were measured using a D-3-hydroxybutyric acid kit (Solarbio, China). Plasma D-glucose concentrations were measured using a Glucometer (Yuyue, China).

### Heart morphometric and structural parameters

Changes in cardiomyocyte size were assessed in the left ventricles. Paraffin-embedded cross-sectional heart tissue sections were deparaffinized, rehydrated, and incubated with iFluor-488 labeled WGA at 37 °C for 30 min. After wash with 1 x PBS, sections were sealed with DAPI-containing resinous medium. Tissue slides were visualized under a fluorescent microscope (Nikon, Japan). Images were analyzed using QuPath software. Only cross-sectional, near circular shaped cardiomyocytes were included in the analysis. The QuPath polygon tool was used to encircle the cells and determine the area.

Mouse body weight was measured prior to sacrifice. Freshly isolated heart was drained of blood and weighed on a micro-scale. Heart length was measured from atrium Sinistrum to Apex Cordis. Tibia length was also measured for each mouse. The corresponding values for each mouse were used to calculate HL/TL and HW/BW ratios.

### Histological analyses

Fresh cross-sectional heart tissue slices and liver tissue slices were fixed in 10% neutral buffered formalin, dehydrated through graded alcohols, cleared in xylene and embedded in paraffin wax. Tissue blocks were sectioned into 4-5 μm thick slices using a microtome (Leica Biosystems, Germany). The tissue section slides were then deparaffinized in xylene, rehydrated through graded alcohols to water, and stained with Hematoxylin & Eosin (H&E).

For cardiac fibrosis evaluation, the rehydrated heart tissue slides were stained sequentially with Weigert’s iron Hematoxylin, Biebrich Scarlet-Acid Fuchsin, and Aniline Blue (Modified Masson’s Trichrome Stain Kit, Solarbio, China). Slides were then dehydrated, cleared again, and mounted with a resinous medium. Images were capture under a high-resolution light microscope (Nikon, Japan).

### High-fat diet treatment

8-week-old *Fgf21*^*-/-*^ and WT mice were fed a high-fat diet (60 kcal% fat, Research Diets, USA) for only 12 or 15 weeks. Metabolic and heart function experiments were then conducted. The metabolically healthy stage of chronic high-fat diet intake was determined by the lack of changes in serum AST marker levels using a Micro Glutamic-oxalacetic Transaminase (GOT) Assay Kit (Solarbio, China).

### Cumulative body weight

*Fgf21*^*-/-*^ and WT mice were fed a chow diet or a high-fat diet continuously *ad libitum*. The body weights of individual mice were recorded at weekly interval. At week 12, serum AST levels was measured. Mice without AST changes were used for metabolic, heart rate and contractility analyses.

### Glucose tolerance test (IPGTT)

Glucose tolerance test was carried out in 8-week-old mice following a 12-h fast and standard procedures. After measuring fasted blood glucose levels, mice were injected intraperitoneally with glucose (2 mg/g body weight, 20% glucose solution; Sigma-Aldrich). Blood glucose levels were determined at 15, 30, 60, and 90 min after glucose injection using Glucometer. Area-under-curve value of glucose excursion curve for each mouse was calculated in GraphPad 9.

### Recombinant human FGF21

A cDNA fragment encoding human FGF21 (Ala28-Leu209) was subcloned into the bacterial expression vector pET-3c. After expression in the *E. coli* BL21 DE3 strain, recombinant FGF21 was purified from inclusion body by sequential chromatograph with DEAE Sepharose, Phenyl Sepharose-6, and Sephadex G-25 desalting columns. The protein fraction was lyophilized by vacuum at −80 °C for storage. Before used for animal study, the protein powder was dissolved in 1 x PBS, and its biological activity was assessed in a HEK293 cell line engineered to ectopically express betaKlotho.

### Acute rhFGF21 treatment

Pure and active rhFGF21 was injected (*i.p*. or *i.v*. where indicated) into mice at a concentration of 1 mg/kg body weight in a 100 μl buffered saline solution into experimental mice. For experiments involving both 12-h and 24-h fasts, mice were injected at 8:00 AM immediately following the 12-h or 24-h fast. Mice were experimented or euthanized 2 h after the injection.

### RNA isolation and quantitative RT-PCR

Total RNA was isolated from mouse heart, eWAT, iWAT or liver using RNAiso Plus (TaKaRa, Japan) including DNase treatment. Total RNA (1 μg) was retrotranscribed with HiScript III 1st Strand cDNA Synthesis kit (Vazyme, China) in the presence of random primers following the manufacturer’s instruction. Two microliters of a 1/10 dilution of the 1st strand cDNA mixture were used in each PCR reaction in a total reaction volume of 10 μl using ChamQ Universal SYBR qPCR Master Mix (Vazyme, China). The qPCR amplification scheme was one cycle of 95 °C for 5 min followed by 40 cycles of 95 °C for 10 seconds and 60 °C for 30 seconds, performed in a CFX96 Touch™ Real-Time PCR Detection System (Bio-Rad Laboratories, Inc., Hercules, USA). The qPCR primers were listed in Table S5. After confirming the specificity of the PCR products with melting curve, the comparative cycle threshold method (2^-ddCT^) was used for calculating the relative expression levels of interest genes and normalized to the expression level of 18S, *Rplp0* or *Hprt1*.

### Immunoblot

Heart, liver and WAT samples were isolated freshly and frozen at −80 °C. Protein lysates were extracted in cold RIPA lysis buffer. 20–25 μg of total cellular protein for each sample were separated by standard SDS-PAGE and transferred onto PVDF membranes. Proteins were detected with the indicated antibodies listed in the Antibody table, and blots were imaged using Amersham Imager 680 (GE HealthCare, USA). ImageJ was used for densitometric protein quantification.

### Active kinase levels

Cardiac kinase activation for LKB1, AMPK, and mTOR were assessed on Western Blotting with specific antibodies for pLKB1(S431), pAMPK(T172), pmTOR(S2448), and their respective total proteins. The total relative active protein level for each kinase was determined as: total kinase activity = (p-Kinase/Kinase) x (Kinase/internal standard).

### Quantification and statistical analysis

For experiments with two groups, the two-tailed unpaired Student’s *t* test was used for statistical comparison. Ordinary one-way ANOVA with Tukey’s HSD post-hoc test, as indicated in the figure legends, was used for the comparisons within multiple groups. Data are expressed as the means ± standard errors of the means (SEM) unless otherwise stated. A *p* value of < 0.05 was considered statistically significant: ^*^p < 0.05, ^**^p < 0.01, ^***^p < 0.001, ^****^p < 0.0001. For clarity, different types of symbols are used to distinguish test groups where indicated: * for comparisons between the *Fgf21*^*-/-*^ and WT groups by Student’s t test or one-way ANOVA, ^$^ for comparisons between treatment groups in WT mice, and ^#^ for comparisons between treatment groups in *Fgf21*^*-/-*^ mice, unless otherwise specified. Comparisons without a *p* value or the above symbols are not statistically significant. For RNA-Seq, *p* values were calculated using the Wald test with Benjamini-Hochberg correction. Statistical analysis and data visualization were conducted using Microsoft Excel, GraphPad Prism 9 (GraphPad Software, USA), Instant Clue, and R (v4.4.2).

## Data and resource availability

### Lead Contact

Further information and requests for resources and reagents should be directed to and will be fulfilled by the lead contact, Yongde Luo (yongdeluo08@wmu.edu.cn).

## Supplementary information

Supplementary Materials

Data S1

Supplementary Table S1

Supplementary Table S2

Supplementary Table S3

## Data Availability

Cardiac RNA-Seq data have been deposited in the Sequence Read Archive (SRA) under the access number PRJNA1189445 and are presented in Table S3. Untargeted serum metabolomic data and targeted cardiac energy metabolomic data have been deposited to MetaboLights (MTBLS11725, MTBLS11726) and are presented in Tables S1 and S2. Source data and uncropped blots are provided in Data S1 in supplementary materials. No original code was generated in this study. Any additional information required to reanalyze the data reported in this paper is available from the lead contact upon request.

## Materials availability

The mouse lines generated in this study are available from the lead contact upon request.

## Code availability

Cardiac RNA-Seq data have been deposited in the Sequence Read Archive (SRA) under the access number PRJNA1189445 and are presented in Table S3. Untargeted serum metabolomic data and targeted cardiac energy metabolomic data have been deposited to MetaboLights (MTBLS11725, MTBLS11726) and are presented in Tables S1 and S2. Source data and uncropped blots are provided in Data S1 in supplementary materials. No original code was generated in this study. Any additional information required to reanalyze the data reported in this paper is available from the lead contact upon request.

## References

[1] Lopaschuk, G. D., Karwi, Q. G., Tian, R., Wende, A. R. & Abel, E. D. Cardiac energy metabolism in heart failure. *Circ. Res.* **128**, 1487–1513 (2021). 33983836 10.1161/CIRCRESAHA.121.318241 PMC8136750

[2] Ritterhoff, J. & Tian, R. Metabolism in cardiomyopathy: every substrate matters. *Cardiovasc. Res.* **113**, 411–421 (2017). 28395011 10.1093/cvr/cvx017 PMC5852620

[3] Aubert, G. et al. The failing heart relies on ketone bodies as a fuel. *Circulation* **133**, 698–705 (2016). 26819376 10.1161/CIRCULATIONAHA.115.017355 PMC4766035

[4] Carley, A. N. et al. Short-chain fatty acids outpace ketone oxidation in the failing heart. *Circulation* **143**, 1797–1808 (2021). 33601938 10.1161/CIRCULATIONAHA.120.052671 PMC8096711

[5] Badman, M. K. et al. Hepatic fibroblast growth factor 21 is regulated by PPARalpha and is a key mediator of hepatic lipid metabolism in ketotic states. *Cell Metab.* **5**, 426–437 (2007). 17550778 10.1016/j.cmet.2007.05.002

[6] Inagaki, T. et al. Endocrine regulation of the fasting response by PPARalpha-mediated induction of fibroblast growth factor 21. *Cell Metab.* **5**, 415–425 (2007). 17550777 10.1016/j.cmet.2007.05.003

[7] Forsstrom, S. et al. Fibroblast growth factor 21 drives dynamics of local and systemic stress responses in mitochondrial myopathy with mtDNA deletions. *Cell Metab.* **30**, 1040–1054 e1047 (2019). 31523008 10.1016/j.cmet.2019.08.019

[8] Restelli, L. M. et al. Neuronal mitochondrial dysfunction activates the integrated stress response to induce fibroblast growth factor 21. *Cell Rep.* **24**, 1407–1414 (2018). 30089252 10.1016/j.celrep.2018.07.023 PMC6092266

[9] Schlein, C. et al. FGF21 lowers plasma triglycerides by accelerating lipoprotein catabolism in white and brown adipose tissues. *Cell Metab.* **23**, 441–453 (2016). 26853749 10.1016/j.cmet.2016.01.006

[10] Khan, N. A. et al. mTORC1 regulates mitochondrial integrated stress response and mitochondrial myopathy progression. *Cell Metab.* **26**, 419–428 e415 (2017). 28768179 10.1016/j.cmet.2017.07.007

[11] Lehtonen, J. M. et al. FGF21 is a biomarker for mitochondrial translation and mtDNA maintenance disorders. *Neurology* **87**, 2290–2299 (2016). 27794108 10.1212/WNL.0000000000003374 PMC5270510

[12] Suomalainen, A. et al. FGF-21 as a biomarker for muscle-manifesting mitochondrial respiratory chain deficiencies: a diagnostic study. *Lancet Neurol.* **10**, 806–818 (2011). 21820356 10.1016/S1474-4422(11)70155-7 PMC7568343

[13] Kharitonenkov, A. et al. FGF-21 as a novel metabolic regulator. *J. Clin. Invest.* **115**, 1627–1635 (2005). 15902306 10.1172/JCI23606 PMC1088017

[14] Chou, R. H. et al. Circulating fibroblast growth factor 21 is associated with diastolic dysfunction in heart failure patients with preserved ejection fraction. *Sci. Rep.* **6**, 33953 (2016). 27650781 10.1038/srep33953 PMC5030655

[15] Shen, Y. et al. Contribution of serum FGF21 level to the identification of left ventricular systolic dysfunction and cardiac death. *Cardiovasc. Diabetol.* **16**, 106 (2017). 28821258 10.1186/s12933-017-0588-5 PMC5562996

[16] Li, Q. et al. Association between serum fibroblast growth factor 21 and mortality among patients with coronary artery disease. *J. Clin. Endocrinol. Metab.* **101**, 4886–4894 (2016). 27662438 10.1210/jc.2016-2308

[17] Fan, L., Gu, L., Yao, Y. & Ma, G. Elevated serum fibroblast growth factor 21 is relevant to heart failure patients with reduced ejection fraction. *Comput. Math. Methods Med.* **2022**, 7138776 (2022). 35069790 10.1155/2022/7138776 PMC8767358

[18] Frayling, T. M. et al. A common allele in FGF21 associated with sugar intake is associated with body shape, lower total body-fat percentage, and higher blood pressure. *Cell Rep.* **23**, 327–336 (2018). 29641994 10.1016/j.celrep.2018.03.070 PMC5912948

[19] Giontella, A. et al. Renoprotective effects of genetically proxied fibroblast growth factor 21: Mendelian randomization, proteome-wide and metabolome-wide association study. *Metabolism* **145**, 155616 (2023). 37302695 10.1016/j.metabol.2023.155616

[20] Larsson, S. C. & Gill, D. Genetic evidence supporting fibroblast growth factor 21 signalling as a pharmacological target for cardiometabolic outcomes and Alzheimer’s disease. *Nutrients* **13**, 1504 (2021). 10.3390/nu13051504 PMC8146158 33946944

[21] Gaich, G. et al. The effects of LY2405319, an FGF21 analog, in obese human subjects with type 2 diabetes. *Cell Metab.* **18**, 333–340 (2013). 24011069 10.1016/j.cmet.2013.08.005

[22] Harrison, S. A. et al. Efruxifermin in non-alcoholic steatohepatitis: a randomized, double-blind, placebo-controlled, phase 2a trial. *Nat. Med.* **27**, 1262–1271 (2021). 34239138 10.1038/s41591-021-01425-3

[23] Sanyal, A. et al. Pegbelfermin (BMS-986036), a PEGylated fibroblast growth factor 21 analogue, in patients with non-alcoholic steatohepatitis: a randomised, double-blind, placebo-controlled, phase 2a trial. *Lancet* **392**, 2705–2717 (2019). 30554783 10.1016/S0140-6736(18)31785-9

[24] Planavila, A. et al. Fibroblast growth factor 21 protects against cardiac hypertrophy in mice. *Nat. Commun.* **4**, 2019 (2013). 23771152 10.1038/ncomms3019

[25] Planavila, A. et al. Fibroblast growth factor 21 protects the heart from oxidative stress. *Cardiovasc. Res.* **106**, 19–31 (2015). 25538153 10.1093/cvr/cvu263

[26] Brahma, M. K. et al. Fibroblast growth factor 21 is induced upon cardiac stress and alters cardiac lipid homeostasis. *J. Lipid Res.* **55**, 2229–2241 (2014). 25176985 10.1194/jlr.M044784 PMC4617126

[27] Ferrer-Curriu, G. et al. Fibroblast growth factor-21 protects against fibrosis in hypertensive heart disease. *J. Pathol.* **248**, 30–40 (2019). 30582148 10.1002/path.5226

[28] Badman, M. K., Koester, A., Flier, J. S., Kharitonenkov, A. & Maratos-Flier, E. Fibroblast growth factor 21-deficient mice demonstrate impaired adaptation to ketosis. *Endocrinology* **150**, 4931–4940 (2009). 19819944 10.1210/en.2009-0532 PMC2775979

[29] Hotta, Y. et al. Fibroblast growth factor 21 regulates lipolysis in white adipose tissue but is not required for ketogenesis and triglyceride clearance in liver. *Endocrinology* **150**, 4625–4633 (2009). 19589869 10.1210/en.2009-0119

[30] Potthoff, M. J. et al. FGF21 induces PGC-1alpha and regulates carbohydrate and fatty acid metabolism during the adaptive starvation response. *Proc. Natl. Acad. Sci. USA* **106**, 10853–10858 (2009). 19541642 10.1073/pnas.0904187106 PMC2705613

[31] Elizondo, G., Matern, D., Vockley, J., Harding, C. O. & Gillingham, M. B. Effects of fasting, feeding and exercise on plasma acylcarnitines among subjects with CPT2D, VLCADD and LCHADD/TFPD. *Mol. Genet. Metab.* **131**, 90–97 (2020). 32928639 10.1016/j.ymgme.2020.09.001 PMC8048763

[32] Rubio-Gozalbo, M. E., Bakker, J. A., Waterham, H. R. & Wanders, R. J. Carnitine-acylcarnitine translocase deficiency, clinical, biochemical and genetic aspects. *Mol. Asp. Med.* **25**, 521–532 (2004). 10.1016/j.mam.2004.06.007 15363639

[33] Strauss, A. W. et al. Molecular basis of human mitochondrial very-long-chain acyl-CoA dehydrogenase deficiency causing cardiomyopathy and sudden death in childhood. *Proc. Natl Acad. Sci. USA* **92**, 10496–10500 (1995). 7479827 10.1073/pnas.92.23.10496 PMC40638

[34] Lee, R. G. et al. Quantitative subcellular reconstruction reveals a lipid mediated inter-organelle biogenesis network. *Nat. Cell Biol.* **26**, 57–71 (2024). 38129691 10.1038/s41556-023-01297-4

[35] Murari, A. et al. Phospholipids can regulate complex I assembly independent of their role in maintaining mitochondrial membrane integrity. *Cell Rep.* **42**, 112846 (2023). 37516961 10.1016/j.celrep.2023.112846 PMC10718285

[36] Sanchez-Lopez, E. et al. Choline uptake and metabolism modulate macrophage IL-1beta and IL-18 production. *Cell Metab.* **29**, 1350–1362 e1357 (2019). 30982734 10.1016/j.cmet.2019.03.011 PMC6675591

[37] Brackett, J. C. et al. Two alpha subunit donor splice site mutations cause human trifunctional protein deficiency. *J. Clin. Invest.* **95**, 2076–2082 (1995). 7738175 10.1172/JCI117894 PMC295799

[38] Al-Habsi, M. et al. Spermidine activates mitochondrial trifunctional protein and improves antitumor immunity in mice. *Science* **378**, eabj3510 (2022). 36302005 10.1126/science.abj3510

[39] Eisenberg, T. et al. Cardioprotection and lifespan extension by the natural polyamine spermidine. *Nat. Med.* **22**, 1428–1438 (2016). 27841876 10.1038/nm.4222 PMC5806691

[40] Nayak, A. et al. N8-acetylspermidine: a polyamine biomarker in ischemic cardiomyopathy with reduced ejection fraction. *J. Am. Heart Assoc.* **9**, e016055 (2020). 32458724 10.1161/JAHA.120.016055 PMC7429012

[41] Puleston, D. J. et al. Polyamines and eIF5A hypusination modulate mitochondrial respiration and macrophage activation. *Cell Metab.* **30**, 352–363 e358 (2019). 31130465 10.1016/j.cmet.2019.05.003 PMC6688828

[42] Leiria, L. O. et al. 12-lipoxygenase regulates cold adaptation and glucose metabolism by producing the omega-3 Lipid 12-HEPE from brown fat. *Cell Metab.* **30**, 768–783 e767 (2019). 31353262 10.1016/j.cmet.2019.07.001 PMC6774888

[43] Schulze, M. B., Minihane, A. M., Saleh, R. N. M. & Riserus, U. Intake and metabolism of omega-3 and omega-6 polyunsaturated fatty acids: nutritional implications for cardiometabolic diseases. *Lancet Diabetes Endocrinol.* **8**, 915–930 (2020). 32949497 10.1016/S2213-8587(20)30148-0

[44] Takayama, K. et al. Thromboxane A2 and prostaglandin F2alpha mediate inflammatory tachycardia. *Nat. Med.* **11**, 562–566 (2005). 15834430 10.1038/nm1231

[45] Karmazyn, M., Tani, M. & Neely, J. R. Effect of prostaglandins I2 (prostacyclin) and F2 alpha on function, energy metabolism, and calcium uptake in ischaemic/reperfused hearts. *Cardiovasc. Res.* **27**, 396–402 (1993). 8490938 10.1093/cvr/27.3.396

[46] Davids, M., Ndika, J. D., Salomons, G. S., Blom, H. J. & Teerlink, T. Promiscuous activity of arginine:glycine amidinotransferase is responsible for the synthesis of the novel cardiovascular risk factor homoarginine. *FEBS Lett.* **586**, 3653–3657 (2012). 23010440 10.1016/j.febslet.2012.08.020

[47] Mahemuti, N. et al. Urinary albumin-to-creatinine ratio in normal range, cardiovascular health, and all-cause mortality. *JAMA Netw. Open* **6**, e2348333 (2023). 38113044 10.1001/jamanetworkopen.2023.48333 PMC10731498

[48] Deng, Y. et al. An adipo-biliary-uridine axis that regulates energy homeostasis. *Science* **355**, 1173–1182 (2017). 10.1126/science.aaf5375 PMC5832364 28302796

[49] Kirino, Y. et al. Codon-specific translational defect caused by a wobble modification deficiency in mutant tRNA from a human mitochondrial disease. *Proc. Natl. Acad. Sci. USA* **101**, 15070–15075 (2004). 15477592 10.1073/pnas.0405173101 PMC524061

[50] Chuang, D. T., Ku, L. S. & Cox, R. P. Thiamin-responsive maple-syrup-urine disease: decreased affinity of the mutant branched-chain alpha-keto acid dehydrogenase for alpha-ketoisovalerate and thiamin pyrophosphate. *Proc. Natl. Acad. Sci. USA* **79**, 3300–3304 (1982). 6954481 10.1073/pnas.79.10.3300 PMC346403

[51] Komine, S. et al. Taurine supplementation enhances endurance capacity by delaying blood glucose decline during prolonged exercise in rats. *Amino Acids* **54**, 251–260 (2022). 35122528 10.1007/s00726-021-03110-8 PMC8894168

[52] Wei, W. et al. PTER is a N-acetyltaurine hydrolase that regulates feeding and obesity. *Nature* **633**, 182–188 (2024). 39112712 10.1038/s41586-024-07801-6 PMC11374699

[53] DiNicolantonio, J. J., Liu, J. & O’Keefe, J. H. Thiamine and Cardiovascular Disease: A Literature Review. *Prog. Cardiovasc. Dis.* **61**, 27–32 (2018). 29360523 10.1016/j.pcad.2018.01.009

[54] Ito, T. et al. Taurine depletion caused by knocking out the taurine transporter gene leads to cardiomyopathy with cardiac atrophy. *J. Mol. Cell. Cardiol.* **44**, 927–937 (2008). 18407290 10.1016/j.yjmcc.2008.03.001

[55] Pion, P. D., Kittleson, M. D., Rogers, Q. R. & Morris, J. G. Myocardial failure in cats associated with low plasma taurine: a reversible cardiomyopathy. *Science* **237**, 764–768 (1987). 3616607 10.1126/science.3616607

[56] Lewis, G. D. et al. Metabolic signatures of exercise in human plasma. *Sci. Transl. Med.* **2**, 33ra37 (2010). 20505214 10.1126/scitranslmed.3001006 PMC3010398

[57] Niemann, B. et al. Apoptotic brown adipocytes enhance energy expenditure via extracellular inosine. *Nature* **609**, 361–368 (2022). 35790189 10.1038/s41586-022-05041-0 PMC9452294

[58] Sohn, J. H. et al. Liver mitochondrial cristae organizing protein MIC19 promotes energy expenditure and pedestrian locomotion by altering nucleotide metabolism. *Cell Metab.* **35**, 1356–1372 e1355 (2023). 37473754 10.1016/j.cmet.2023.06.015 PMC10528355

[59] Tong, L. et al. Transketolase promotes MAFLD by limiting inosine-induced mitochondrial activity. *Cell Metab*. **36**, 1013–1029 (2024). 10.1016/j.cmet.2024.03.003 38547864

[60] Ikeda, Y. et al. Cardiac-specific deletion of LKB1 leads to hypertrophy and dysfunction. *J. Biol. Chem.* **284**, 35839–35849 (2009). 19828446 10.1074/jbc.M109.057273 PMC2791013

[61] Kingma, J. G. Jr, Simard, D. & Rouleau, J. R. Timely administration of AICA riboside reduces reperfusion injury in rabbits. *Cardiovasc. Res.* **28**, 1003–1007 (1994). 7954584 10.1093/cvr/28.7.1003

[62] Sahu, U. et al. Pyrimidines maintain mitochondrial pyruvate oxidation to support de novo lipogenesis. *Science* **383**, 1484–1492 (2024). 38547260 10.1126/science.adh2771 PMC11325697

[63] Hondares, E. et al. Hepatic FGF21 expression is induced at birth via PPARalpha in response to milk intake and contributes to thermogenic activation of neonatal brown fat. *Cell Metab.* **11**, 206–212 (2010). 20197053 10.1016/j.cmet.2010.02.001 PMC2847690

[64] Icard, P., Alifano, M., Donnadieu, E. & Simula, L. Fructose-1,6-bisphosphate promotes PI3K and glycolysis in T cells?. *Trends Endocrinol. Metab.* **32**, 540–543 (2021). 34016523 10.1016/j.tem.2021.04.013

[65] Sullivan, A. I. et al. Characterization of FGF21 Sites of Production and Signaling in Mice. *Endocrinology* **165**, bqae120 (2024). 10.1210/endocr/bqae120 39253796

[66] Mastaglia, F. L., Thompson, P. L. & Papadimitriou, J. M. Mitochondrial myopathy with cardiomyopathy, lactic acidosis and response to prednisone and thiamine. *Aust. N. Z. J. Med.* **10**, 660–664 (1980). 6938188 10.1111/j.1445-5994.1980.tb04250.x

[67] Haemmerle, G. et al. ATGL-mediated fat catabolism regulates cardiac mitochondrial function via PPAR-alpha and PGC-1. *Nat. Med.* **17**, 1076–1085 (2011). 21857651 10.1038/nm.2439 PMC3244833

[68] Opie, L. H. & Owen, P. Effects of increased mechanical work by isolated perfused rat heart during production or uptake of ketone bodies. Assessment of mitochondrial oxidized to reduced free nicotinamide-adenine dinucleotide ratios and oxaloacetate concentrations. *Biochem. J.* **148**, 403–415 (1975). 173281 10.1042/bj1480403 PMC1165557

[69] Ye, M. et al. FGF21-FGFR1 coordinates phospholipid homeostasis, lipid droplet function, and ER stress in obesity. *Endocrinology* **157**, 4754–4769 (2016). 27690692 10.1210/en.2016-1710

[70] Adams, A. C. et al. The breadth of FGF21 metabolic actions are governed by FGFR1 in adipose tissue. *Mol. Metab.* **2**, 31–37 (2012). 24024127 10.1016/j.molmet.2012.08.007 PMC3757657

[71] Zhang, Y. et al. The starvation hormone, fibroblast growth factor-21, extends lifespan in mice. *Elife* **1**, e00065 (2012). 23066506 10.7554/eLife.00065 PMC3466591

[72] Cefalo, C. M. A. et al. Relationship between hemoglobin glycation index and myocardial mechano-energetic efficiency in non-diabetic individual. *Cardiovasc. Diabetol.* **24**, 148 (2025). 40176082 10.1186/s12933-025-02710-y PMC11966833

