# Supplementary material for: A two-strata energy flux system driven by a stress hormone prioritizes cardiac energetics
Source: Signal Transduct Target Ther. 2025 Sep 26;10:315. doi: 10.1038/s41392-025-02402-9 (PMC12464335; doi:10.1038/s41392-025-02402-9)
Supplement: Supplementary file 1 — Supplementary Materials [file 41392_2025_2402_MOESM1_ESM.docx]

Supplementary Materials for

A two-strata energy flux system driven by a stress hormone prioritizes cardiac energetics

Zhiheng Rao^1,2,☨^, Zhichao Chen^1,☨^, Yuxuan Bao^1,☨^, Zhenzhen Lu^1,☨^, Yuli Tang^1^, Jiamei Zhu^1^, Jianjia Ma^1^, Siyang Dong^1^, Jiawei Shi^1^, Suhui Sheng^1^, Yajing Chen^1^, Jiaojiao Wang^1^, Alan Vengai Mukondiwa^1^, Ziyue Li^1^, Xulan Wang^1^, Zibo Huang^1^, Chi Li^1^, Wumengwei Ding^1^, Mengjie Chen^1^, Ziyi Han^1^, Cong Wang^1^, Xuebo Pang^1^, Xiaojie Wang^1,3^, Hong Zhu^4^, Li Lin^1,3^, Zhifeng Huang^1,3^, Weiqin Lu^5^, Xiaokun Li^1,3,^*, Yongde Luo^1,3,4,6,^*

*Correspondence to: yongdeluo08@wmu.edu.cn (Y.L.), xiaokunli@wmu.edu.cn (X.L.)

**This file includes:**

Figures S1 to S30

Tables S1 to S3 legends

Tables S4 to S5

Key resource table

**Figure S1.**

**
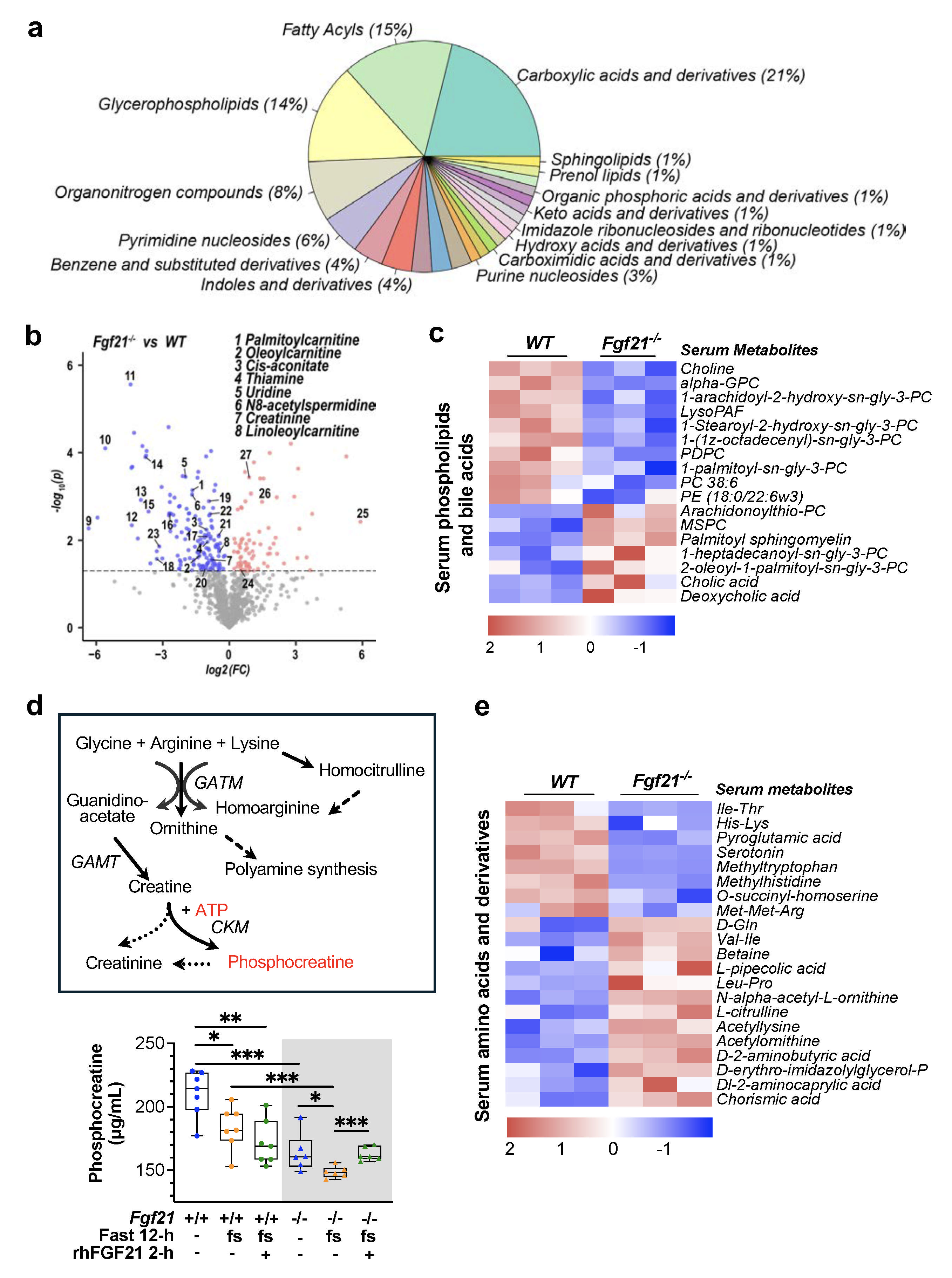
**

**Fig. S1. Effects of germline *Fgf21* deletion on serum metabolites in mice.**

**Related to Fig. 1a-1c.**

(**a**) Pie chart indicating class proportions of altered serum metabolites due to FGF21 deficiency vs WT (n=3 per group) under normal conditions.

(**b**) Volcano plot indicating altered serum metabolites with a cutoff value of *p* < 0.05. Some representative metabolites are shown: 1, L-palmitoylcarnitine; 2, L-oleoylcarnitine; 3, cis-aconitate; 4, Thiamine; 5. Uridine; 6, N8-acetylspermidine; 7, creatinine; 8, L-linoleoylcarnitine; 9, 14-hydroxy-4z,7z,10z,12e,16z,19z-docosahexaenoic acid (14-HDHA, C22:6); 10, 5-methoxytryptophan; 11, 12-oxo-5z,8z,10e,14z-eicosatetraenoic acid (12-oxo-ETE); 12, 12s-hydroxy-5z,8z,10e,14z-eicosatetraenoic acid (12(s)-HETE, C20:4); 13, S-D-lactoylglutathione; 14, L-homocitrulline; 15, serotonin; 16, 1-methylhistamine; 17, Citraconic acid; 18, Ile-thr; 19, taurine; 20, AICAR; 21, palmitic acid; 22, choline; 23, adenosine; 24, inosine; 25, 9-deoxy-9-methyleneprostaglandin e2; 26, Val-Ile; 27, L-homoarginine.

(**c**) Heatmap depicting changes in choline, phospholipids and basic bile acid, especially phosphatidylcholines due to FGF21 deficiency. Increases in both basic bile acids may indicate (1) conservation of resources for essential functions, such as energy provision, or (2) less efficient fat digestion and malabsorption of fats and fat-soluble vitamins.

(**d**) Upper: Diagram of the pathways for biosynthesis of creatine and creatinine from arginine, involving homocitrulline and homoarginine in association with polyamine. The image is generated in PowerPoint. Lower: Changes in serum phosphocreatine levels in whole-body FGF21-deficient mice under the indicated conditions compared to wildtype mice.

(**e**) Heatmap depicting changes in serum amino acids and derivatives, such as glutamine, betaine, serotonin, L-citrulline, Val-Ile, L-pipecolic acid, and pyroglutamic acid (a glutathione derivative), in addition to arginine and its derivatives (Fig. 1b).

**Figure S2.**

**
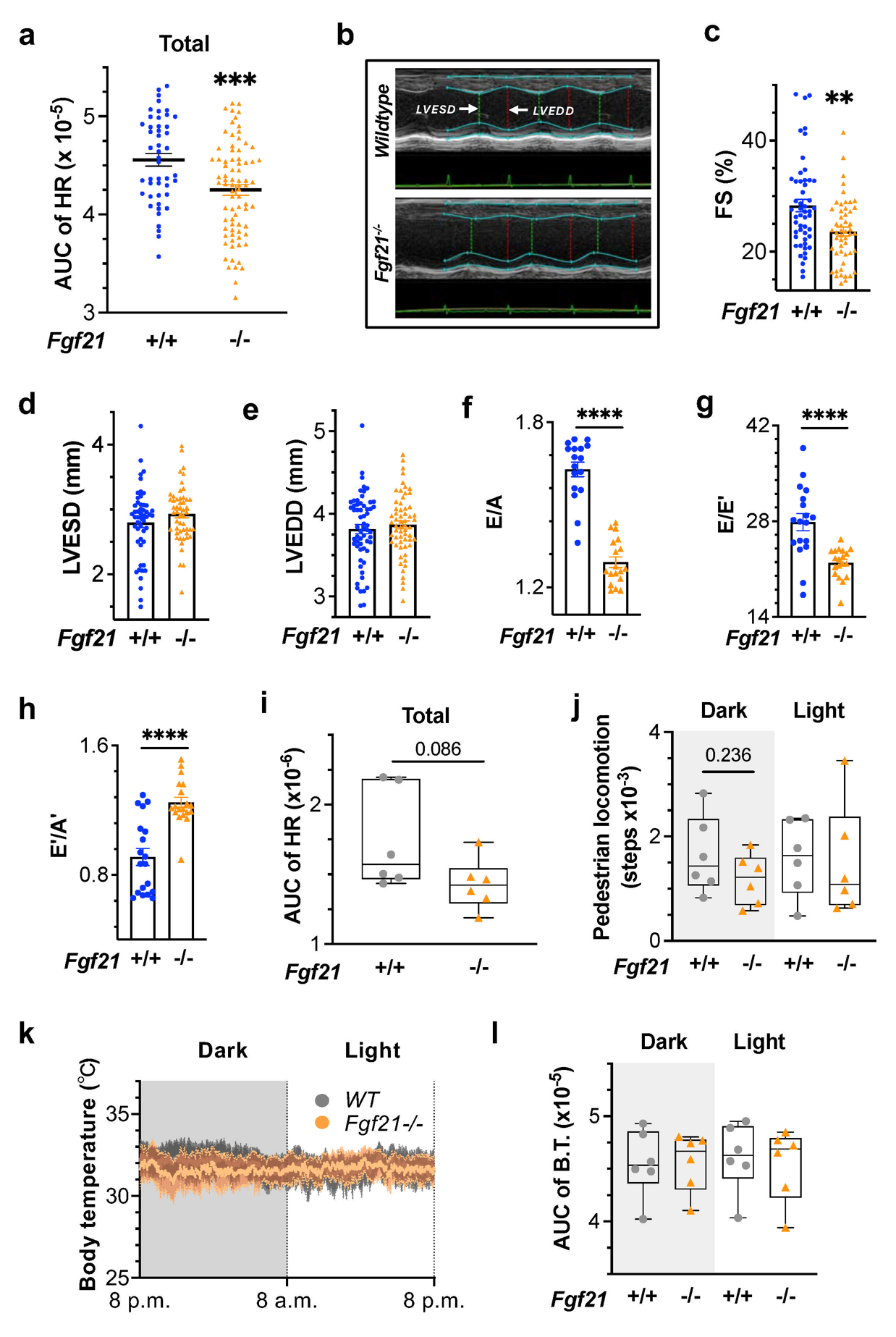
**

**Fig. S2. Effects of germline *Fgf21* deletion on cardiac Echo parameters, telemetric heartrate and physical parameters.**

**Related to Figures 1d-1j.**

(**a**) Changes in total AUC of heart rate (HR) in FGF21-deficient (n=78) vs WT (n=47) mice, from electrocardiogram.

(**b**) Representative echocardiogram measurements of LVESD and LVEDD in the indicated mice.

(**c**-**h**) Changes in the Echo parameters as indicated. For FS, LVESD, and LVEDD, n=51 for both FGF21-deficient and WT mice. For E/A, E/E’, and E’/A’, n=17-19 per group.

(**i**) Changes in total AUC of HR in FGF21-deficient (n=6) vs WT (n=6) mice, from telemetry under normal conditions.

(**j**) Changes in ambulatory movements in FGF21-deficient vs WT mice.

(**k**) Changes in body temperature in FGF21-deficient vs WT mice.

(**l**) Changes in total AUC of body temperature (B.T.) as in k.

**Figure S3.**

**
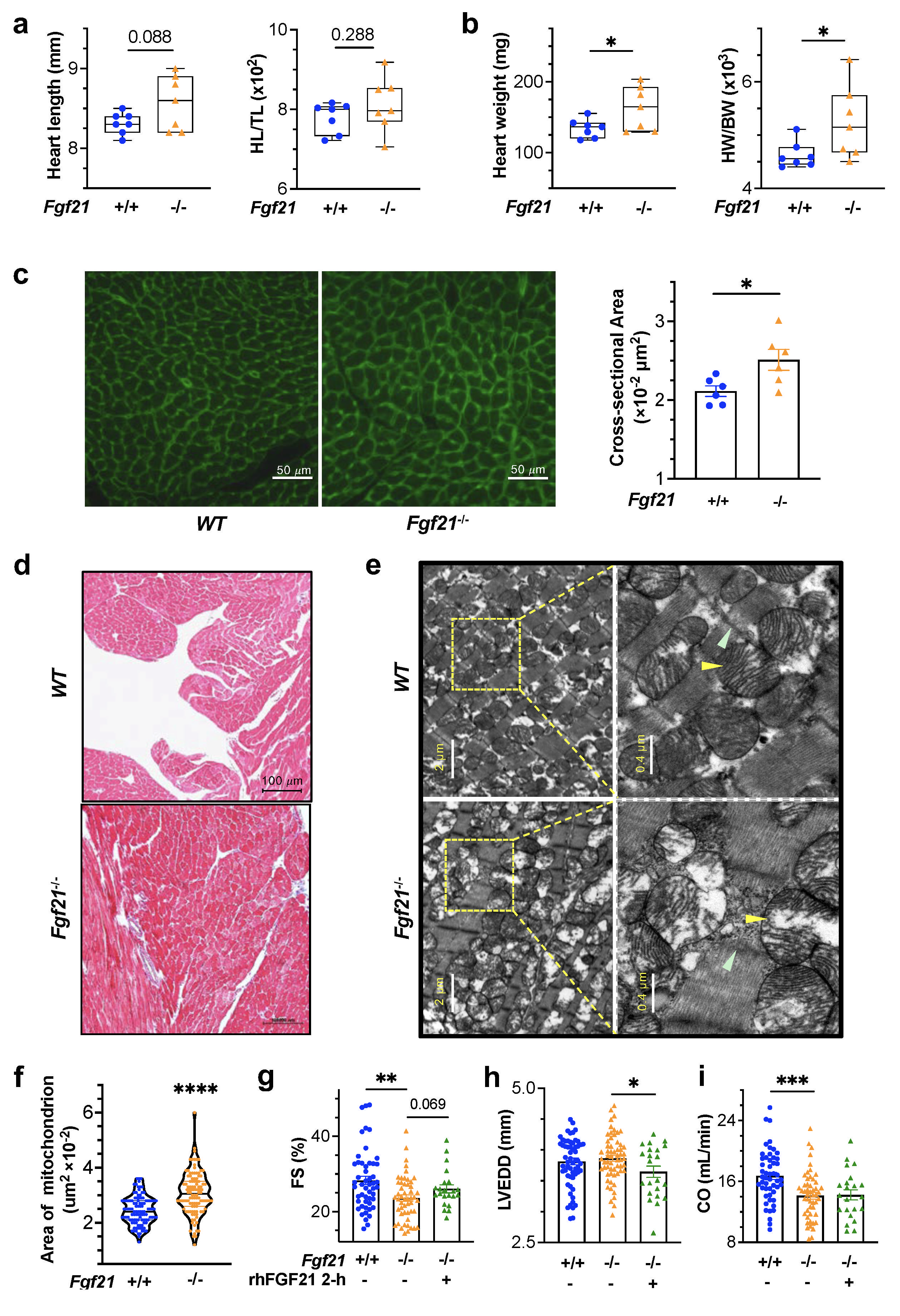
**

**Fig. S3. Effects of germline *Fgf21* deletion on heart morphology and tissue ultrastructure.**

**Related to Fig. 1k-1q.**

(**a**) Changes in absolute heart length (HL) and relative HL compared to tibia length (TL) in FGF21-deficient (n=7) vs WT (n=7) mice under normal conditions.

(**b**) Changes in absolute heart weight (HW) and relative HW compared to body weight (BW).

(**c**) Representative images for mildly dilated cardiac myofibers analyzed by Wheat Germ Agglutinin (WGA) fluorescent staining on left ventricle cross-sections, due to FGF21 deficiency. Right, statistical analysis of cross-sectional areas of myofibers (n=6 for each group).

(**d**) Representative images for cardiac fibrosis analyzed by Masson’s Trichome stain on left ventricle cross-sections (n=6 for each group).

(**e**) Representative images for low-magnification and high magnification views of cardiac myofiber ultrastructure and interfibrillar mitochondria (n=6 for each group).

(**f**) Changes in cardiac mitochondria sizes in FGF21-deficient vs WT mice under normal conditions. n=108 per group.

(**g**-**i**) Effects of acute rhFGF21 treatment (1 mg/kilogram body weight, *i.p.*) on FS, LVEDD, and CO in FGF21-deficient mice under normal conditions.

**Figure S4.**

**
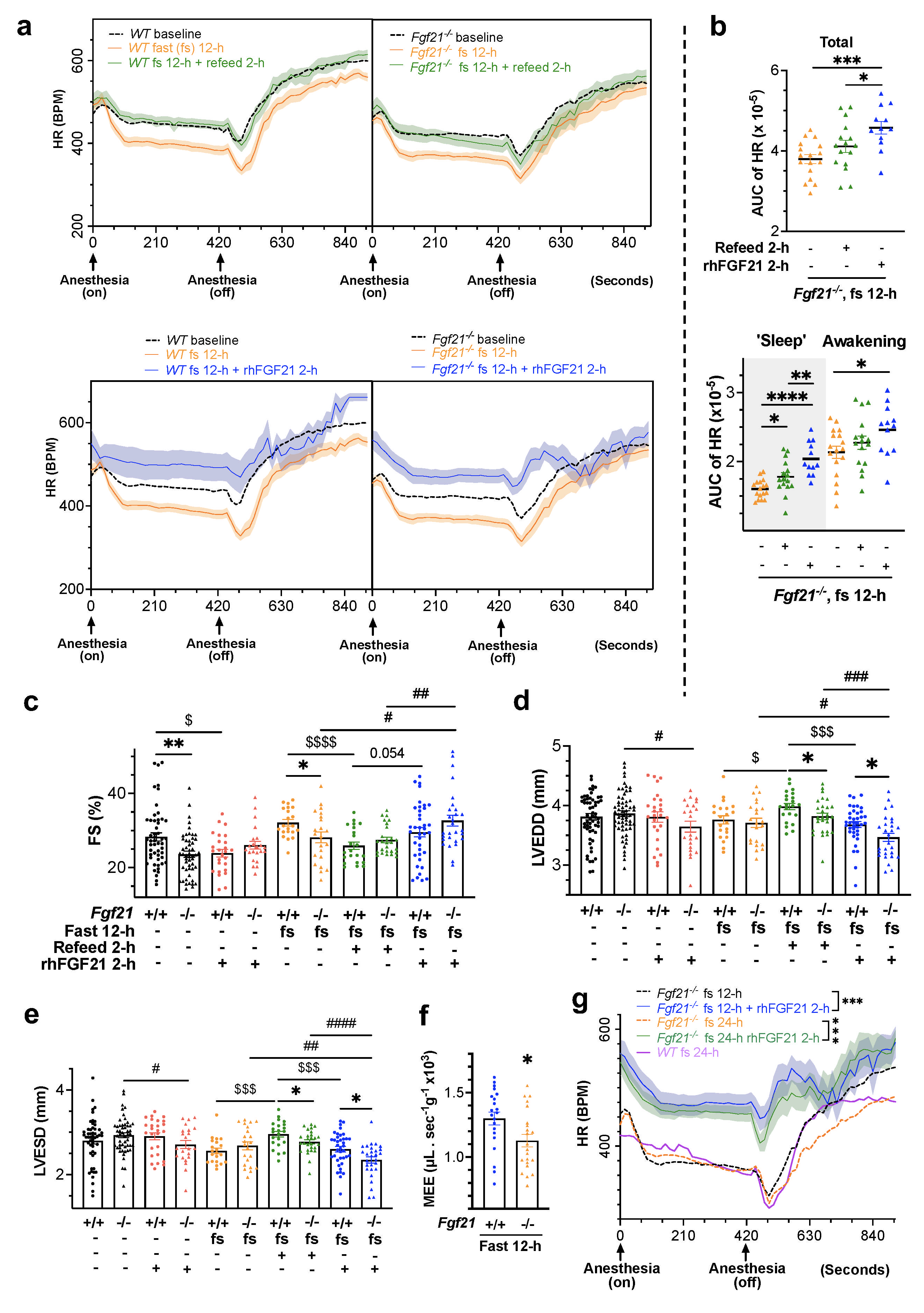
**

**Fig. S4. Effects of fast, refeeding and FGF21 restoration on heart rate and contractility in *Fgf21*-null mice.**

**Related to Fig. 2a-2f.**

(a) More detailed comparisons for the changes in HR in FGF21-deficient vs WT mice (n=12-17) fasted for 12 hours (h), followed by 2-h rhFGF21 treatment (while still on fast) (lower panel) compared to 2-h refeeding (upper panel). See Fig. 2b-2c.

(b) Total AUC values (upper) and both ‘sleep’ and awakening phase AUC values (lower) in *Fgf21*-null mice under conditions as indicated. ‘Sleep’, an immobile state under anesthetic inhalation; awakening phase, a recovery state after anesthetic removal.

(c-e) Changes in Echo parameters FS, LVEDD and LVESD in *Fgf21*-null vs WT mice under conditions as indicated.

(f) Changes in MEE in *Fgf21*-null vs WT mice following a 12-h fast. n=18-19 per group.

(g) Changes in HR following 24-h fast compared to 12-h in FGF21-deficient mice and acute rhFGF21 treatment.

**Figure S5.**

**
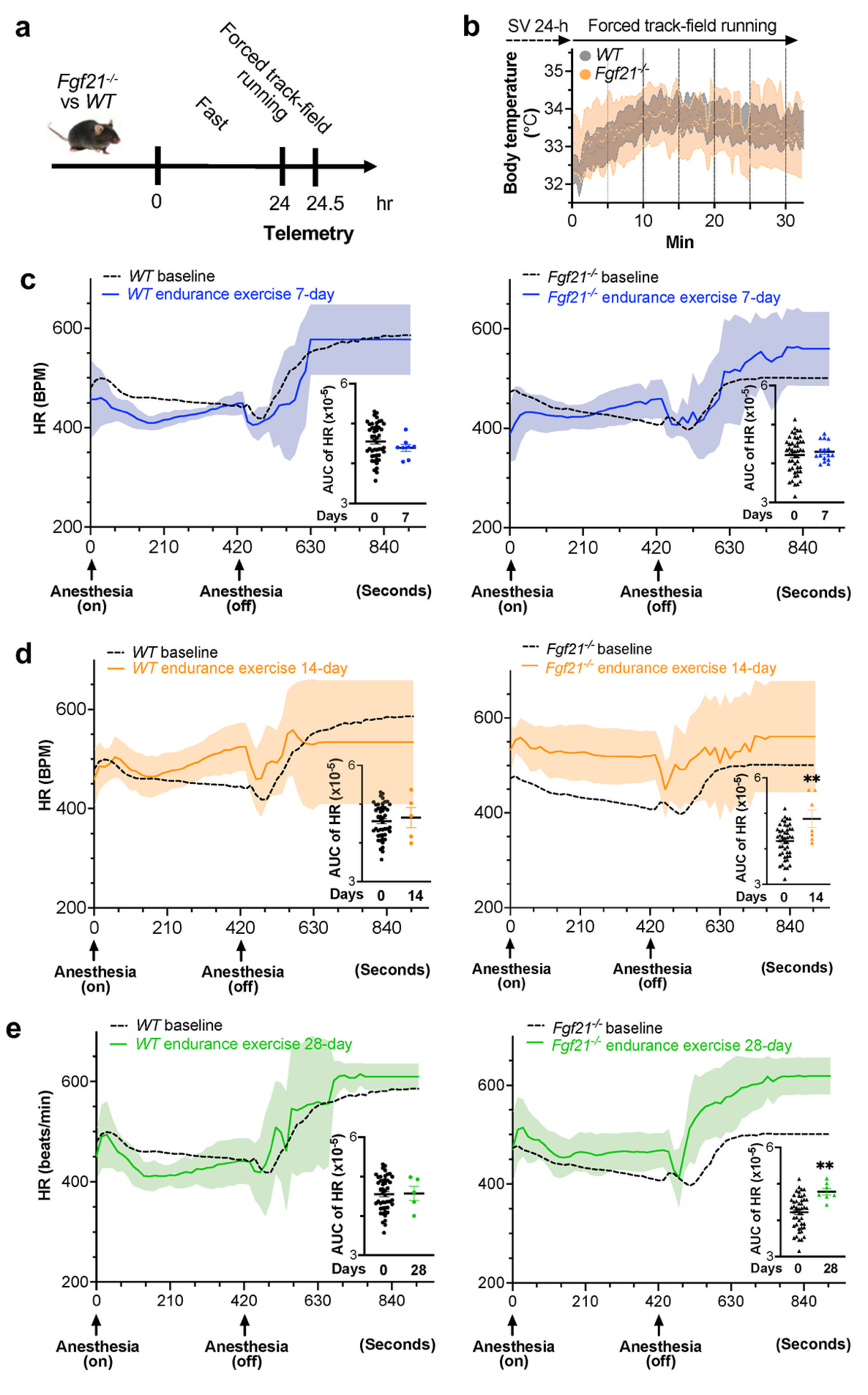
**

**Fig. S5. Effects of physical exertion and endurance training on heart rate in *Fgf21*-null mice.**

**Related to Fig. 2g-2k.**

(**a**) Experimental scheme for assessing the changes in heart function performance following an acute, forced exercise by telemetry in *Fgf21*-null vs WT mice fasted for 24 hours.

(**b**) Insignificant changes in body temperature following 24-h fasting monitored by telemetry in mice as indicated.

(**c**) Changes in HR following a daily 2-h forced track-field endurance exercise for 7 days in mice as indicated. *Inset*, AUC of HR curves.

(**d**) Changes in HR following the endurance exercise for 14 days in mice as indicated. *Inset*, AUC of HR curves.

(**e**) Changes in HR following the endurance exercise for 28 days in *Fgf21*-null vs WT mice. *Inset*, AUC of HR curves.

**Figure S6.**

**
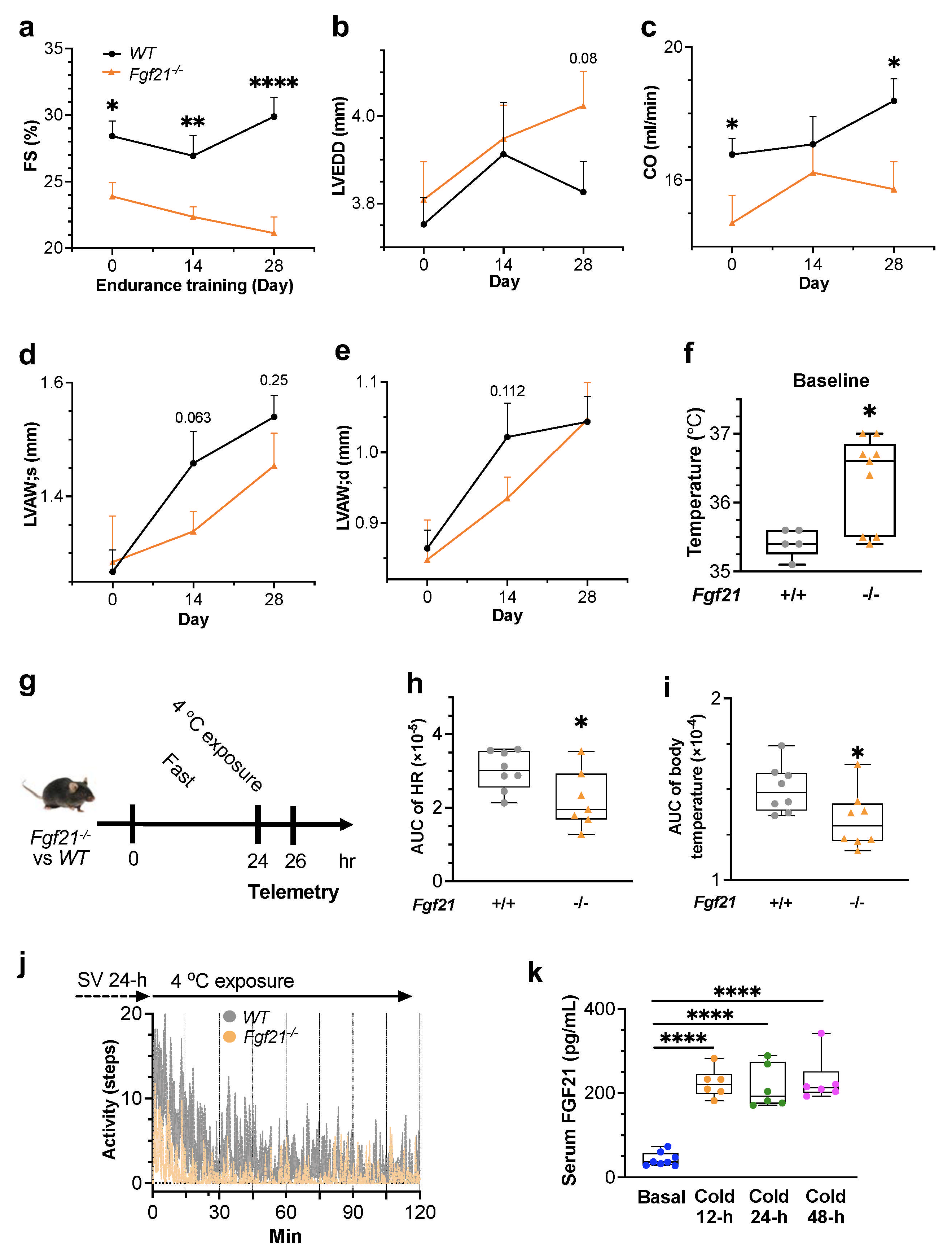
**

**Fig. S6. Effects of cold exposure on heart rate and locomotion in *Fgf21*-null mice.**

**Related to Fig. 2j-2q.**

(**a**-**e**) Changes in FS, LVEDD, CO, LVAW;s and LVAW;d following a daily 2-h treadmill exercise for 28 days in mice as indicated.

(**f**) Basal body temperature in *Fgf21*-null (n=9) vs WT (n=5) mice under normal conditions.

(**g**) Telemetry experimental scheme for monitoring the changes in heart function performance, core body temperature and ambulatory movements following a 2-h exposure to 4 ℃ by telemetry in *Fgf21*-null (n=7-8) vs WT (n=8) mice fasted for 24 hours.

(**h**) AUC analysis of HR curves in Fig. 2o (n=7-8 per group).

(**i**) AUC analysis of core body temperature curves in Fig. 2p (n=8 per group).

(**j**) Ambulatory activity monitored by telemetry for 2 hours following exposure to 4 ℃ in *Fgf21^-/-^* (n=8) vs WT (n=8) mice fasted for 24 hours (n=8 per group).

(**k**) Induction of FGF21 upon cold exposure. Changes in serum FGF21 levels in wildtype mice after 12-h, 24-h, and 48-h cold exposure were measured by ELISA. h, hour. n=6 per group.

**Figure S7.**

**
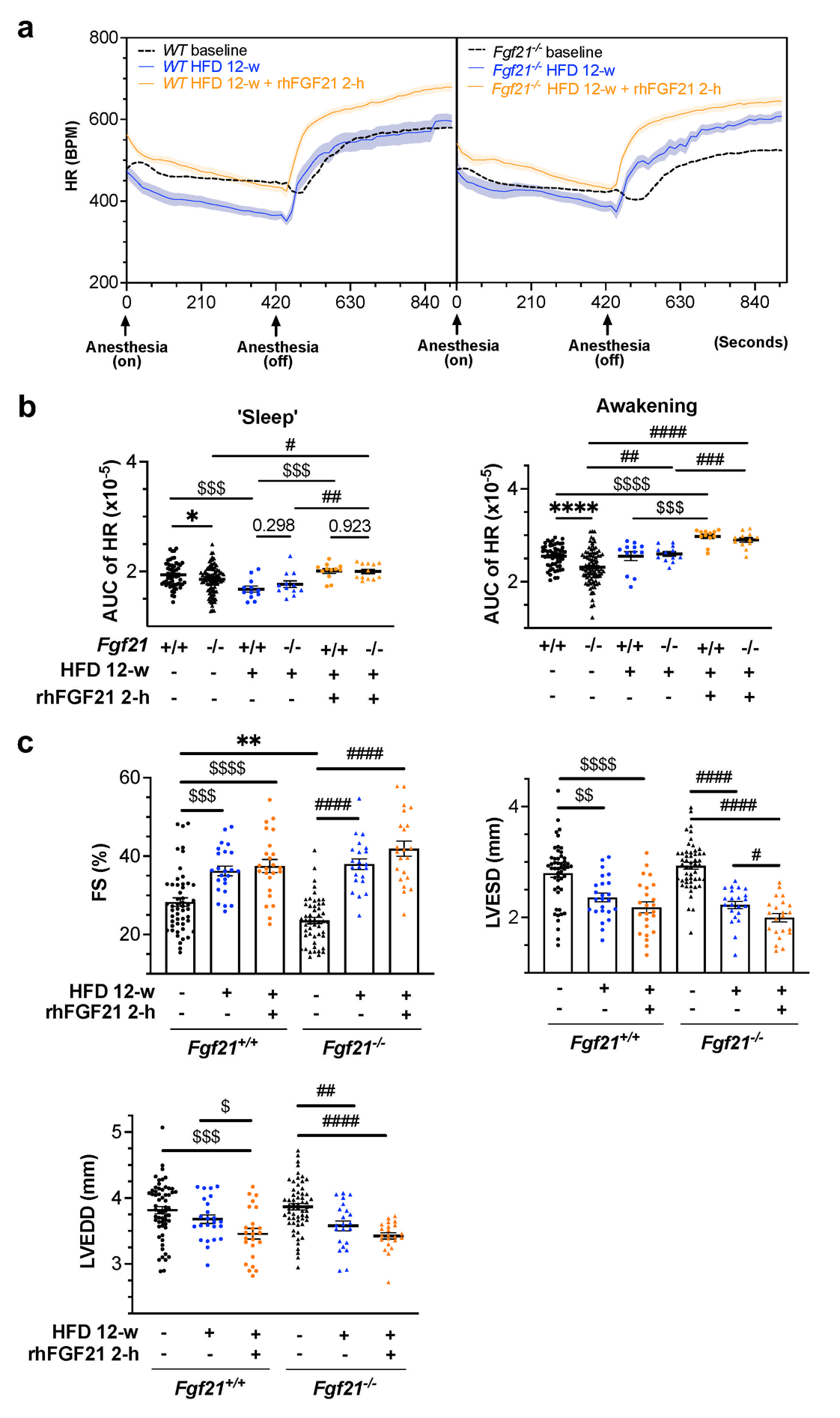
**

**Fig. S7. Effects of high-fat diet on heart rate and contractility in *Fgf21*-null mice and with rhFGF21 treatment.**

**Related to Fig. 3a-3d.**

(**a**) More detailed HR changes as compared to individual groups and to baseline (under normal conditions). See Fig. 3a. w, week.

(**b**) AUC analysis of HR curves in ‘sleep’ and awakening phases as indicated. For AUC analysis of total HR curves, see Fig. 3b.

(**c**) Effects of 12-w HFD on FS, LVESD and LVEDD in *Fgf21^-/-^* (n=8) vs WT (n=8) mice and following 2-h rhFGF21 treatment.

**Figure S8.**

**
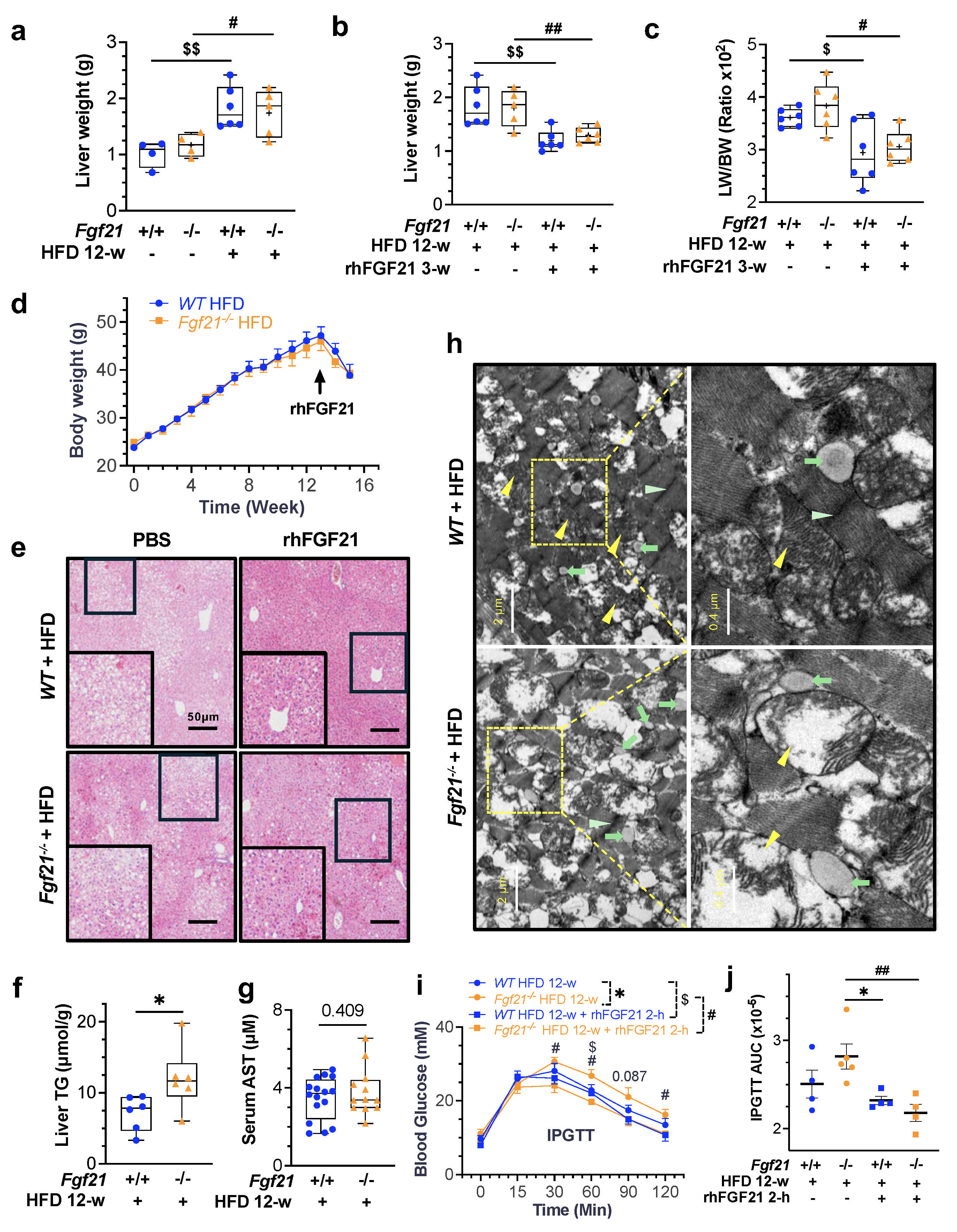
**

**Fig. S8. Effects of high-fat diet on hepatic and serum metabolic parameters and on cardiac mitochondria in *Fgf21*-null mice and with rhFGF21 treatment.**

**Related to Fig. 3e-3f.**

**Results related to effects of mild HFD feeding on heart energetic performance (see Fig. 3a-3f):**

Unlike chronic, obesity-causing HFD consumption (usually more than 14 weeks), we employed a mild regime of 12-week HFD feeding. Despite similar body and liver weights, mild HFD-fed *Fgf21^-/-^* mice had mildly elevated serum triglycerides (TG), FFAs, and hepatic TG, without changing liver damage marker AST (Fig. 3e-3f and S8a-S8g). Ultrastructure analysis revealed increased lipid droplets associating with mitochondria without significant mitochondrial improvements in the hearts of HFD-fed *Fgf21^-/-^* mice compared to normal conditions or WT hearts (Fig. S8h). Additionally, *Fgf21*-null mice displayed slight glucose intolerance but greater sensitivity to rhFGF21 treatment (Fig. S8i-S8j). These mild systemic metabolic changes set a relatively more physiologically relevant condition for assessing cardiac energetic performance under nutrient repletion than an overt pathological obese condition.

(**a**) Effects of 12-w HFD on liver weights in gram (g) in *Fgf21^-/-^* (n=4-6) vs WT (n=4-5) mice. W, week.

(**b**) Effects of 3-w FGF21 treatment on HFD-fed *Fgf21^-/-^* (n=5-6) vs WT (n=5-6) mice.

(**c**) Changes in liver weight (LW) relative to body weight (BW) in mice as in b.

(**d**) Changes in body weight in HFD-fed *Fgf21^-/-^* (n=6) vs WT (n=6) mice and mice treated with rhFGF21 for 3 weeks.

(**e**) Representative changes in liver fat accumulation in HFD-fed *Fgf21^-/-^* (n=6) vs WT (n=6) mice after 3-w rhFGF21 treatment.

(**f**) Changes in liver TG content in *Fgf21^-/-^* vs WT mice (n=6 per group) after HFD feeding for 12 weeks.

(**g**) Changes in serum AST levels in *Fgf21^-/-^* (n=12) vs WT (n=16) mice after HFD feeding for 12 weeks.

(**h**) Changes in cardiac myofiber ultrastructure and interfibrillar mitochondria in in *Fgf21^-/-^* vs WT mice (n=6 per group) following a 12-w HFD feeding (n=6 for each group). Yellow arrowhead, mitochondria. Cyan arrowhead, Z line. Green arrow, lipid droplet. There appeared to be more lipid droplets in cardiac myofibrillar tissue in *Fgf21*-null mice.

(**i**) IPGTT in the same groups as in Fig. 3a.

(**j**) AUC analysis for glucose excursion curves from IPGTT, shown in (i).

**Figure S9.**

**
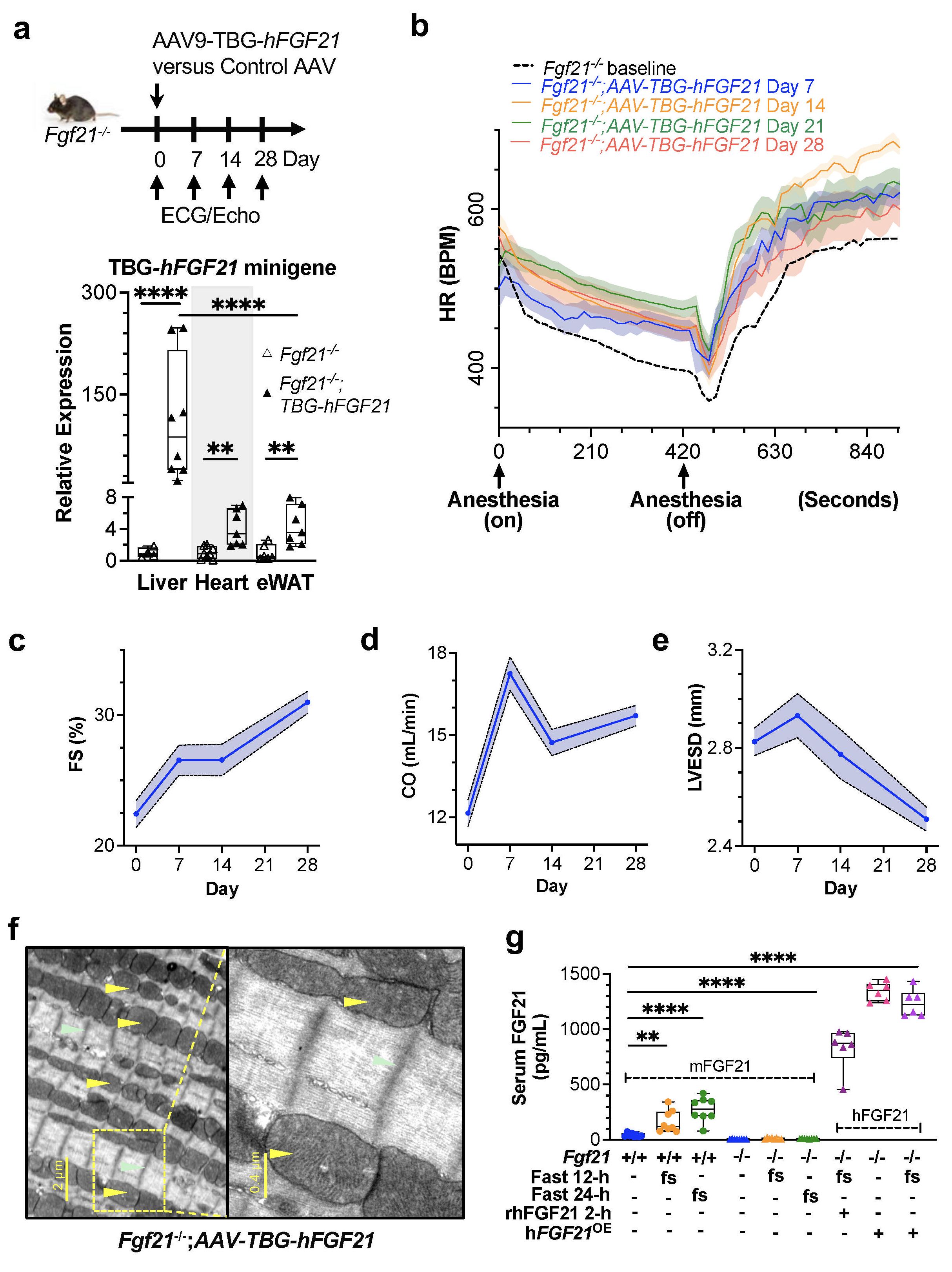
**

**Fig. S9. Effects of FGF21 restoration on heart function performance in *Fgf21*-null mice.**

**Related to Fig. 3g-3i.**

(**a**) Upper, experimental scheme for ECG and Echo analyses following AAV9-mediated overexpression of human FGF21 (hFGF21) via TBG promoter-driven minigene (AAV-TBG-*hFGF21*) in FGF21-deficient mice. Empty vector serves as a control. Lower, AAV-driven hFGF21-HA expression in mouse livers two weeks post-infection compared to that in mouse hearts and eWATs, analyzed by qPCR.

(**b**) Time-dependent changes in HR excursion in hFGF21-overexpressing *Fgf21*-null mice.

(**c**-**e**) Time-dependent changes in FS, CO, and LVESD in hFGF21-overexpressing *Fgf21*-null mice.

(**f**) Normalization of cardiac mitochondria ultrastructure after FGF21 restoration. Yellow arrowhead, mitochondria. Cyan arrowhead, Z line.

(**g**) Changes in serum FGF21 levels in mice with FGF21 deficiency, rhFGF21 treatment, hFGF21 overexpression under basal and fasting conditions, measured by ELISA. n=6-8 per group. mFGF21, mouse FGF21. hFGF21, human FGF21. OE, overexpression.

**
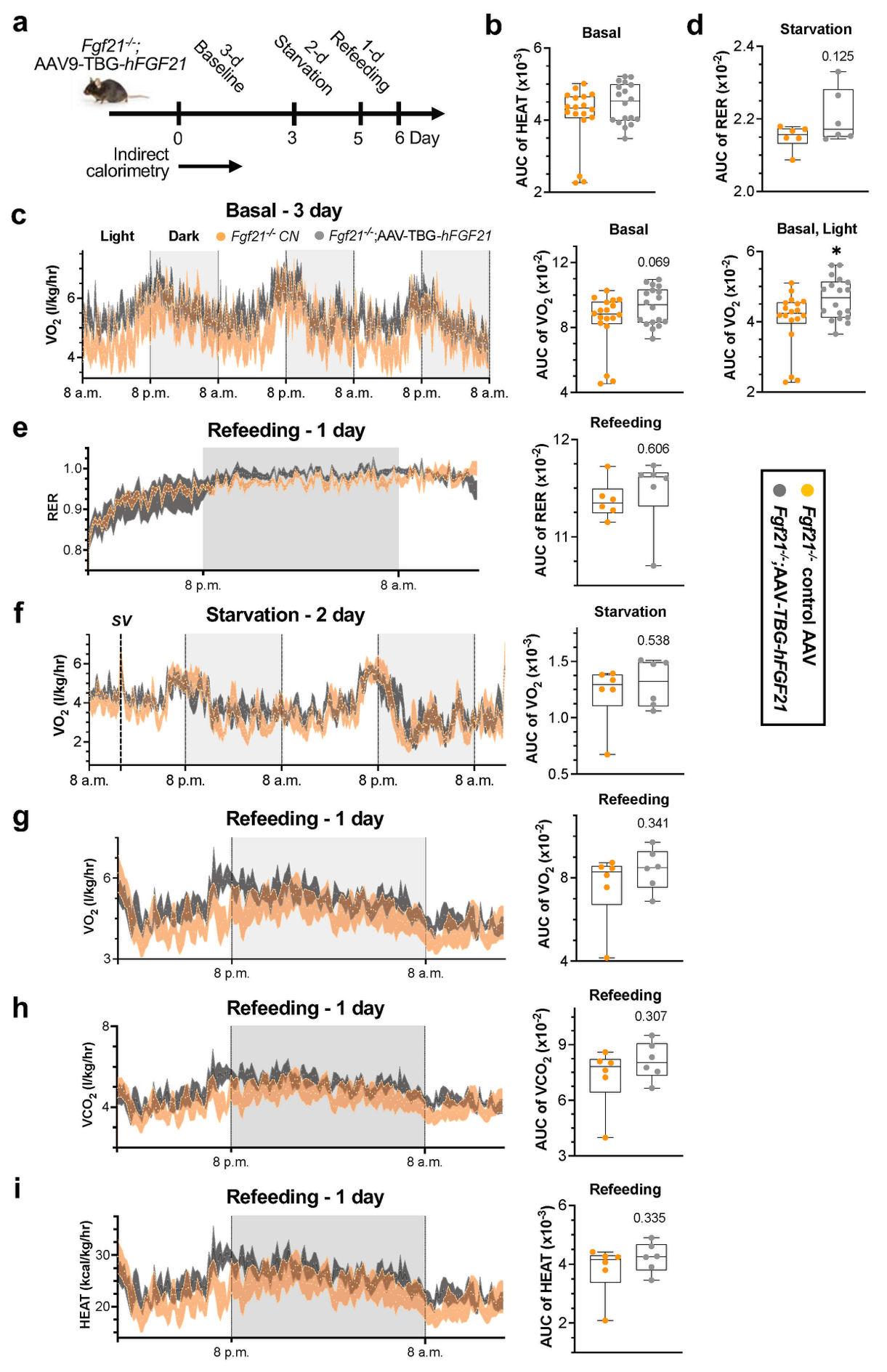
Figure S10.**

**Fig. S10. Effects of FGF21 restoration on energy expenditure during basal starvation, and refeeding periods in *Fgf21*-null mice.**

**Related to Fig. 3j-3p** and **S11.**

**Results related to energy expenditure and substrate utilization in association with heart energetic performance (**see **Fig. 3j-3p** and **next S11):**

Indirect calorimetry revealed that FGF21 restoration increased RER from approximately 0.7 to 0.8 under normal caloric conditions (Fig. 3j and S10a), indicating a shift from fat to mixed substrate utilization, which was even more drastic than that of the wildtype mice (Fig. S11a), suggesting maintaining a constantly high FGF21 level accelerates energy expenditure. Resting energy expenditure (HEAT) also increased, especially during the light phase upon FGF21 restoration (Fig. 3j, S10b and S11a). Basal VCO_2_, light-phase VO_2_ (Fig. S10c), food intake (Fig. 3l), and water consumption increased (Fig. 3m), reflecting higher metabolic rate and energy expenditure caused by FGF21 overexpression, consistent with adaptive HR reduction upon FGF21 loss. This is generally in contrast to wildtype mice with basal or low levels of FGF21 (Fig. S11a and S11d). Given the more significant changes of VCO_2_ than VO_2_ (Fig. 3j), these findings indicate that FGF21 loss disrupts the TCA cycle for oxidative metabolism. In contrast, FGF21 overexpression enhances energy expenditure and shifts energy reliance from predominantly fat and glycolysis (also Fig. 4a) to mixed substrate oxidation, promoting effective energy use, cardiac energy competency, and thus, HR adaptation.

During 2-day starvation, RER curves flattened and dropped below 0.7 in both FGF21-null and -restored mice, indicating a shift to fat utilization, especially on the second day, as freely glucose and available amino acids/proteins became depleted or more limited (Fig. 3k and S10d). Notably, FGF21-restored mice continued to consume all macronutrients during the first active phase, while *Fgf21*-null mice remained inflexible. By contrast, the wildtype mice, which typically have elevated circulating FGF21 induced by starvation, maintained a significantly higher RER throughout (Fig. S11b). During 1-day refeeding, macronutrient utilization increased, but *Fgf21*-null mice still showed a trend towards higher fat-craving (Fig. S10e and S11c). Throughout the 2-day starvation and 1-day refeeding, VO_2_, VCO_2_, energy expenditure, and water consumption trended higher in FGF21-restored mice, particularly during the first active phase, though without statistical significance (Fig. 3k, 3n-3p and S10f-S10i). Food intake was compensatively higher in *Fgf21*-null mice during refeeding, indicating a lower energy-producing capacity.

(**a**) Experimental scheme for indirect calorimetry in hFGF21-overexpressed *Fgf21*-null mice in response to 2-day starvation and then 1-day refeeding. *Fgf21*-null (*Fgf21^-/-^*) mice with control AAV serves as the experimental control.

(**b**) AUC analysis of total (light phase + dark phase) basal HEAT curves shown in Fig. 3j, n=18 per group.

(**c**) Effects of FGF21 restoration on VO_2_ under 3-day basal conditions in *Fgf21*-null mice, n=18 per group. Middle, Total AUC; Right, light phase AUC.

(**d**) AUC (total) analysis of RER curves during 2-day starvation, n=6 per group.

(**e**) Comparative RER excursions during 1-day refeeding following 2-day starvation. Right, total AUC.

(**f**) Comparative VO_2_ excursions during 2-day starvation. Right, total AUC.

(**g**) Comparative VO_2_ excursions during 1-day refeeding. Right, total AUC.

(**h**) Comparative VCO_2_ excursions during 1-day refeeding. Right, total AUC.

(**i**) Comparative HEAT excursions during 1-day refeeding. Right, total AUC.

**Figure S11.**

**
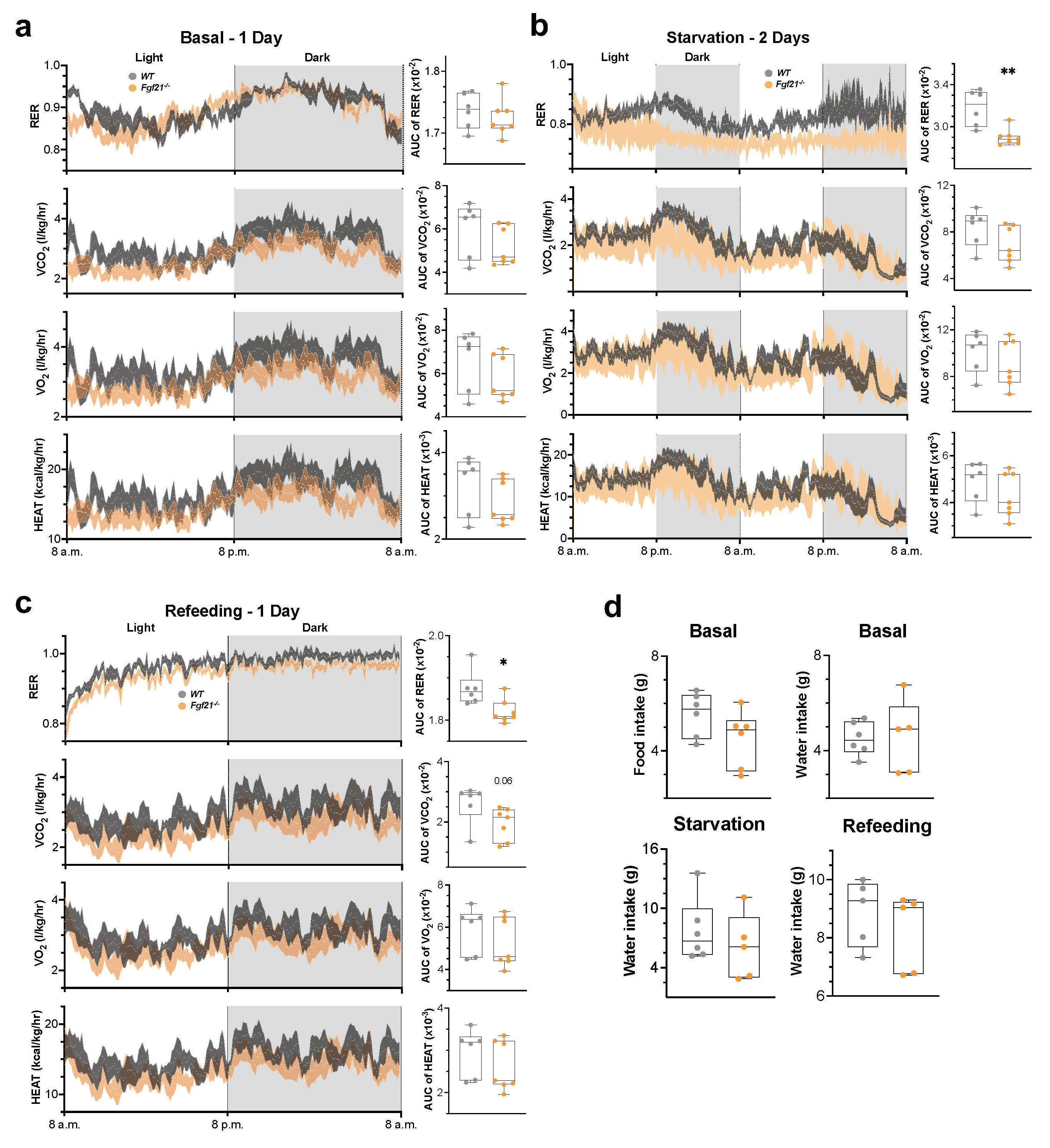
**

**Fig. S11. Changes in energy expenditure and substrate utilization during basal, starvation and refeeding periods in *Fgf21*-null mice.**

**Related to Fig. 3j-3p** and **S11.**

(**a**-**c**) Energy expenditure (RER, VCO_2_, VO_2_, HEAT) by indirect calorimetry under basal, 48 hours of starvation, and 24 hours of refeeding in FGF21-null (*Fgf21^-/-^*) and wildtype (WT) mice. See Fig. S10a for a similar experimental design. Left, excursion curves. Right, AUC of the respective curve. AUC plot without a title indicates total (light + dark) AUC. n=6 per group.

(**d**) Changes in food intake and water consumption in the same groups and conditions as in a-c.

Data are means ± s.e.m.s; (a-d) two-tailed unpaired Student’s t-test.

**Figure S12.**

**
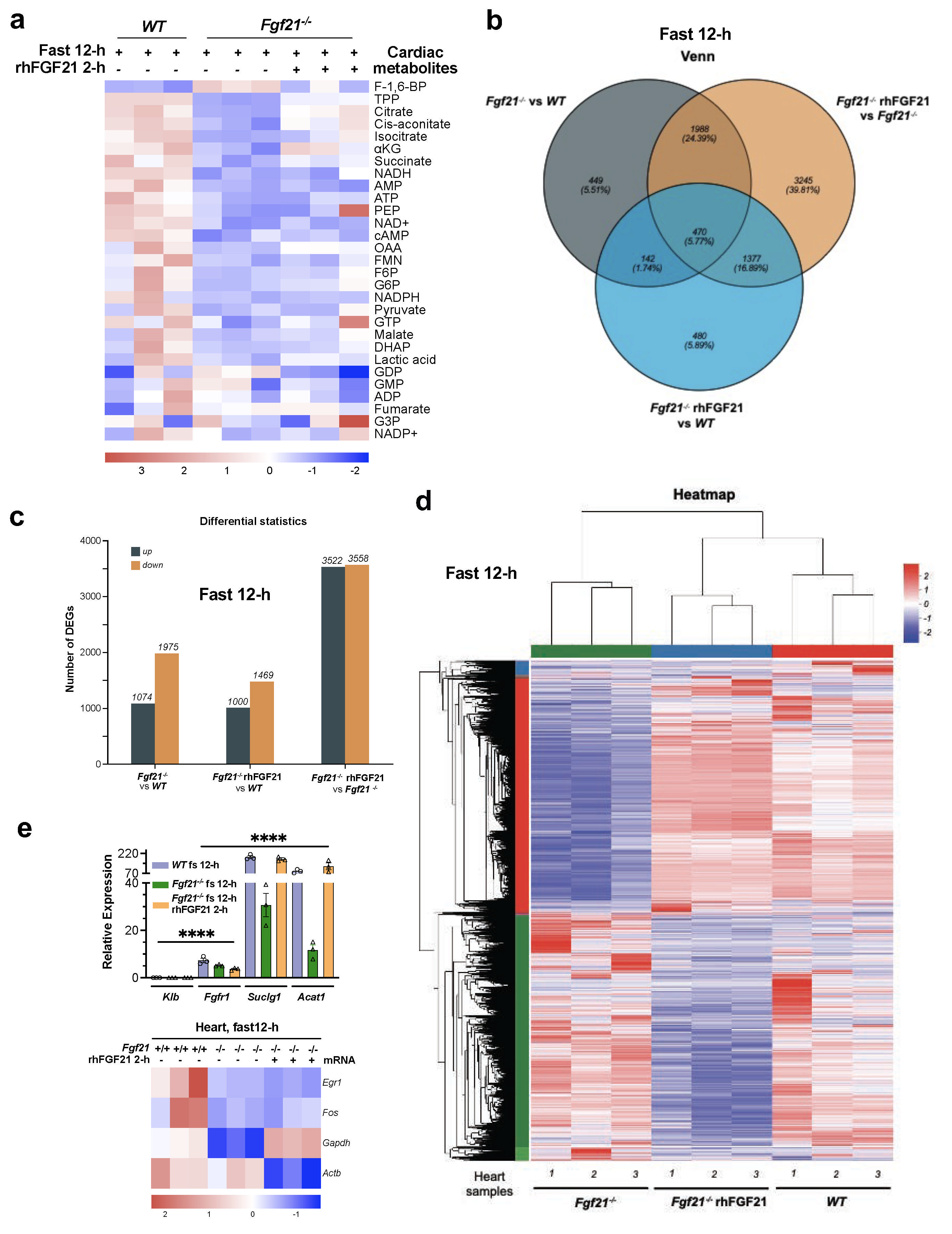
**

**Fig. S12. Effects of FGF21 deficiency and restoration on mitochondrial energy metabolism during fast revealed by targeted cardiac energy metabolomics and transcriptomics.**

**Related to Fig. 4a-4e.**

(**a**) Heatmap of changes in cardiac metabolites of glycolysis, TCA cycle, ETC, and OXPHOS in FGF21-deficient mice compared to acute 2-h rhFGF21 treatment and WT mice after a 12-h fast.

(**b**) Venn diagram illustrating cardiac differential gene expression changes among fasted *Fgf21*-null, rhFGF21 treatment, and WT mice.

(**c**) Bar graph illustrating the differential statistics of DEGs.

(**d**) Heatmap of differential cardiac gene expression in fasted *Fgf21*-null, rhFGF21 treatment, and WT mice.

(**e**) Upper, negligible expression of *Klb* compared to *Fgfr1* and other more abundant metabolic genes in the heart under conditions as indicated. Lower, changes of cardiac EGR1 and c-FOS levels in response to rhFGF21 treatment in the heart of FGF21-null mice, compared to wildtype mice under 12-h fast. n=3 per group.

**Figure S13.**

**
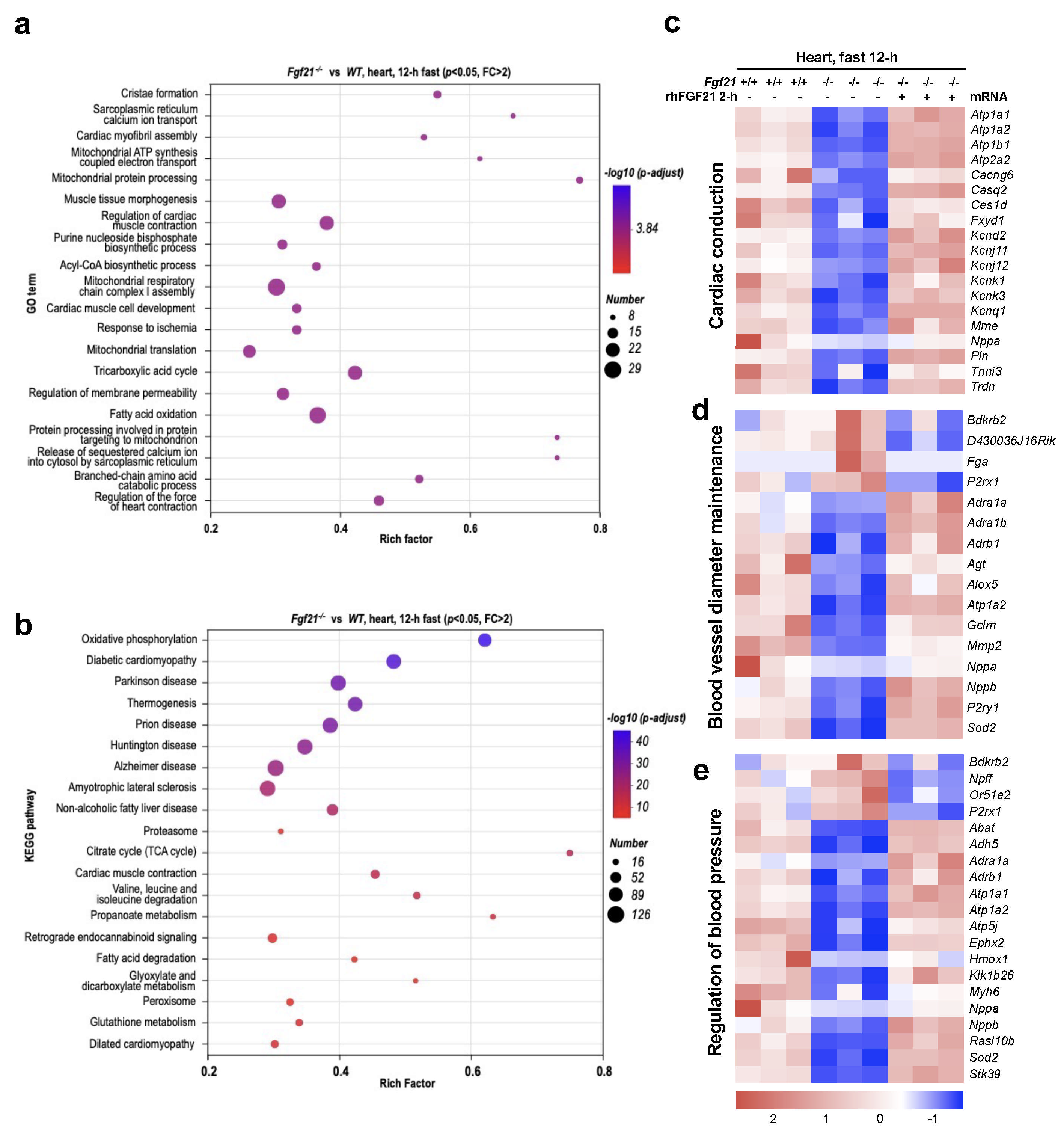
**

**Fig. S13. Effects of FGF21 deficiency and restoration on cardiac gene expression involved in mitochondrial energy metabolism and heart function during fast revealed by transcriptomics.**

**Related to Fig. 4a-4e.**

(**a**) GO-term pathway enrichment (bubble plot) revealing the most significant changes in cardiac mitochondrial biogenesis, TCA cycle, ETC (e.g., complex I), OXPHOS, membrane permeability, substrate catabolism (e.g., FAO, BCAA catabolism), and cardiac muscle contraction and function in fasted *Fgf21*-null vs WT mice. *p* < 0.05, |FC| > 2, n=3 per group.

(**b**) KEGG pathway enrichment (bubble plot) revealing the most significant vulnerabilities to developing cardiomyopathies and other metabolic diseases, along with the topmost significant changes in pathways for energy metabolism, heart contraction, and proteasome in fasted *Fgf21*-null vs WT mice.

(**c**) Transcriptomic enrichment heatmap of cardiac conduction pathway due to FGF21 deficit and restoration in the indicated mouse groups.

(**d**) Transcriptomic enrichment heatmap of blood vessel diameter maintenance pathway due to FGF21 deficit and restoration in the indicated mouse groups.

(**e**) Transcriptomic enrichment heatmap of blood pressure regulation pathway due to FGF21 deficit and restoration in the indicated mouse groups.

**Figure S14.**

**
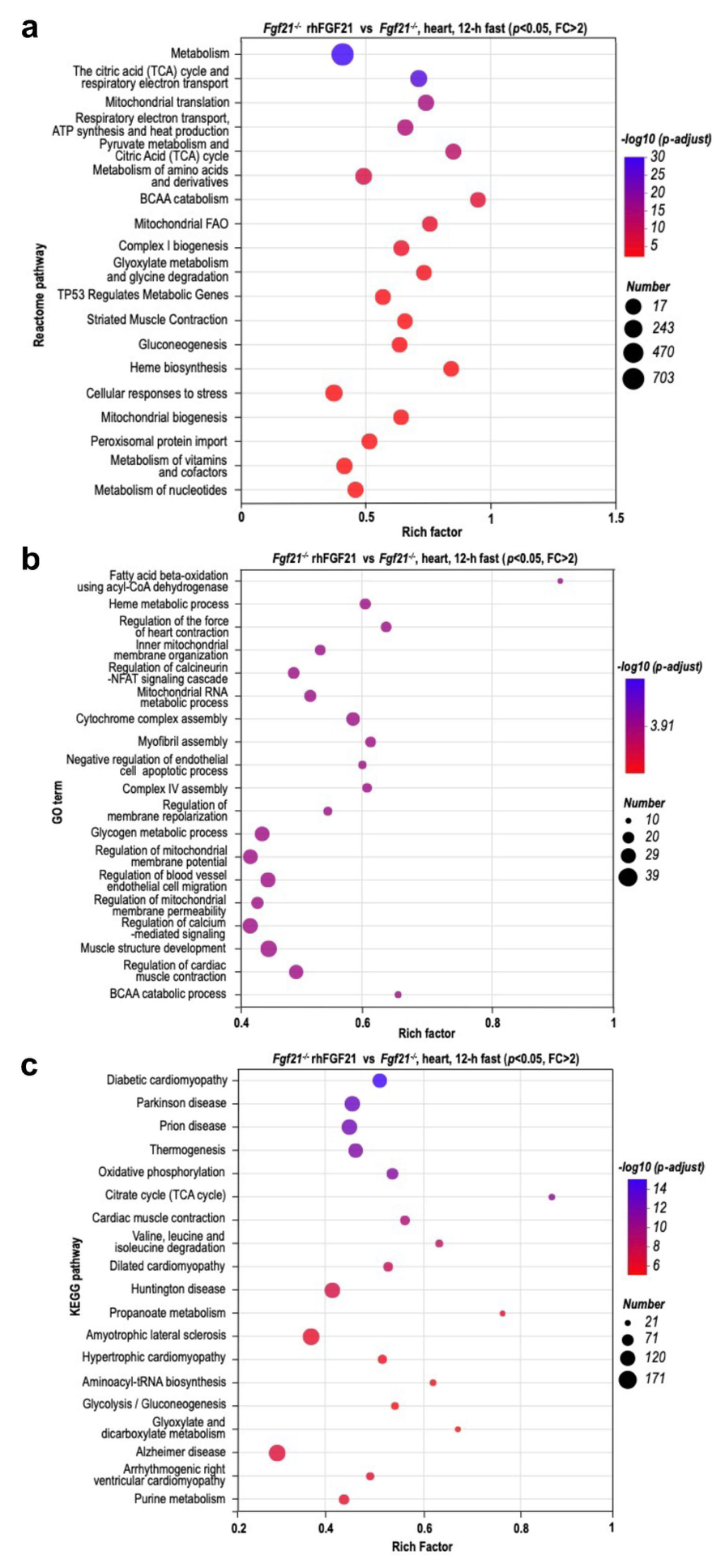
**

**Fig. S14. Effects of FGF21 restoration on cardiac gene expression involved in mitochondrial energy metabolism and heart function during fast revealed by transcriptomics.**

**Related to Fig. 4a-4e.**

(**a**) Reactome pathway enrichment (bubble plot) revealing the most significant improvements in cardiac mitochondrial biogenesis, TCA cycle, ETC (e.g., complex I), OXPHOS, membrane permeability, substrate catabolism (e.g., FAO, BCAA catabolism, glucose and nucleotide metabolism), striated muscle contraction, and stress response in fasted *Fgf21*-null mice following rhFGF21-treatment. *p* < 0.05, |FC| > 2, n=3 per group.

(**b**) GO-term pathway enrichment (bubble plot) also revealing the most significant improvements in substrate catabolism (e.g., FAO, BCAA catabolism, glucose metabolism), cardiac muscle contraction and function, hemodynamic function (vasculature effects), mitochondrial membrane permeability, biogenesis, ETC (e.g., complex III and IV) in fasted *Fgf21*-null mice following rhFGF21-treatment.

(**c**) KEGG pathway enrichment (bubble plot) also revealing the most significant the most significant improvements in vulnerabilities to developing cardiomyopathies and other metabolic diseases, along with the most significant changes in pathways for energy metabolism, heart contraction, glycolysis, and purine metabolism in fasted *Fgf21*-null mice following rhFGF21-treatment.

**Figure S15.**

**
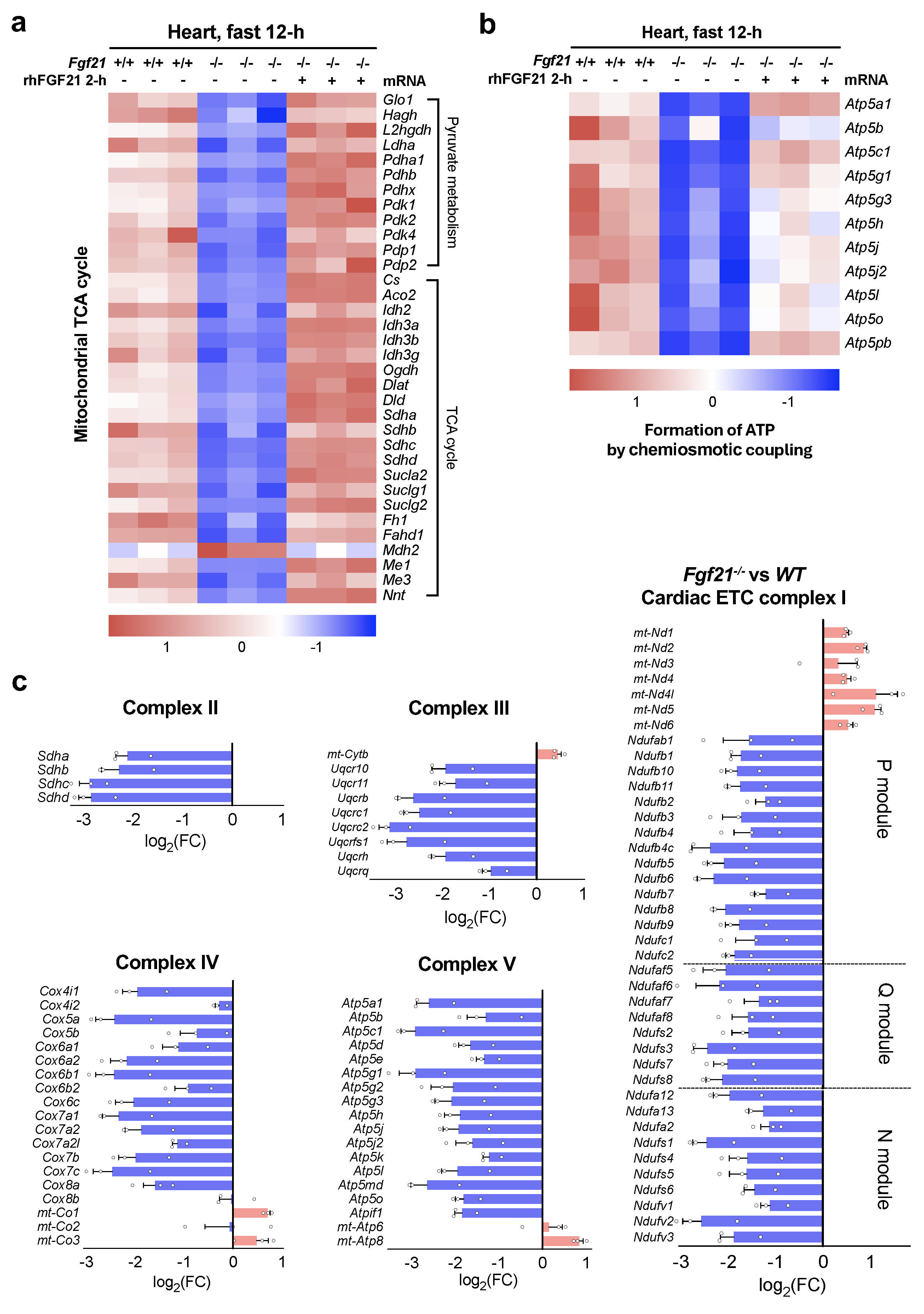
**

**Fig. S15. Effects of FGF21 deficiency and restoration on cardiac gene expression involved in mitochondrial TCA cycle, ETC, and OXPHOS during fast revealed by transcriptomics.**

**Related to Fig. 4a-4c.**

(**a**) Transcriptomic enrichment heatmap of mitochondrial TCA cycle changes due to FGF21 deficit and restoration in the indicated mouse groups.

(**b**) Transcriptomic enrichment heatmap of mitochondrial ATP synthesis pathway changes due to FGF21 deficit and restoration in the indicated mouse groups.

(**c**) Transcriptomic enrichment heatmap of mitochondrial ETC complexes I to IV changes due to FGF21 deficit in the indicated mouse groups.

**Figure S16.**

**
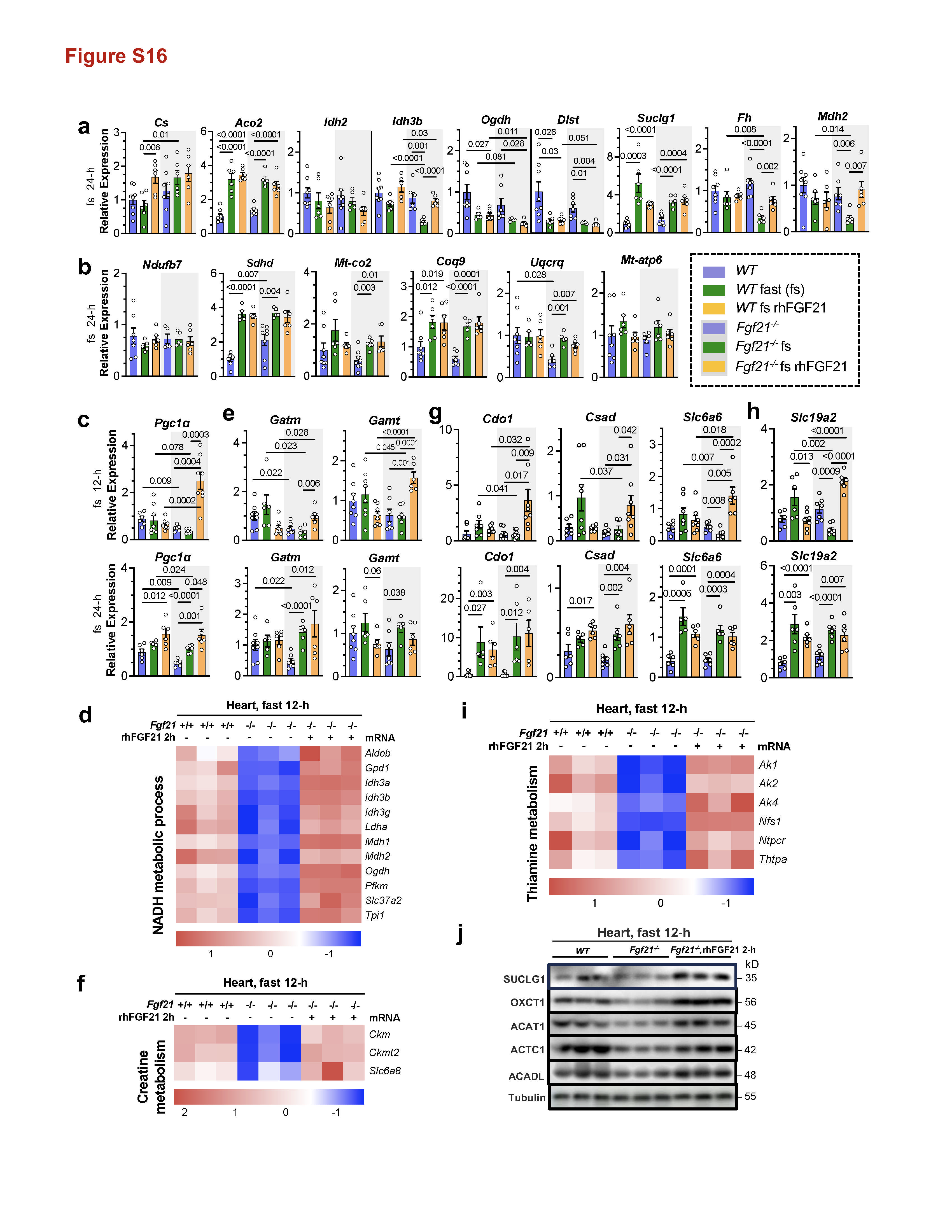
**

**Fig. S16. Effects of FGF21 deficiency and restoration on cardiac gene expression involved in mitochondrial TCA cycle, ETC, and OXPHOS during fast.**

**Related to Fig. 4a-4c.**

(**a**) qRT-PCR analysis for the expression of representative cardiac genes involved in TCA cycle in *Fgf21*-null vs WT mice under basal, 24-h fast (fs), and then 2-h rhFGF21 treatment conditions.

(**b**) qRT-PCR analysis for the expression of representative cardiac genes involved in ETC complexes in *Fgf21*-null vs WT mice under conditions as in a.

(**c**) qRT-PCR analysis for cardiac *Pgc1⍺* expression in *Fgf21*-null vs WT mice under basal, 12-h vs 24-h fast, and then 2-h rhFGF21 treatment conditions.

(**d**) Transcriptomic enrichment heatmap of NADH generation reactions due to FGF21 deficit and restoration in the indicated mouse groups.

(**e**) qRT-PCR analysis for cardiac *Gatm* and *Gamt* expression involved in creatine/creatinine synthesis in *Fgf21*-null vs WT mice under conditions as in c.

(**f**) Transcriptomic enrichment heatmap of creatine metabolism genes due to FGF21 deficit and restoration in the indicated mouse groups.

(**g**) qRT-PCR analysis for the expression of taurine synthesis and transport genes in *Fgf21*-null vs WT mice under conditions as in c.

(**h**) qRT-PCR analysis for the expression of cardiac thiamine transporter *Slc19a2* gene under conditions as in c.

(**i**) Transcriptomic enrichment heatmap of thiamine metabolism genes due to FGF21 deficit and restoration in the indicated mouse groups.

(**j**) Western blot analysis confirms transcriptomic and qPCR data for the expression of cardiac genes as indicated. n=3 per group.

**Figure S17.**

**
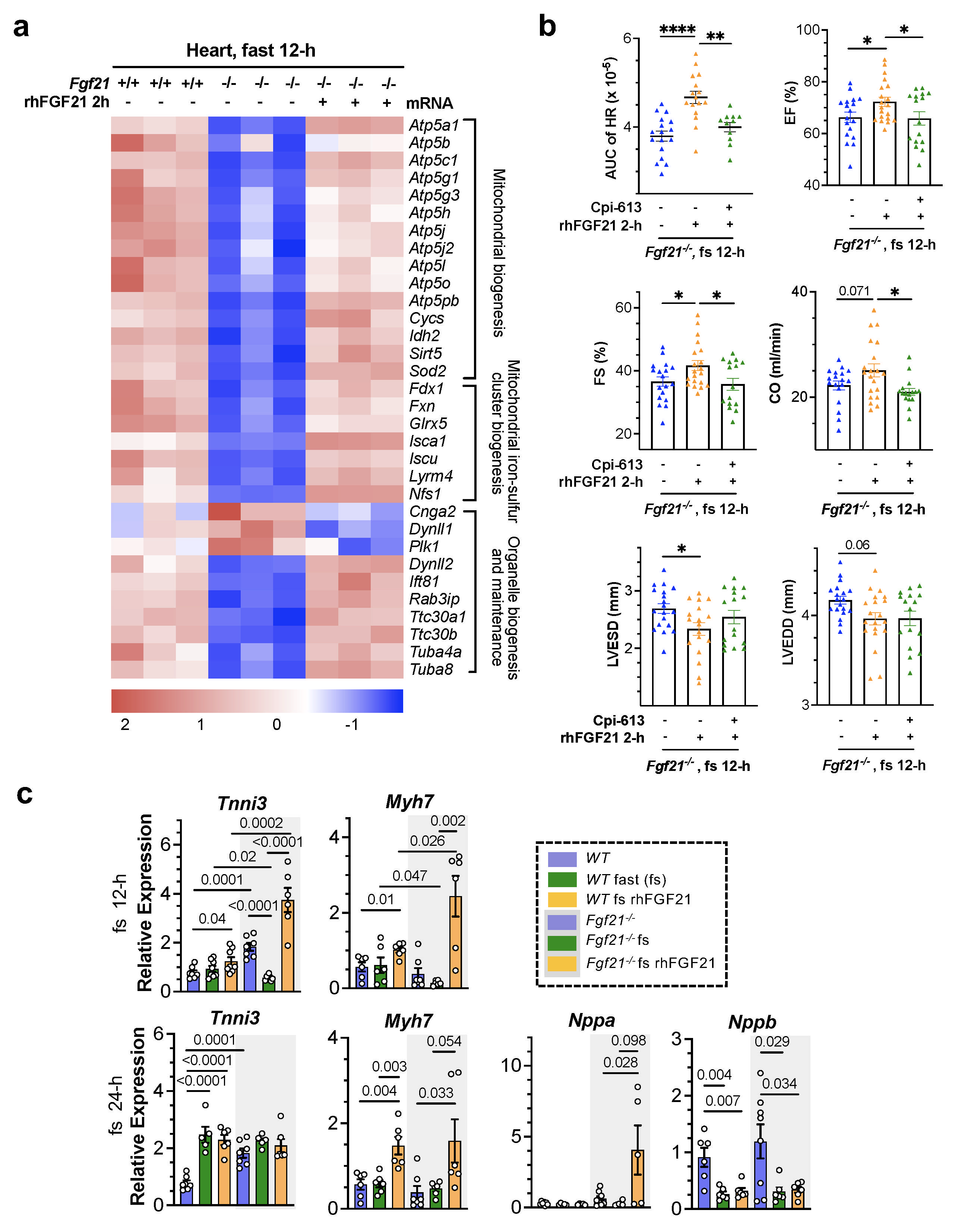
**

**Fig. S17. Effects of FGF21 deficiency and restoration on cardiac mitochondrial biogenesis and heart function during fast.**

**Related to Fig. 4.**

(**a**) Transcriptomic enrichment heatmap of mitochondrial biogenesis, iron-sulfur cluster biogenesis, and other organelle biogenesis, transport and maintenance due to FGF21 deficit and restoration in the indicated mouse groups.

(**b**) Effects of Cpi-613 in rhFGF21-promoted improvements of Echo parameters EF, FS, CO, LVEDD and LVESD in *Fgf21*-null mice fasted for 12 hours.

(**c**) qRT-PCR analysis for the expression of cardiac genes involved in heart rate and contraction in *Fgf21*-null vs WT mice under basal, 12-h vs 24-h fast, and then 2-h rhFGF21 treatment conditions.

**Figure S18.**

**
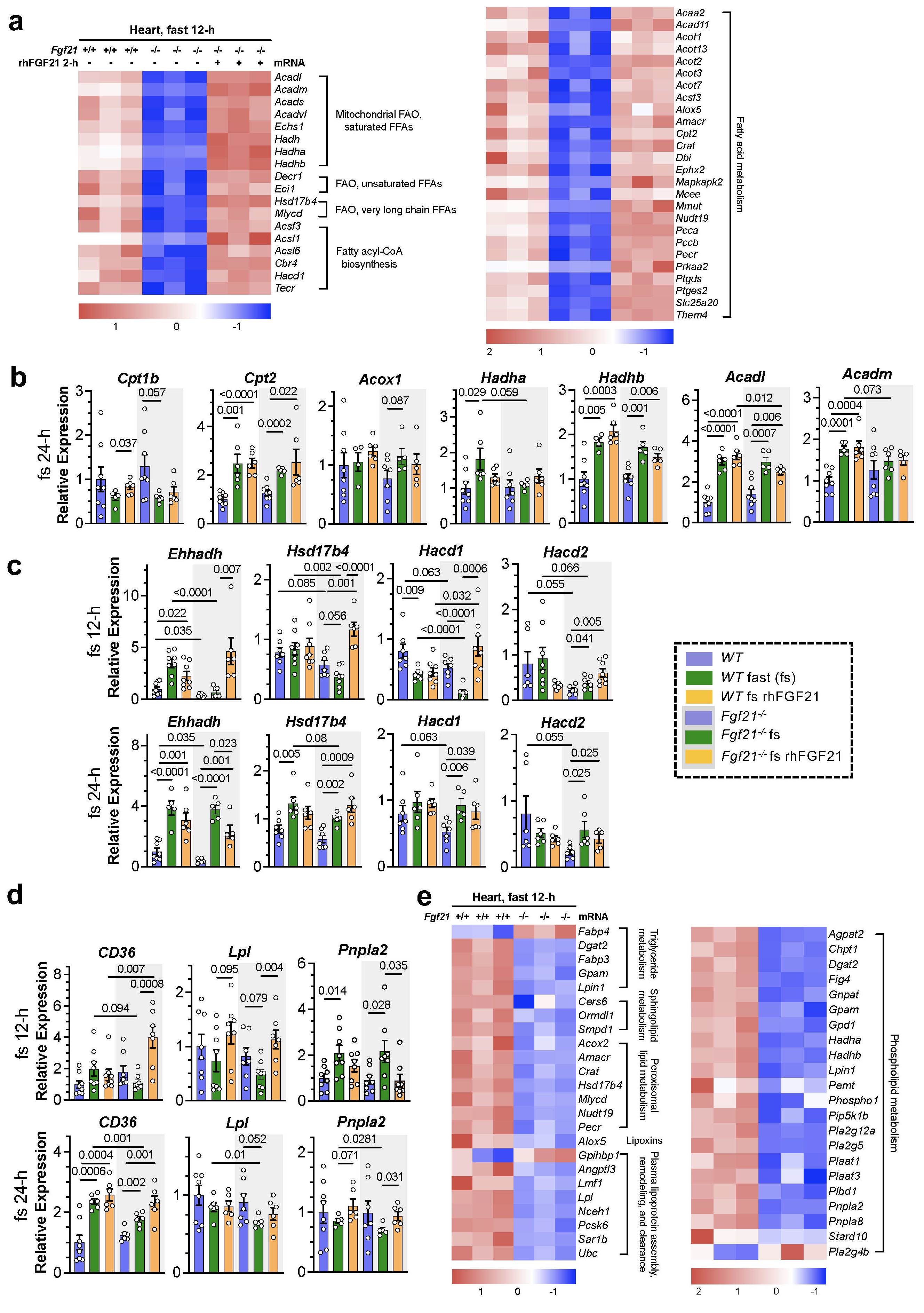
**

**Fig. S18. Effects of FGF21 deficiency and restoration on cardiac mitochondrial FAO and lipid (TG and phospholipids) metabolism during fast.**

**Related to Fig. 5a-5b.**

(**a**) Transcriptomic enrichment heatmap of fatty acid metabolism, particularly mitochondrial FAO, due to FGF21 deficit and restoration in the indicated mouse groups.

(**b**) qRT-PCR analysis for the expression of cardiac genes involved in FAO in *Fgf21*-null vs WT mice under basal, 24-h fast (fs), and then 2-h rhFGF21 treatment conditions.

(**c**) qRT-PCR analysis for the expression of representative cardiac genes involved peroxisome- and ER-associated FAO, which are linked to congenital cardiomyopathy, in *Fgf21*-null vs WT mice under basal, 12-h vs 24-h fast (fs), and then 2-h rhFGF21 treatment conditions.

(**d**) qRT-PCR analysis for the expression of cardiac genes for lipid importer and lipolysis in the indicated groups as in b.

(**e**) Transcriptomic enrichment heatmap of cardiac pathway genes for TG metabolism, sphingolipid metabolism, peroxisomal lipid metabolism, phospholipid metabolism, and plasma lipoprotein assembly, remodeling and clearance due to FGF21 deficit and restoration in the indicated mouse groups.

**Figure S19.**

**
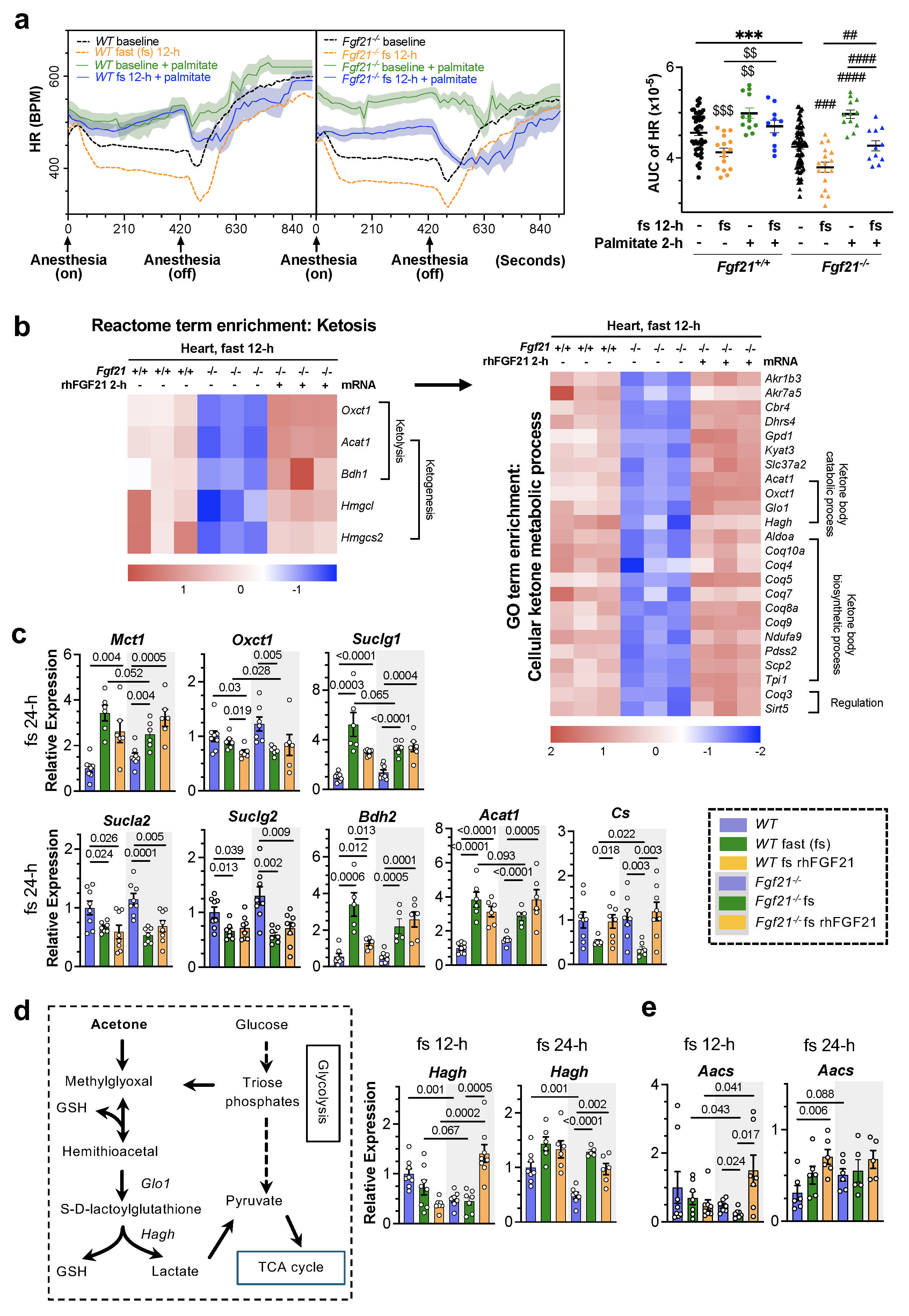
**

**Fig. S19. Roles of FGF21-regulated mitochondrial FAO and ketolysis in cardiac energetic performance during fast.**

**Related to Fig. 5c-5e.**

(**a**) More detailed comparisons for the effects of palmitate supplementation on HR in FGF21-deficient vs WT mice, under basal and 12-h fast (fs) conditions (n=10-12 for each group and each condition). See Fig. 5c.

(**b**) Left, Reactome pathway enrichment of cardiac ketolysis and ketogenesis (BHB and acetoacetate) due to FGF21 deficit and restoration in the indicated mouse groups. Right, GO-term enrichment of cardiac ketone body metabolic processes (catabolism and anabolism) due to FGF21 deficit and restoration in the indicated mouse groups.

(**c**) qRT-PCR analysis for the expression of cardiac ketolysis (BHB and acetoacetate) genes in *Fgf21*-null vs WT mice under basal, 24-h fast, and then 2-h rhFGF21 treatment conditions.

(**d**) Left, sketch of a common degradation pathway for acetone (a form of ketone body) and glycolysis byproduct methylglyoxal via S-D-lactoylglutathione to GSH and lactate, latter of which may feed into the TCA cycle. The image is generated in PowerPoint. Right, qRT-PCR analysis for the expression of cardiac *Hagh* gene in *Fgf21*-null vs WT mice under basal, 12-h vs 24-h fast, and then 2-h rhFGF21 treatment conditions. See Figure S19B, right panel for the expression of cardiac *Glo1*.

(**e**) qRT-PCR analysis for the expression of cardiac *Aacs* gene in *Fgf21*-null vs WT mice under basal, 12-h vs 24-h fast, and then 2-h rhFGF21 treatment conditions. AACS catalyzes the conversion of acetoacetate (another form of ketone body) to acetyl-CoA.

**Figure S20.**

**
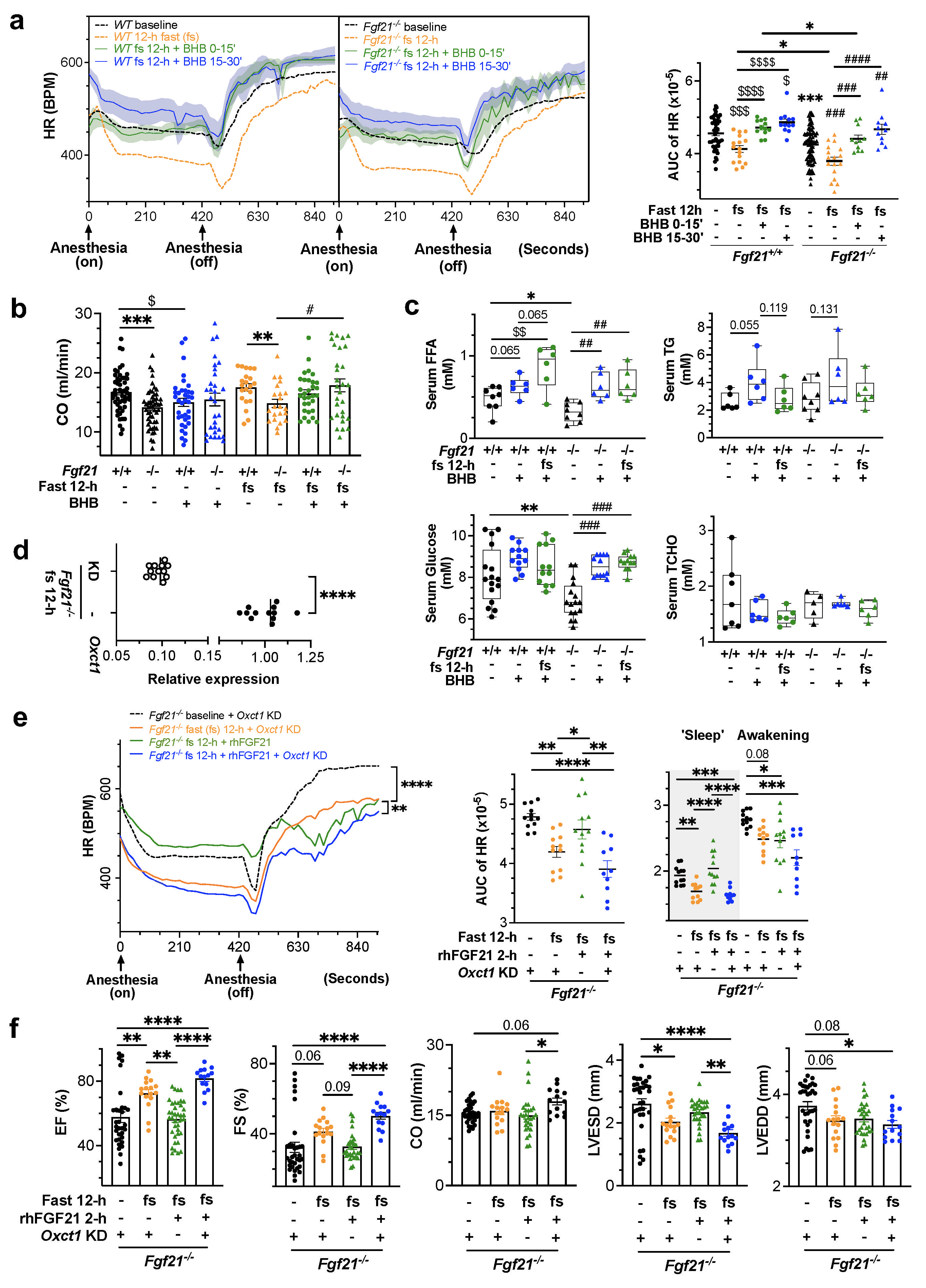
**

**Fig. S20. Roles of FGF21-regulated cardiac ketolysis in heart function performance during fast.**

**Related to Fig. 5f-5i.**

(**a**) More detailed comparisons for the effects of BHB supplementation (10 mmol 1,3-butanediol monoester per mouse) on HR in FGF21-deficient vs WT mice, under basal and 12-h fast (fs) conditions (n=10-12 for each group and each condition). See Fig. 5f. Note that the FGF21-regulated cardiac ketolysis appeared to occur during both 12-h and 24-h fasts (Fig. 5e, S19b-S19e), although it was attenuated during the prolonged fast. Thus, we chose the 12-h fast condition to assess BHB’s effect on heart energetic function. However, it would be interesting to know where the ketone body is produced during the 12-h fast, as hepatic ketogenesis occurred only during the prolonged fast (Fig. 6b and S25b-S25d)

(**b**) Effects of BHB supplementation on cardiac output in FGF21-deficient vs WT mice, under both basal and 12-h fast conditions.

(**c**) Effects of BHB supplementation on serum FFA, TG, glucose and total cholesterol (Tcho) levels in *Fgf21^-/-^* vs WT mice, under both basal and 12-h fast conditions. See Fig. 5h.

(**d**) Deficiency of cardiac *Oxct1* expression by pAAV9-cTnT-EGFP-shRNA(*mOxct1*)-WPRE mediated knockdown.

(**e**) More detailed comparisons for the effects of cardiac *Oxct*1 knockdown on HR in FGF21-deficient mice, under basal, 12-h fast, and acute rhFGF21 treatment conditions (n=10-12 for each group and each condition). See Fig. 5i.

(**f**) Effects of cardiac *Oxct*1 knockdown on Echo parameters EF, FS, CO, LVESD, and LVEDD in FGF21-deficient mice, under basal, 12-h fast, and acute rhFGF21 treatment conditions (n=10-12 for each group and each condition).

**Figure S21.**

**
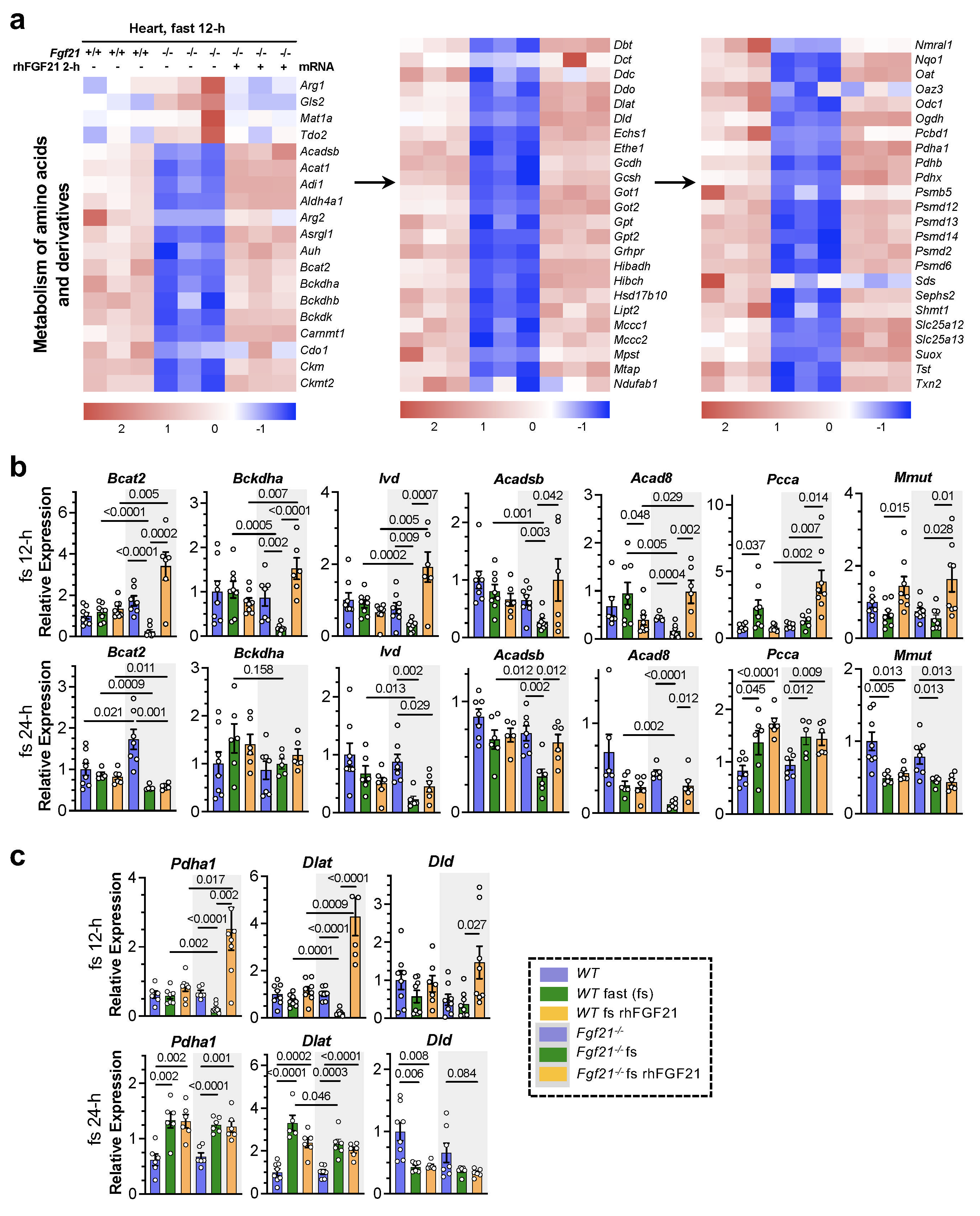
**

**Fig. S21. Roles of FGF21-regulated cardiac BCAA catabolism in heart function performance during fast.**

**Related to Fig. 5j-5k.**

(**a**) Transcriptomic enrichment heatmap for the expression of cardiac genes involved in metabolism of amino acids and derivatives, due to FGF21 deficit and restoration in the indicated mouse groups under a 12-h fast.

(**b**-**c**) qRT-PCR analysis for the expression of cardiac genes involved in BCAA catabolism in *Fgf21*-null vs WT mice under basal, 12-h vs 24-h fast (fs), and then 2-h rhFGF21 treatment conditions.

**Figure S22.**

**
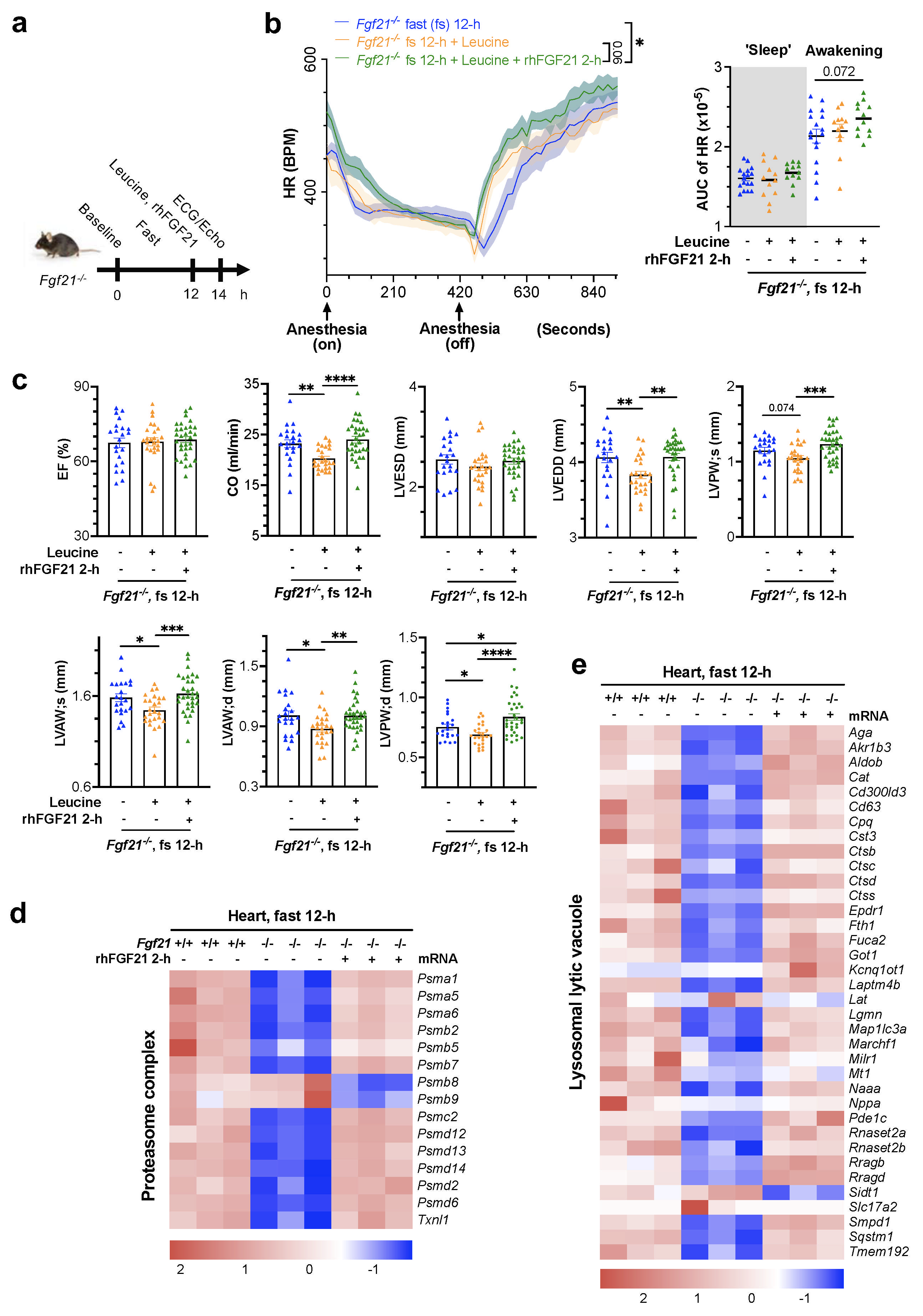
**

**Fig. S22. Effects of leucine supplementation and roles of proteasome complex and lysosomal lytic vacuole in FGF21-regulated heart energetic function during fast.**

**Related to Fig. 5j-5k.**

(**a**) Experimental scheme for assessing leucine supplementation (0.5 mg per mouse) effects on heart energetic efficiency in *Fgf21*-null mice under a 12-h fast and then acute rhFGF21 treatment.

(**b**) Effects of leucine supplementation on HR.

(**c**) Effects of leucine supplementation on Echo parameters.

(**d**) Transcriptomic heatmap for changes in cardiac proteasome complex due to FGF21 deficit and restoration in the indicated mouse groups under a 12-h fast.

(**e**) Transcriptomic heatmap for changes in cardiac lysosomal lytic activities.

**Figure S23.**

**
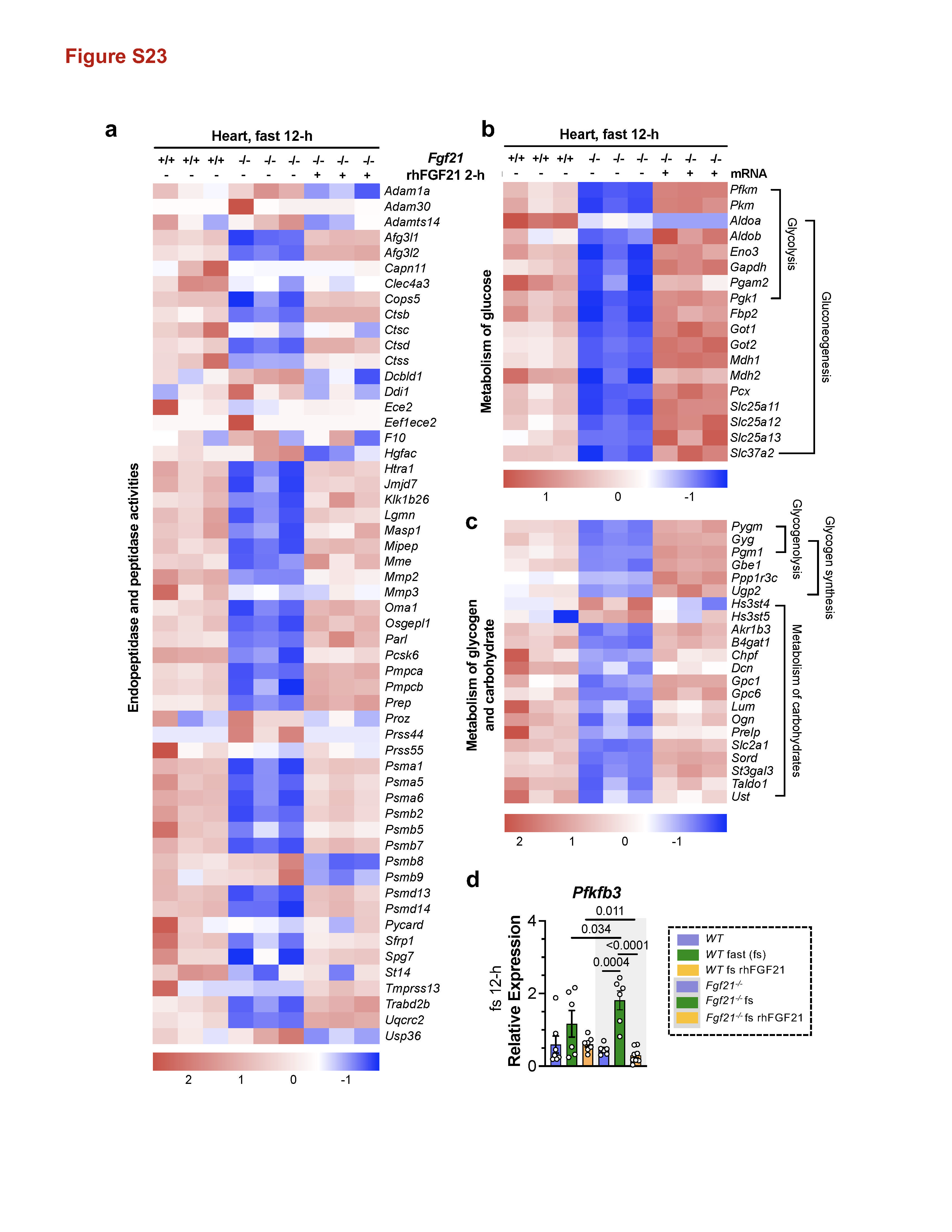
**

**Fig. S23. Roles of FGF21 in regulating cardiac glucose/glycogen metabolism during fast.**

**Results related to cardiac glucose and nucleotide flux in association with heart energetic performance (**see **Fig. 5** and **S24):**

Carbohydrates and glycogen are also vital cardiac fuels. Under 12-h fasting, FGF21-deficient mouse hearts exhibited impaired glucose/glycogen flux pathways. Conversely, rhFGF21 normalized/enhanced these pathways (Fig. S23b-S23d). *Pfkfb3*, which regulates F-2,6-BP levels (a glycolysis accelerator via F-1,6-BP), was upregulated by FGF21’s loss and downregulated by rhFGF21 under fasting. The expression of gluconeogenic *Fbp2* and glycolytic *Pfkm* and *Aldoa*, which directly control F-1,6-BP levels, showed significant changes, which reflect cardiac F-1,6-BP and VCO_2_/VO_2_ alterations (Fig. 3j and 4a). Thus, FGF21 loss causes a glucose hypometabolic state with relatively enhanced glycolysis while FGF21 signaling promotes cardiac glucose/glycogen flux. Similarly, FGF21 loss exclusively downregulated, while rhFGF21 upregulated, nucleotide flux (anabolism and catabolism), particularly purine nucleosides, essential for mitochondrial DNA synthesis, translation, turnover, and energy conversion (Fig. S24a-S24c). These changes correlated with serum adenosine, AICAR, hypoxanthine, and cardiac AMP, ATP, and NADH levels. Altogether, findings from serum and cardiac metabolomes, pathway metabolons, and transcriptomes suggest that FGF21 loss leads to a hypometabolic/hypo-energy state in the heart, while FGF21 signaling promotes the flux of various metabolic substrates, ensuring cardiac metabolic sufficiency, fuel flexibility, and thus function efficiency. This mechanism is crucial for preventing cardiac stress vulnerability and maintaining energetic performance.

(**a**) Transcriptomic heatmap for changes in cardiac endopeptidases and peptidases.

(**b**) Transcriptomic heatmap for pathway changes in glycolysis and gluconeogenesis due to FGF21 deficit and restoration in the indicated mouse groups under a 12-h fast.

(**c**) Transcriptomic heatmap for pathway changes in glycogenolysis, glycogenesis and carbohydrate metabolism.

(**d**) qRT-PCR analysis for the expression of cardiac gene *Pfkfb3* involved in glycolysis in *Fgf21*-null vs WT mice, under basal, 12-h fast (fs), and then 2-h rhFGF21 treatment conditions.

**Figure S24.**

**
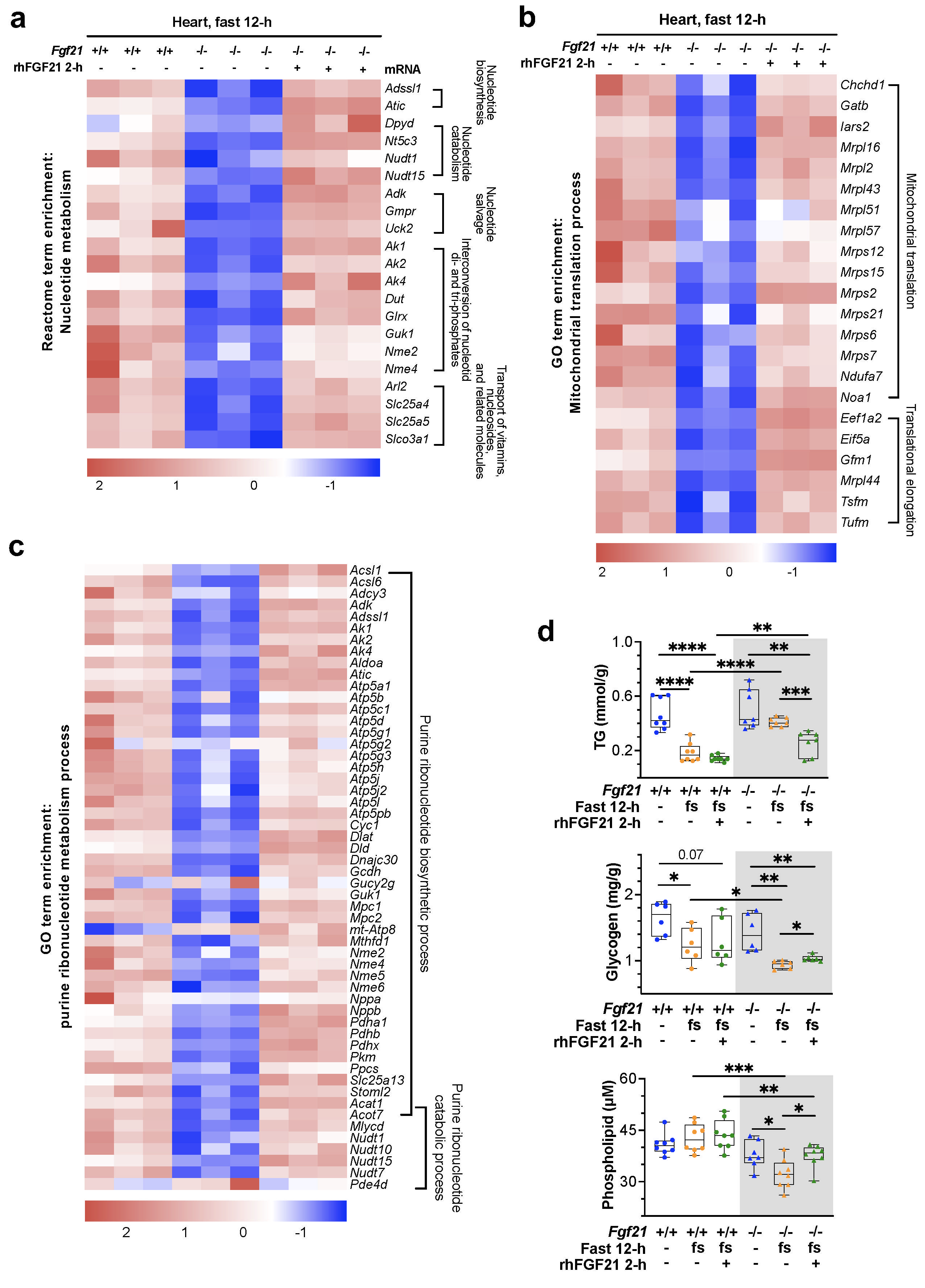
**

**Fig. S24. Roles of FGF21 in regulating cardiac nucleotide metabolism and mitochondrial translation during fast.**

**Related to Fig. 5.**

(**a**) Transcriptomic heatmap (Reactome term enrichment) for changes in cardiac nucleotide biosynthesis, catabolism, interconversion, and transport due to FGF21 deficit and restoration in the indicated mouse groups under a 12-h fast.

(**b**) Transcriptomic heatmap (GO term enrichment) for changes in cardiac mitochondrial translation and elongation.

(**c**) Transcriptomic heatmap (GO term enrichment) for changes in cardiac purine ribonucleotide biosynthesis and catabolism.

(**d**) Changes in cardiac contents of triglycerides, glycogen, and phospholipids in *Fgf21*-null mice during a 12-h fasting, compared to the wildtype mice, rhFGF21 restoration and under basal conditions as indicated. n=6-8 per group.

**Figure S25.**

**
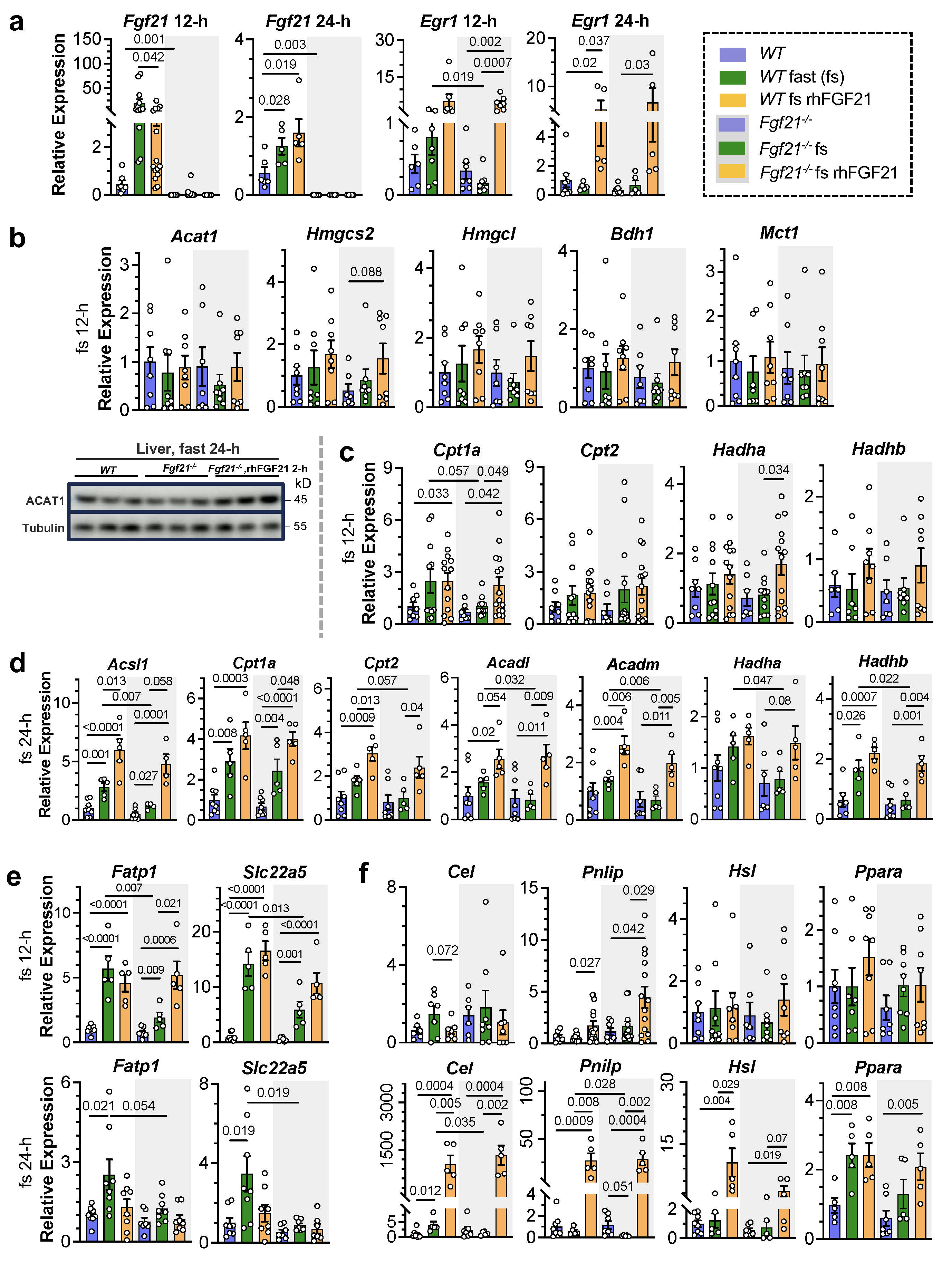
**

**Fig. S25. Roles of FGF21 in regulating hepatic ketogenesis in association with heart energy flux during fast.**

**Related to Fig. 6a-6d.**

(**a**) Detailed qRT-PCR analysis for the expression of hepatic *Fgf21* and *Egr1* in *Fgf21*-null vs WT mice under basal, 12-h vs 24-h fast (fs), and then 2-h rhFGF21 treatment conditions.

(**b**) Upper, qRT-PCR analysis for the expression of hepatic genes involved in ketogenesis pathway in *Fgf21*-null vs WT mice under basal, 12-h fast, and then 2-h rhFGF21 treatment conditions. n=7-8 per group. Lower right, Western blot analysis confirms transcriptomic and qPCR data for the expression of hepatic *Acat1* gene (as an example) as indicated. n=3 per group. For 24-h fast effects, see Fig. 6b.

(**c**) qRT-PCR analysis for the expression of hepatic genes involved in ketogenesis-associated FAO pathway in the indicated groups as in b following 12-h fast.

(**d**) qRT-PCR analysis of hepatic FAO pathway upstream of ketogenesis in *Fgf21^-/-^* (n=4-5) vs WT mice (n=5) under conditions as in b following 24-h fast.

(**e**-**f**) qRT-PCR analysis for the expression of hepatic genes involved in hepatic FFA, carnitine import, and lipolysis in *Fgf21*-null vs WT mice under basal, 12-h vs 24-h fast, and then 2-h rhFGF21 treatment conditions.

**Figure S26.**

**
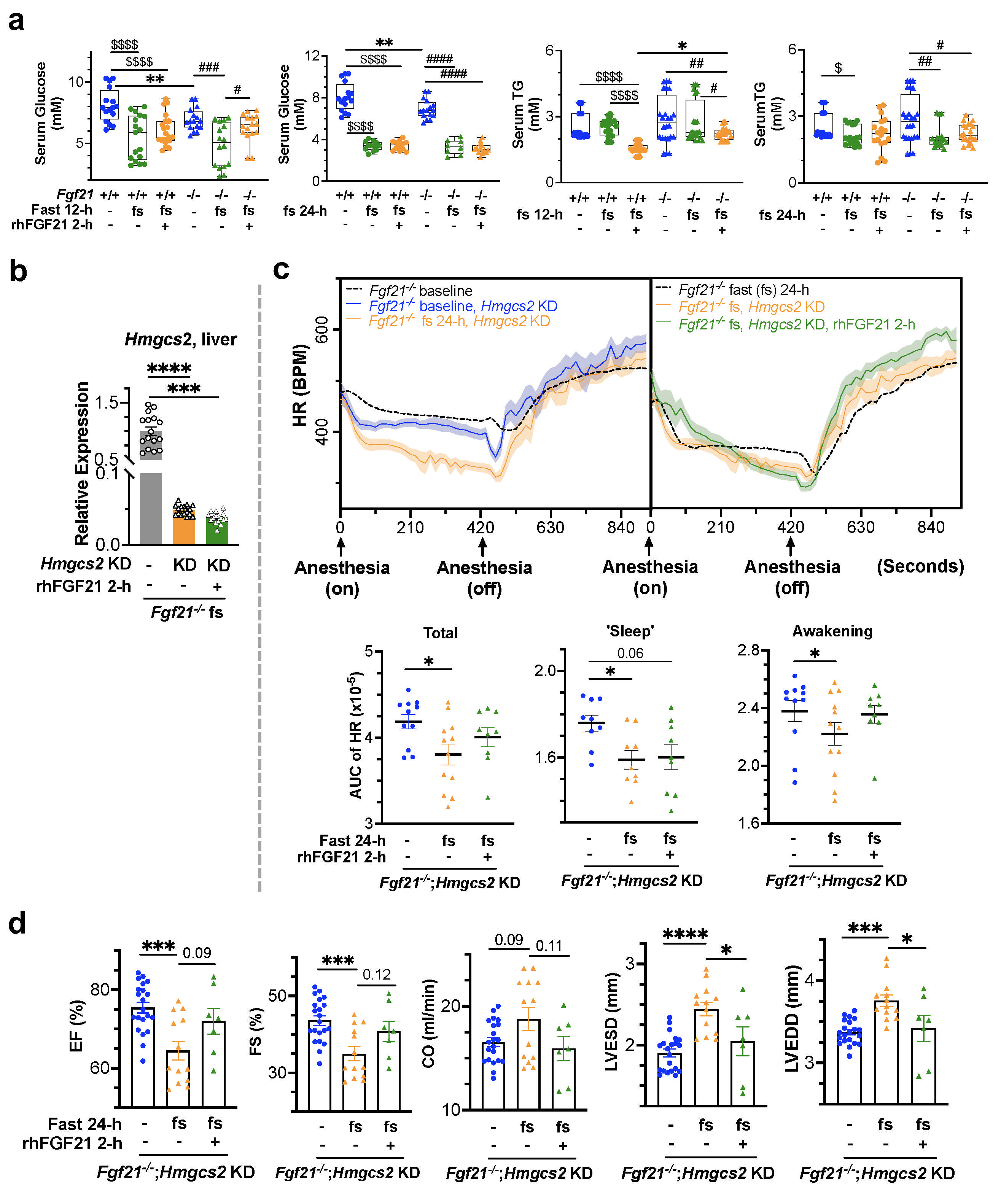
**

**Fig. S26. Effects of defective hepatic ketogenesis on FGF21-mediated improvements of cardiac energetic efficiency during prolonged fast.**

**Related to Fig. 6c-6d.**

(**a**) Roles of FGF21 in regulating serum glucose and TG, which provide substrate flux to intracardiac mitochondria via the transcardiac route (crossing the blood barrier into the interfibrillar/interstitial spaces and cardiomyocytes), in *Fgf21*-null vs WT mice under basal, 12-h vs 24-h fast, and then 2-h rhFGF21 treatment conditions. For effects on serum BHB and FFA, see Fig. 6c.

(**b**) Knockdown (KD) efficiency of hepatic *Hmgcs2* expression in *Fgf21*-null mice by AAV9-mediated shRNA, analyzed by qRT-PCR.

(**c**) More detailed comparisons for the effects of hepatic HMGCS2 deficit on HR in FGF21-deficient mice, under basal, 24-h fast (fs), and acute rhFGF21 treatment conditions (n=6-9 as indicated). See Fig. 6d.

(**d**) Effects of hepatic HMGCS2 deficit on FGF21-mediated improvements of Echo parameters EF, FS, CO, LVESD, and LVEDD in the indicated groups.

**Figure S27.**

**
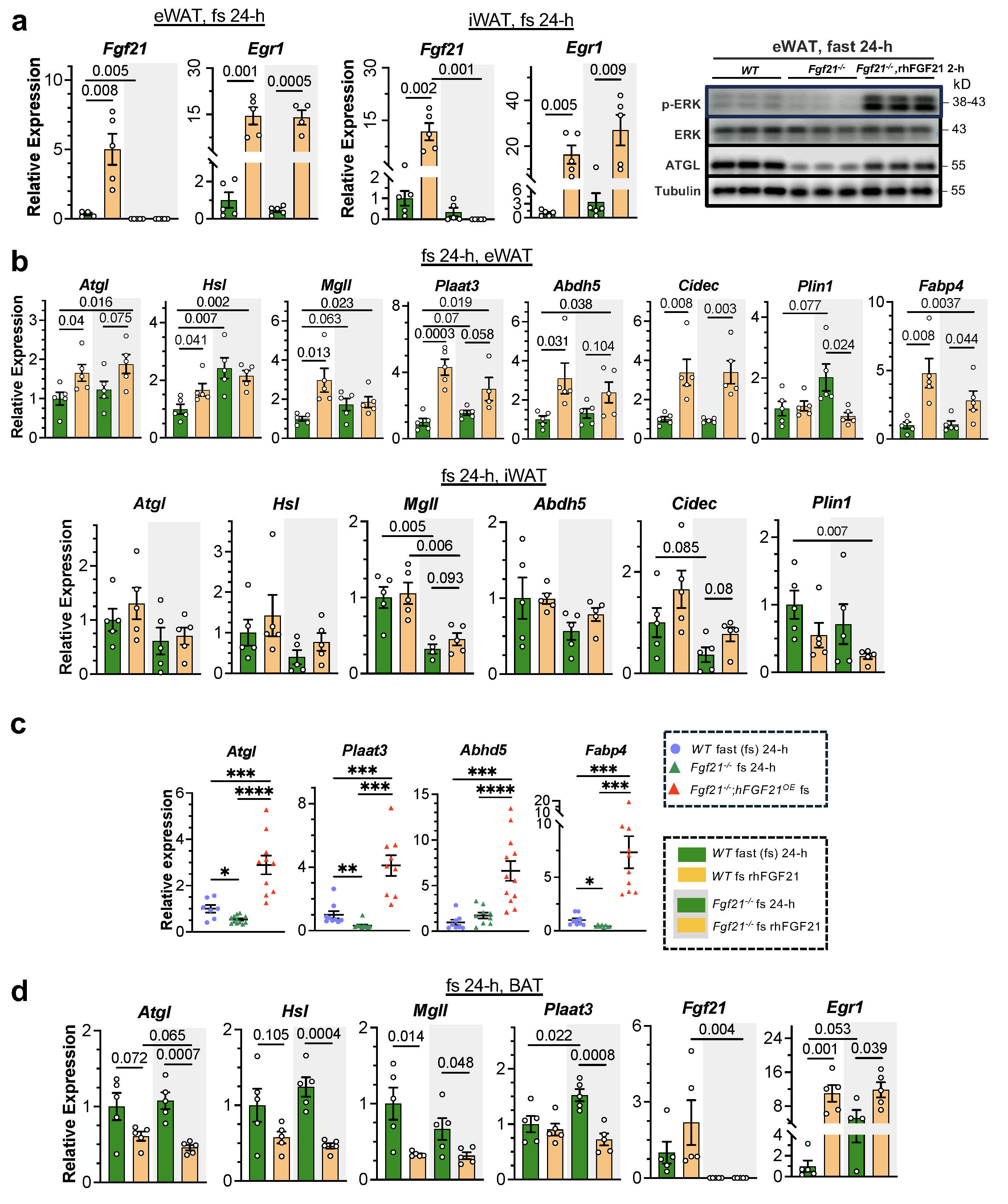
**

**Fig. S27. Roles of FGF21 in white adipose tissue lipolysis during prolonged fast.**

**Related to Fig. 6f-6g.**

**Results related to adipose tissue lipolysis in association with heart energetic performance (**see **Fig. 6f-6g):**

WAT is the primary FFA source during prolonged fast/starvation via lipolysis, which meets the temporal pattern of hepatic ketogenesis and serum BHB levels 24 hours post-fast (Fig. S25f, 6b-6c).^6^ After 24-h fast, both fat depots (eWAT and iWAT) responded significantly to rhFGF21 (Fig. S27a). FGF21 deficiency trended to elevate lipases for TG (ATGL, HSL and MGLL) and phospholipid (PLAAT3) in eWAT but slightly reduce them in iWAT (Fig. S27b-S27c). Levels of lipolysis-promoting accessory proteins (ABHD5, CIDEC) and FFA exporter (FABP4) followed a similar trend, while the lipogenic PLIN1 showed opposite changes. Acute rhFGF21 treatment or FGF21 overexpression upregulated lipolysis, consistent with other reports.^6,69^ Serum FFA levels during fast mirrored lipolytic changes but were used for BHB production upon rhFGF21 treatment (Fig. 6c). These data suggest that FGF21 promotes WAT lipolysis to meet hepatic ketogenesis under prolonged fasting. Interestingly, during 24-h fast, FGF21 loss minimally affected lipolysis in BAT, but rhFGF21 significantly downregulated it (Fig. S27d). This suggests that under thermoneutral but fasting conditions, non-shivering thermogenesis is suppressed to conserve lipid energy for other organs (e.g., liver, heart), which is regulated by FGF21.

(**a**) qRT-PCR analysis for the expression of *Fgf21* and *Egr1* in eWAT and iWAT in *Fgf21*-null vs WT mice under 24-h fast (fs) and then 2-h rhFGF21 treatment conditions. n=5-6 per group. Right, Western blot analysis confirms qPCR data for the expression of *Atgl* genes and the activation of FGF21 downstream MAPK pERK1/2 in eWAT as indicated. n=3 per group.

(**b**) Upper: Detailed qRT-PCR analysis of gene expression involved in eWAT lipolysis in *Fgf21^-/-^* (n=5) vs WT mice (n=5) under 24-h fast and then 2-h rhFGF21 treatment. Lower: qRT-PCR analysis for the expression of lipolytic genes in iWAT.

(**c**) qRT-PCR analysis for the expression of lipolytic genes in eWAT in FGF21-restored (*hFGF21^OE^*) *Fgf21*-null mice under 24-h fast. *hFGF21^OE^*, AAV9-mediated overexpression of human *FGF21* (hFGF21) via a AAV9-TBG-*hFGF21*-HA-IRES-EGFP mini-gene.

(**d**) qRT-PCR analysis for the expression of lipolytic genes in BAT in *Fgf21*-null vs WT mice under 24-h fast and then 2-h rhFGF21 treatment conditions.

**Figure S28.**

**
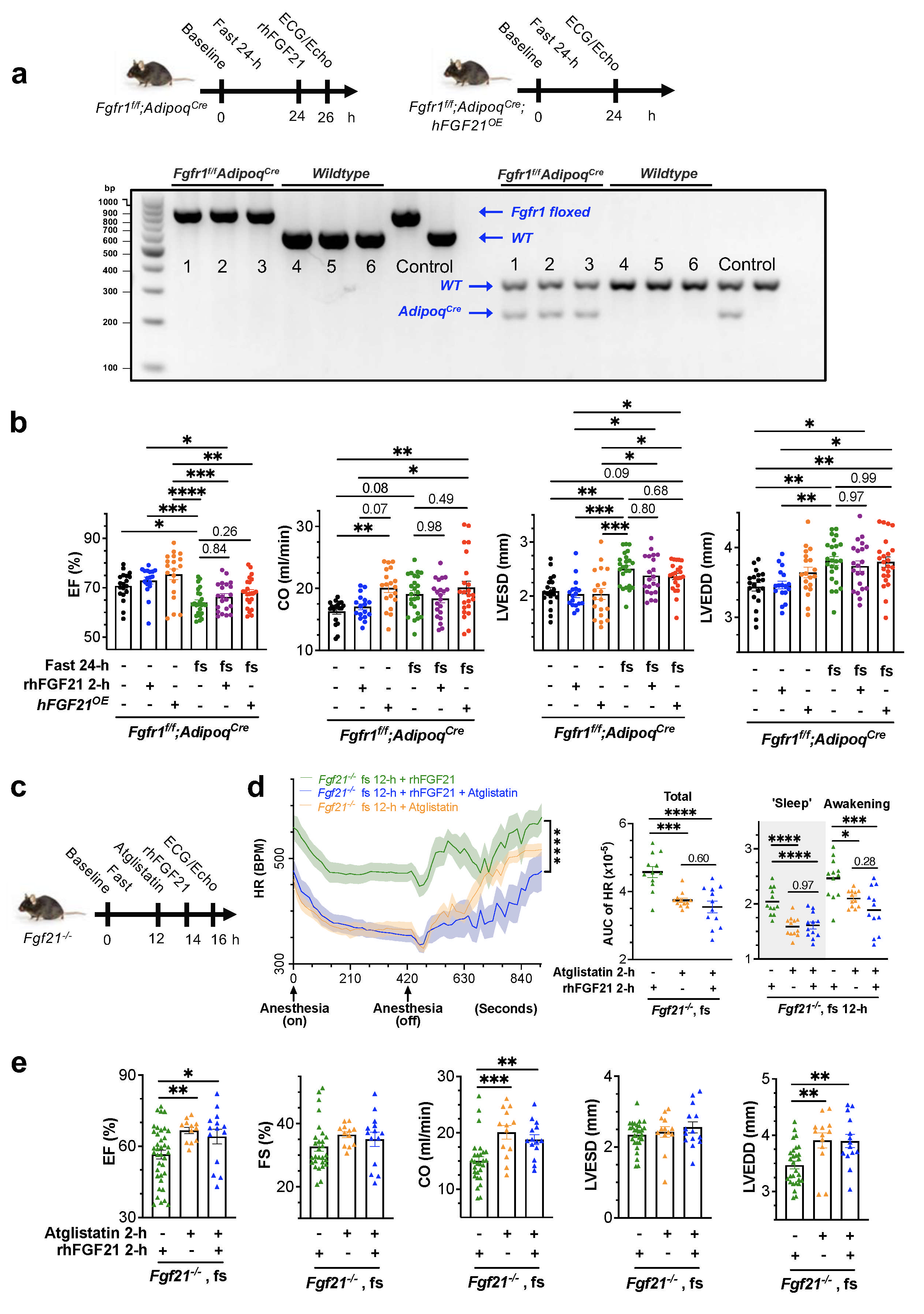
**

**Fig. S28. Effects of defective lipolysis in white adipose tissues and systemically on FGF21-mediated improvements of cardiac energetic efficiency during prolonged fast.**

**Related to Figure 6f-6i.**

(**a**) Experimental scheme for assessing the contribution of FFA flux of white adipose tissues to heart energetic performance during prolonged fast. Left, scheme for acute rhFGF21 (1 mg/Kg body weight) treatment in mice with WAT-specific deletion of FGFR1 (*Fgfr1^f/f^AdipoQ^Cre^*). Right, scheme for overexpression of hFGF21 in *Fgfr1^f/f^AdipoQ^Cre^* mice. Lower panel, genotyping, n=3 per group.

(**b**) Effects of adipose FGFR1 deficit on FGF21-mediated improvements of Echo parameters EF, FS, CO, LVESD, and LVEDD in the indicated groups.

(**c**) Experimental scheme for assessing the contribution of cardiac lipolysis to FGF21-regulated heart energetic performance during early fast. Atglistatin at 1.42 mg/mouse (*i.p*) is used to inhibit ATGL. Note that our results revealed that the heart utilizes FFAs during both 12-h and 24-h fasts (fs), which may come from the circulating (blood and interstitial) FFAs and from cardiomyocytic lipolysis via ATGL, but not WAT lipolysis yet (which occurs during prolonged or 24-h fast under FGF21 regulation). This experiment is thus designed to assess FGF21-mediated effects of cardiac lipolysis or cardiac FFA flux on heart function.

(**d**) More detailed comparisons for the effects of ATGL inhibition during early fast on HR in FGF21-deficient mice with acute rhFGF21 treatment (1 mg/Kg body weight). n=10-12 for each group and each condition. See Fig. 6h.

(**e**) Effects of ATGL inhibition during early fast on FGF21-mediated improvements of Echo parameters EF, FS, CO, LVESD, and LVEDD in the indicated groups.

**Figure S29.**

**
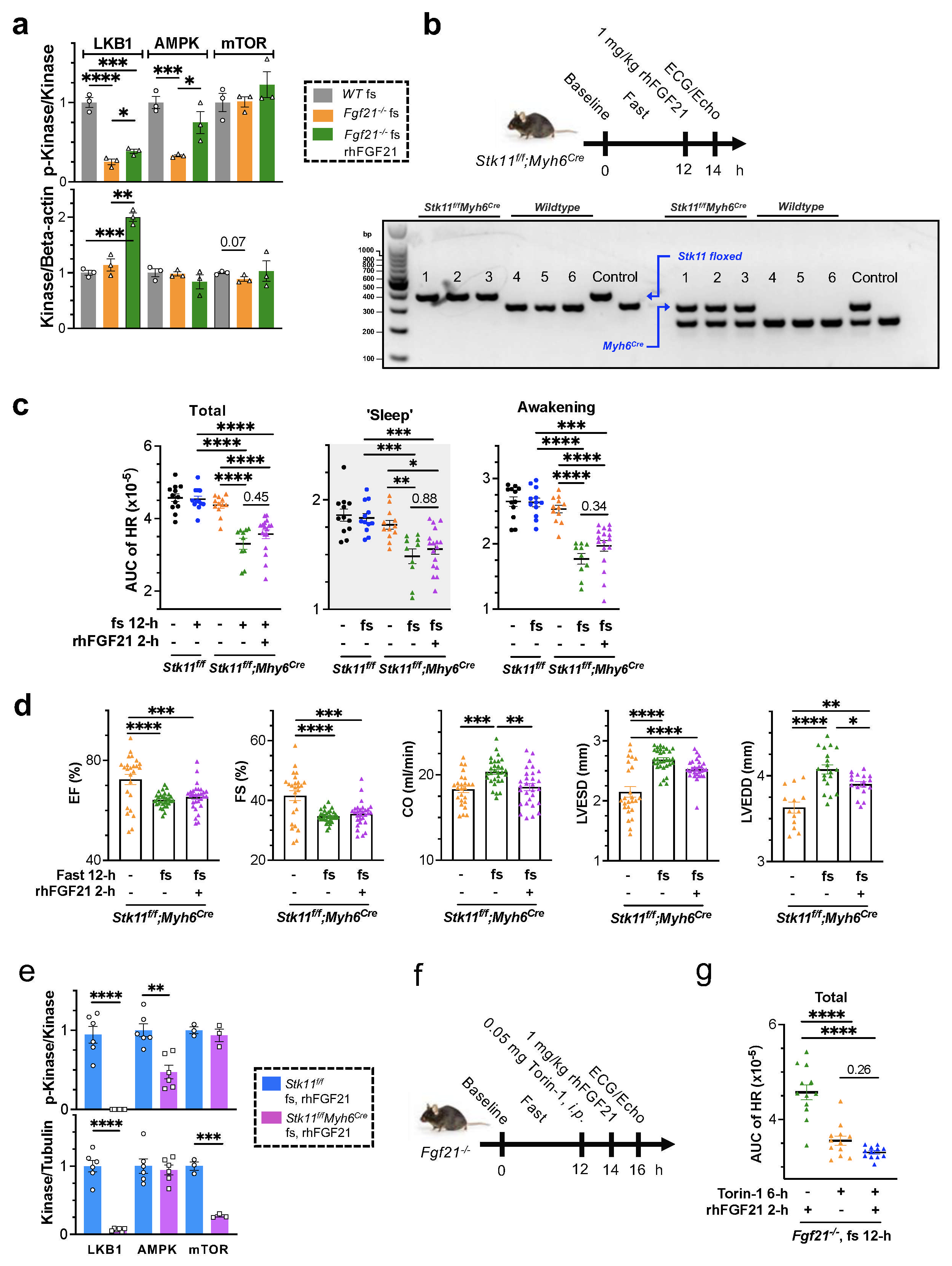
**

**Fig. S29. Roles of LKB1-AMPK-mTOR energy and stress pathway in FGF21-mediated regulation of cardiac energetic efficiency during fast.**

**Related to Fig. 7.**

(**a**) Changes in kinase phosphorylation and total kinase protein of LKB1, AMPK and mTOR by Western Blotting analysis as indicated, n=3-6 for each group. See Fig. 7b.

(**b**) Experimental scheme for assessing the contribution of cardiac LKB1 to FGF21-regulated heart energetic performance during fast, using a mouse model with cardiac specific knockout of *Stk11* (*Stk11^f/f^Myh6^Cre^*). Lower panel, genotyping, n=3 per group.

(**c**) AUC (total, sleep and awakening phases) analysis of HR excursion curves as indicated in Fig. 7c.

(**d**) Effects of cardiac LKB1 deficiency on FGF21-mediated improvements of Echo parameters EF, FS, CO, LVESD, and LVEDD during fast in the indicated groups.

(**e**) Changes in kinase phosphorylation and total kinase protein of LKB1, AMPK and mTOR by Western Blotting analysis as indicated, n=3-6 for each group. See Fig. 7d.

(**f**) Experimental scheme for assessing the contribution of cardiac mTOR to FGF21-regulated heart energetic performance during fast, using mTOR inhibitor Torin-1.

(**g**) AUC (total) analysis of HR excursion curves as indicated in Fig. 7e.

**Figure S30.**

**
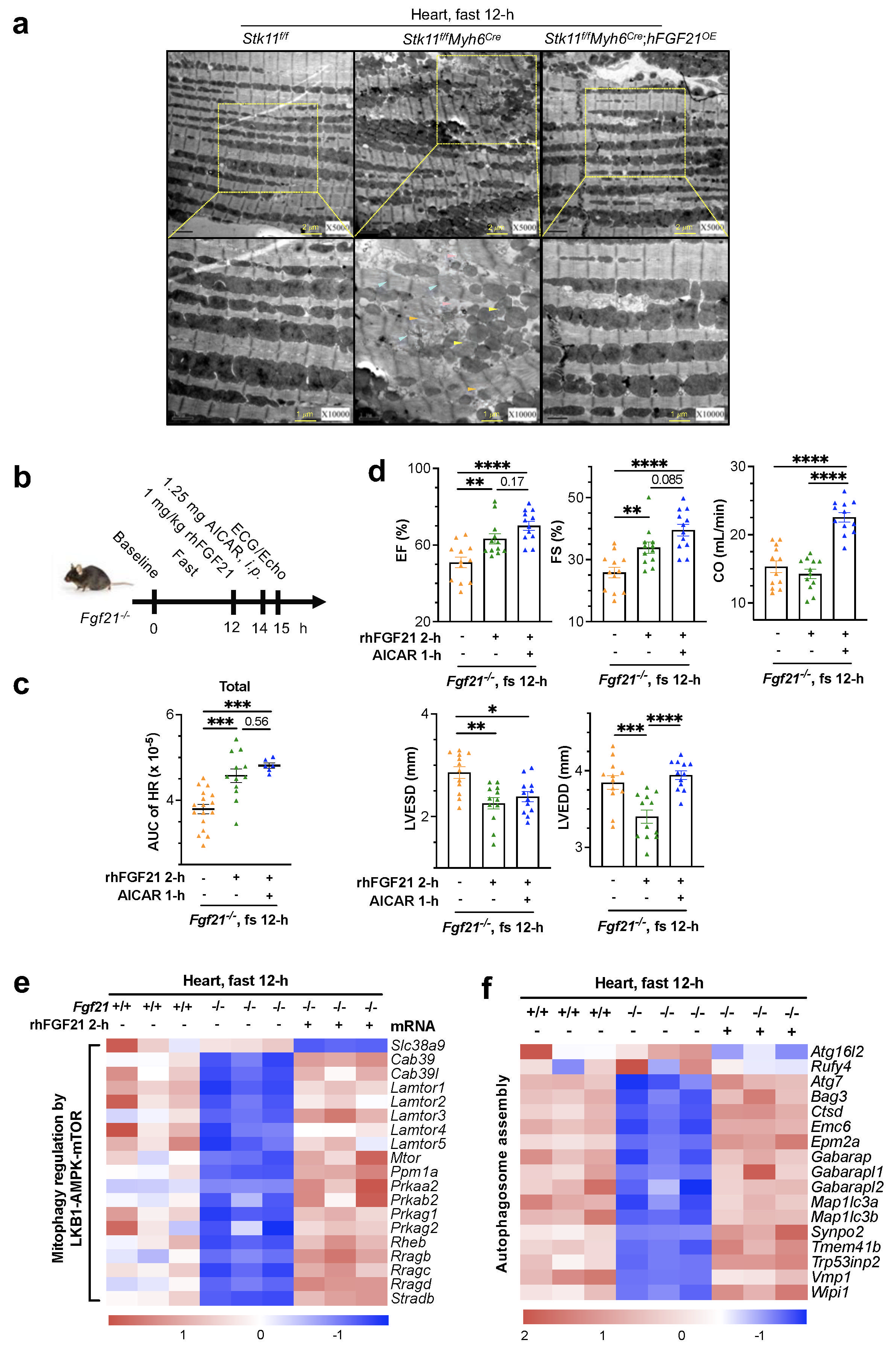
**

**Fig. S30. Roles of LKB1-AMPK-mTOR energy and stress pathway in FGF21-mediated regulation of cardiac energetic efficiency during fast.**

**Related to Fig. 7.**

(**a**) Chronic FGF21 signaling mitigates myocardial and mitochondrial structural defects caused by LKB1 deficiency. Transmission electron microscopy was employed to examine heart sections from *Stk11^f/f^Myh6^Cre^* mice with or without AAV-mediated hFGF21 overexpression, compared to the *Stk11^f/f^* control mice. All mice were fasted for 12 hours prior to analysis. Yellow arrowhead, mitochondrial vacuolation. Cyan arrowhead, Z-line distortion. Light-pink arrowhead, muscle fiber loss or fibrotic displacement. Orange arrowhead, myofibrillar disorganization. Images are representative of n=15 per group.

(**b**) Experimental scheme for assessing the contribution of cardiac AMPK to FGF21-regulated heart energetic performance during fast, using AMPK activator AICAR.

(**c**) AUC (total) analysis of HR excursion curves as indicated in Fig. 7h.

(**d**) Effects of AMPK activation on FGF21-mediated improvements of Echo parameters EF, FS, CO, LVESD, and LVEDD during fast in the indicated groups.

(**e**) Transcriptomic heatmap for LKB1-AMPK-mTOR pathways involved in mitophagy and macroautophagy in the indicated mouse groups. See Fig. 7a and 7i.

(**f**) Transcriptomic enrichment heatmap for autophagosome assembly process in the indicated mouse groups.

**Table S1. Changes in serum metabolites in FGF21-deficient mice under normal dietary and resting conditions.**

Blood samples were collected from *Fgf21^-/-^* and wildtype (WT) mice at 8 weeks of age (n=3 per group) and subjected to untargeted metabolomic analysis as described in the Materials and Methods section. The table shows changes in the levels of serum metabolites across the indicated groups and conditions detected by both negative and positive ion modes. For a complete list of detected metabolites, please refer to MetaboLights with access number MTBLS11726.

See a separate file for the Table S1.

**Table S2. Changes in cardiac energy metabolism-associated metabolites in FGF21-deficient mice under fasting and FGF21 treatment.**

Heart samples were collected from *Fgf21^-/-^* mice, both with and without acute rhFGF21 treatment, and from wildtype (WT) mice at 8 weeks of age (n=3 per group) following a 12-h fast. These samples were subjected to targeted energy metabolomic analysis, as detailed in the Materials and Methods section. Changes in the levels of metabolites across the indicated groups and conditions are presented. For further information, please refer to MetaboLights with access number MTBLS11725.

See a separate file for the Table S2.

**Table S3. Cardiac transcriptomic changes** **in FGF21-deficient mice under fasting and FGF21 treatment.**

Heart samples were collected from *Fgf21^-/-^* mice, both with and without acute rhFGF21 treatment, and from wildtype (WT) mice at 8 weeks of age (n=3 per group) following a 12-h fast. These samples were processed for RNA-Seq analysis, as detailed in the Materials and Methods section. Changes in the expression levels of genes across the indicated groups and conditions are presented. For further information, please refer to Sequence Read Archive (SRA) with access number PRJNA1189445.

See a separate file for the Table S3.

**Table S4: Primer sequences for mouse genotyping**

| Genotype | Species | Primers sequences |
| --- | --- | --- |
| *Fgf21^-/-^* | Mouse | F1: 5′-GACTGTTCAGTCAGGGATTG-3′  F2: 5′-CCCGTGATATTGCTGAAGAG-3′  R1: 5′-ACAGGGTCTCAGGTTCAAAG-3′ |
| *Fgfr1^flox/flox^* | Mouse | F1: 5′-GGACTGGGATAGCAAGTCTCTA-3′  R1: 5′-GTGGATCTCTGTGAGCCTGAG-3′ |
| *Stk11^flox/flox^* | Mouse | F1: 5′-GGTATCAGACCTGAGCTAAGGGAA-3′  R1: 5′-GGTTTCAGGCCATAGTGGAGAAG-3′ |
| *Myh6*-Cre | Mouse | F1: 5′-TCTATTGCACACAGCAATCCA-3′  R1: 5′-CCAGCATTGTGAGAACAAGG-3′ |
| *AdipoQ*-Cre | Mouse | F1: 5′-ACGGACAGAAGCATTTTCCA-3′  R1: 5′-GGATGTGCCATGTGAGTCTG-3′  F2: 5′-CTAGGCCACAGAATTGAAAGATC-3′  R2: 5′-GTAGGTGGAAATTCTAGCATCATCC-3′ |

**Table S5: Primer sequences for qRT-PCR**

| Genes | Species | Primers sequences |
| --- | --- | --- |
| *Fgf21* | Mouse | F: 5′-GGGCACCGGAGTCAGAACAC-3′  R: 5′-ACAGTCCCAGGGTCCCAACT-3′ |
| *Hprt1* | Mouse | F: 5′-CAGTCCCAGCGTCGTGATTA-3′  R: 5′-TGGCCTCCCATCTCCTTCAT-3′ |
| *Egr1* | Mouse | F: 5′-TGTGGGAGGAAGTTTGCCAG-3′  R: 5′-GGATGGGTAGGAGGTAGCCA-3′ |
| *Mct1* | Mouse | F: 5′-AGTGCAACGACCAGTGAAGT-3′  R: 5′-GCGATCATTACTGGACGGCT-3′ |
| *Mct2* | Mouse | F: 5′-CATTCAACCTGCAACCAGCC-3′  R: 5′-ACTCCCCTTCCAGCCATAGT-3′ |
| *Bdh1* | Mouse | F: 5′-CTCGCCATACTGCATCACCA-3′  R: 5′-CACACTGACCTTGACACCCA-3′ |
| *Bdh2* | Mouse | F: 5′-GAACGGTTGACACCCCATCT-3′  R: 5′-GTATACGCAGAGCAGAGCGA-3′ |
| *Oxct1* | Mouse | F: 5′-CTGGAGTTTGAGGACGGCAT-3′  R: 5′-TCCGCATCAGCTTCGTCTTT-3′ |
| *Acat1* | Mouse | F: 5′-AATGCCAGCACACTGAACGA-3′  R: 5′-ATCAATGGGGTCTACGGCAG-3′ |
| *Suclg1* | Mouse | F: 5′-GTCTTACACAGCCTCTCGGAAAC-3′  R: 5′-ACTCCAAAGCCTGCTGACTGTG-3′ |
| *Suclg2* | Mouse | F: 5′-TAGCGGTTTGAAAGGAGGTGT-3′  R: 5′-TTGGAGTTTGTTTCGTTGCTAG-3′ |
| *Sucla2* | Mouse | F: 5′-CTGACGGAAAGGTGTTGTGC-3′  R: 5′-CTTCTTCTGGCGGTAGGCTG-3′ |
| *Cs* | Mouse | F: 5′-CTTGGGAGCCAAGAACTCATC-3′  R: 5′- TTCATGCCTCTCATGCCACC-3′ |
| *Aacs* | Mouse | F: 5′-CAGATGTCCCCGAGTGGTTC-3′  R: 5′- CACGTAAAGGGCGACTCTGT-3′ |
| *Cpt1b* | Mouse | F: 5′-TGGCATCCTTAGGGGTGTGTA-3′  R: 5′-GGGTCCCAAAGTGGCCATAC-3′ |
| *Cpt2* | Mouse | F: 5′-CCCAAACCCAGTCGTGATGA-3′  R: 5′-TGTGCCTGGATTTCTGAGGG-3′ |
| *Acox1* | Mouse | F: 5′-GACAGAGATGGGTCATGGAACT-3′  R: 5′-CATGTAACCCGTAGCACTCCC-3′ |
| *Hadha* | Mouse | F: 5′-GCACAGCACGTAGCAGAAGA-3′  R: 5′-GCGACCTAAGAAGCCCTTGG-3′ |
| *Hadhb* | Mouse | F: 5′-GGCGCTTCTGCGATGCTAAT-3′  R: 5′-GGAGTAGCATATGTTGGTCCGAGT-3′ |
| *Acadl* | Mouse | F: 5′-CATTGGTGGGGACTTGCTCT-3′  R: 5′-TGGCTATGGCACCGATACAC-3′ |
| *Acadm* | Mouse | F: 5′-AAAAGAGCCTGGGAACTCGG-3′  R: 5′-CCATACGCCAACTCTTCGGT-3′ |
| *Ehhadh* | Mouse | F: 5′-TCCTCGGTTGGTGTTCTTGG-3′  R: 5′-AAGCTTGGCCACTCTTCGAT-3′ |
| *Hsd17b4* | Mouse | F: 5′-GGCTCTGGCGTAGTGATTGT-3′  R: 5′-GTCCGTTTTCCACCAAAGCC-3′ |
| *Bcat2* | Mouse | F: 5′-GGCTCCTACTTCCCTGGAGA-3′  R: 5′-CAGCCACAGTGGGTCCATAG-3′ |
| *Bckdha* | Mouse | F: 5′-GACCAACTATGGCGAGGAGG-3′  R: 5′-GTCATTCACGTTGCCGTAGC-3′ |
| *Dbt* | Mouse | F: 5′-GCCAAGCCAGTGATATTGCC-3′  R: 5′-CCTTTCTGGTCAAATCGGGGA-3′ |
| *Mmut* | Mouse | F: 5′-AGAGCACACTGCCAGACATC-3′  R: 5′-CCATGGCTTCGATTGCAGTG-3′ |
| *Mcee* | Mouse | F: 5′-AATCCAGTCCTGTGTGGAAGC-3′  R: 5′-ACTCCATGTTCCGGCAGAG-3′ |
| *Pcca* | Mouse | F: 5′-CCTCAGAAGAGGCAAAACCGA-3′  R: 5′-GCCTGTCGCCTCTGTGTATATT-3′ |
| *Pccb* | Mouse | F: 5′-CTCAGCGTTCTGAATTGCGG-3′  R: 5′-AGAAGACTGATCCGCTCCCT-3′ |
| *Acss1* | Mouse | F: 5′-GGCTGACATCGGTTGGATCA-3′  R: 5′-CAGGGTAAACTGGGGTGCTC-3′ |
| *Acss2* | Mouse | F: 5′-GCCATATGCTGACCCCTCTC-3′  R: 5′-TCCCCGGACTCATTCAGGAT-3′ |
| *Ogdh* | Mouse | F: 5′-GGCCACAATGAGATGGACGA-3′  R: 5′-TCAGGCTGATTGACGACACC-3′ |
| *Dlst* | Mouse | F: 5′-TGCCTGGGGTCTCCTTATGT-3′  R: 5′-ACAGCCGTGGTTTGGAAGAA-3′ |
| *Pdha1* | Mouse | F: 5′-ACTTCTATGGAGGCAACGGC-3′  R: 5′-ATTGTACTTGCAGGCCAGGG-3′ |
| *Dlat* | Mouse | F: 5′-CTCCGCATCAGAAGGTTCCA-3′  R: 5′-CCAGGCTCTCAAACCCAACA-3′ |
| *Aco2* | Mouse | F: 5′-GCAAAGCCAACTCTGTACGC-3′  R: 5′-CTCCAATCACCACCCACCTG-3′ |
| *Idh3b* | Mouse | F: 5′-GTCACTCGCACCAAGTCTCA-3′  R: 5′-TGTTGGCTTTATGGACGGCT-3′ |
| *Sdhd* | Mouse | F: 5′-TGGTCAGACCCGCTTATGTG-3′  R: 5′-GGTCCAGTGGAGAGATGCAG-3′ |
| *Ppargc1a* | Mouse | F: 5′-CACCTTCCTCTCCTCCTTCTTTTAC-3′  R: 5′-AGAGCAGCACACTGGTTGGAA-3′ |
| *Ndufb7* | Mouse | F: 5′-CTACTGCGAGCACCTGGATT-3′  R: 5′-TACCCGTGCTTCCTTCAGTG-3′ |
| *Coq9* | Mouse | F: 5′-AGGGATGCAGTGGAAACCAG-3′  R: 5′-CGGCGGGTGTACCAGTTAAA-3′ |
| *Uqcrq* | Mouse | F: 5′-ATCTCCTACAGCTTGTCGCC-3′  R: 5′-CTGCTCAAACTCCTGGTTGC-3′ |
| *MT-Co2* | Mouse | F: 5′-ACCGAGTCGTTCTGCCAATA-3′  R: 5′-TAAGTCCTAGGGAGGGGACTG-3′ |
| *Cox6c* | Mouse | F: 5′-CAAGCGTCTGCGGGTTCATA-3′  R: 5′-CACGCCAAACTTATAGGCAGC-3′ |
| *Atp5me* | Mouse | F: 5′-GGTTCAGGTCTCTCCACTCA-3′  R: 5′-CTCCGCTGCTATTCTCCTCTC-3′ |
| *Tnni3* | Mouse | F: 5′-GGCTGATGAGAGCAGCGAT-3′  R: 5′-GGCATAGGCTCGGTAGTTGG-3′ |
| *Myh7* | Mouse | F: 5′- CAGCAGTTGGATGAGCGACT -3′  R: 5′- GCTCATCCTCAATCCTGGCAT -3′ |
| *Hmgcl* | Mouse | F: 5′-TGTACCCACCCCAGTGAAGA -3′  R: 5′- GAGTGGTCAGCCATCTGTGG-3′ |
| *Hmgcs2* | Mouse | F: 5′-GCCTACCGCAAGAAGATCCA -3′  R: 5′- TGAACATCAACCGAGCCAGG-3′ |
| *Ppara* | Mouse | F: 5′-CGTCACGGAGCTCACAGAAT-3′  R: 5′-ACTCGCGTGTGATAAAGCCA-3′ |
| *Cpt1a* | Mouse | F: 5′-GTGGTGTCCAAGTATCTGGC-3′  R: 5′-AGGAAACACCATAGCCGTCA-3′ |
| *Atgl* | Mouse | F: 5′-GAGCTTCGCGTCACCAACA-3′  R: 5′-GCCAGCAAAGGGTTGGGTTG-3′ |
| *Hsl* | Mouse | F: 5′-CTCAGAGCCTTCAGACAGCC-3′  R: 5′-CCGTCCATGGGTCTCACTTC-3′ |
| *Mgll* | Mouse | F: 5′-TTTCACCTCTGGTCCTTGCC-3′  R: 5′-GCAGAACCCTCCGACTTGTT-3′ |
| *Plaat3* | Mouse | F: 5′-TAGCACCCATACCAGAACCC-3′  R: 5′-GCGATTTCACTTGGAGGAGC-3′ |
| *Abhd5* | Mouse | F: 5′-ATGCTTCAGCGGATAGGTGG-3′  R: 5′-ATACACATAATGCCCCGCCC-3′ |
| *Cidec* | Mouse | F: 5′-GGAAGCCCCCATCAGAACAG-3′  R: 5′-GAGGGTTGCCTTCACGTTCA-3′ |
| *Plin1* | Mouse | F: 5′-ATGCCCTGAAGGGTGTTACG-3′  R: 5′-TGTCTCGGAATTCGCTCTCG-3′ |
| *Fabp4* | Mouse | F: 5′-TTCCTTCAAACTGGGCGTGG-3′  R: 5′-TTGTGGTCGACTTTCCATCCC-3′ |
| *Cidea* | Mouse | F: 5′-GGACAGAAATGGACACCGGG-3′  R: 5′-TGACATTGAGACAGCCGAGG-3′ |
| *Idh2* | Mouse | F: 5′-CACCCGCCATTACCGAGAAC-3′  R: 5′-GGCAAAGATGCTGGCAATAGG-3′ |
| *Fh* | Mouse | F: 5′-GGAGGTGTGACAGAACGCAT-3′  R: 5′-CATCTGCTGCCTTCATTATTGC-3′ |
| *Mdh2* | Mouse | F: 5′-CCCCTTTCACTCCTGCTGAA-3′  R: 5′-GGGATGACCACCACATCACA-3′ |
| *Mt-atp6* | Mouse | F: 5′-GCAGTCCGGCTTACAGCTAA-3′  R: 5′-GGTAGCTGTTGGTGGGCTAA-3′ |
| *Gatm* | Mouse | F: 5′-CCTCCAACACCAGTCATCCC-3′  R: 5′-TCTACCATCACGCGCTTCTC-3′ |
| *Gamt* | Mouse | F: 5′-ACTTTATTAAGAATCATGCCTTCCG-3′  R: 5′-CACCTGCGTCTCCTCAAACA-3′ |
| *Cdo1* | Mouse | F: 5′-GAGAACGTCAGCCACACAGA-3′  R: 5′-CAAAGGCGTGGCATGTATCG-3′ |
| *Csad* | Mouse | F: 5′-TGTCACTACTCCATCACCAAGGG-3′  R: 5′-GCCTTGACCACTCGGACACT-3′ |
| *Slc6a6* | Mouse | F: 5′-GTTTTGTGTCTGGCTTCGCA-3′  R: 5′-CATCAGCAATGTCCACCCCT-3′ |
| *Slc19a2* | Mouse | F: 5′-GAACTACGCGCAGGGATTGT-3′  R: 5′-AAACACAGCACTAGCACCCA-3′ |
| *Nppa* | Mouse | F: 5′-TCCTCGTCTTGGCCTTTTGG-3′  R: 5′-CCAGGTGGTCTAGCAGGTTC-3′ |
| *Nppb* | Mouse | F: 5′-AAAGTCGGAGGAAATGGCCC-3′  R: 5′-ATCCGGTCTATCTTGTGCCC-3′ |
| *Hacd1* | Mouse | F: 5′-GAGGCCAGCGACGAGAAG-3′  R: 5′-GCCGTCATGGCGATATTGTA-3′ |
| *Hacd2* | Mouse | F: 5′-ATACGCAGCTCTGCCCTTTG-3′  R: 5′-GGGGGAACAGTGGAATGTAGG-3′ |
| *CD36* | Mouse | F: 5′-GATCGGAACTGTGGGCTCAT-3′  R: 5′-ACTGGCATGAGAATGCCTCC-3′ |
| *Lpl* | Mouse | F: 5′-AAACCCCAGCAAGGCATACA-3′  R: 5′-GTAGGGCATCTGAGAGCGAG-3′ |
| *Hagh* | Mouse | F: 5′-AACACCCTGCCATACTTCGG-3′  R: 5′-AAGCAGCGCCTTGTACATCT-3′ |
| *Lvd* | Mouse | F: 5′-CCAGGAGAGTAAGGGGGTCT-3′  R: 5′-ACAGTCCTGGAATTGGCCG-3′ |
| *Acadsb* | Mouse | F: 5′-CAAGCAAGTGCATCGAGTGG-3′  R: 5′-CCGATCTTGGCATCTCGGAA-3′ |
| *Acad8* | Mouse | F: 5′-TCGATCCTTCCTTGGGGCTA-3′  R: 5′-TTTCCCGAGCTGCAAAGTCA-3′ |
| *Dld* | Mouse | F: 5′-GCTGCAAACAGCAGAGCTAA-3′  R: 5′-CGCCTCGTTCACCATTTCTC-3′ |
| *Fabp1* | Mouse | F: 5′-GTCCGCAATGAGTTCACCCT-3′  R: 5′-GCTTGACGACTGCCTTGACT-3′ |
| *Slc22a5* | Mouse | F: 5′-GCTCTCTGGTGGTTCATCCC-3′  R: 5′-GGTGCAACTGAGGCTTCGTA-3′ |
| *Cel* | Mouse | F: 5′-ACTACCTGGCCTTCATCCCT-3′  R: 5′-GGTAGCAAATAGGTGGCCGT-3′ |
| *Pnlip* | Mouse | F: 5′-ACCGATGCTCAGTTTGTGGACG-3′  R: 5′-CCTGGCATTTCGATTCCTCCGT-3′ |
| *Ndufs1* | Mouse | F: 5′-TCCAAAGGTTGTCGCTGCTT-3′  R: 5′-CATTCACCTCCCTGGTCACAA-3′ |
| *Acsl1* | Mouse | F: 5′-GCAACCCCAAAGGAGCAATG-3′  R: 5′-GCACATCATCTGTGGAAGCG-3′ |
| *18S* | Mouse | F: 5′-GCAATTATTCCCCATGAACG-3′  R: 5′-GGCCTCACTAAACCATCCAA-3′ |
| *Rplp0* | Mouse | F: 5′-AGATTCGGGATATGCTGTTGGC-3′  R: 5′-TCGGGTCCTAGACCAGTGTTC-3′ |
| *Pfkfb3* | Mouse | F: 5′-CGCAATAGTGTCACCCCACT-3′  R: 5′-CTTCTGGGAAGAGTCGGCAC-3′ |

**Key resource table**

| REAGENT or RESOURCE | SOURCE | IDENTIFIER |
| --- | --- | --- |
| 1. Antibodies | | |
| Rabbit anti-Phospho-AMPK alpha (Thr172) | Cell Signaling Technology | 2535,  RRID: AB_331250 |
| Mouse anti- AMPK alpha 1 | Santa Cruz Biotechnology | sc-130394,  RRID: AB_2169710 |
| Mouse anti-Phospho-mTOR (Ser2448) | Proteintech Group | 67778-1-Ig,  RRID: AB_2889842 |
| Mouse anti- mTOR | Proteintech Group | 66888-1-Ig,  RRID: AB_2882219 |
| Mouse anti-Phospho-LKB1 (Ser431) | Santa Cruz Biotechnology | sc-271924,  RRID: AB_10610759 |
| Mouse anti-LKB1 (Ley 37D/G6) | Santa Cruz Biotechnology | sc-32245,  RRID: AB_627890 |
| Rabbit anti- PINK1 | Proteintech Group | 23274-1-AP,  RRID: AB_2879244 |
| Mouse anti- ATP5A1 | Proteintech Group | 66037-1-Ig,  RRID: AB_11044196 |
| Rabbit anti- OXCT1 | Proteintech Group | 12175-1-AP，  RRID: AB_2157444 |
| Rabbit anti- ATGL | Proteintech Group | 55190-1-AP，  RRID: AB_11182818 |
| Rabbit anti-ACAT1 | Proteintech Group | 16215-1-AP, RRID:AB_2220210 |
| Rabbit anti- SUCLG1 | Proteintech Group | 14923-1-AP,  RRID: AB_2197177 |
| Rabbit anti- Phospho-ERK1/2 | Proteintech Group | 28733-1-AP,  RRID: AB_2881202 |
| Rabbit anti-ERK1/2 | Proteintech Group | 11257-1-AP,  RRID: AB_2139822 |
| Rabbit anti- ACADL | Proteintech Group | 17526-1-AP,  RRID: AB_2219661 |
| Mouse anti-Alpha Tubulin | Proteintech Group | 66031-1-Ig, RRID:AB_11042766 |
| Goat anti-mouse [IgG](https://www.abcam.cn/products/secondary-antibodies/goat-mouse-igg-hl-alexa-fluor-647-ab150115.html), HRP-conjugated | Proteintech Group | SA00001-1  RRID: AB_2722565 |
| Goat anti-rabbit [IgG](https://www.abcam.cn/products/secondary-antibodies/goat-mouse-igg-hl-alexa-fluor-647-ab150115.html), HRP-conjugated | Proteintech Group | SA00001-2,  RRID: AB_2722564 |
| Goat anti-mouse [IgG](https://www.abcam.cn/products/secondary-antibodies/goat-mouse-igg-hl-alexa-fluor-647-ab150115.html), CoraLite488-conjugated | Proteintech Group | SA00013-1,  RRID: AB_2810983 |
| Goat anti-rabbit [IgG](https://www.abcam.cn/products/secondary-antibodies/goat-mouse-igg-hl-alexa-fluor-647-ab150115.html), CoraLite647-conjugated | Proteintech Group | SA00014-9,  RRID: AB_2935614 |
| 2. Bacterial and virus strains | | |
| pAAV9-TBG-mCherry-mir30shRNA(*mHmgcs2*)-WPRE | PackGene Biotech | N/A |
| pAAV9-cTnT-EGFP-shRNA(*mOxct1*)-WPRE | VectorBuilder | N/A |
| pAAV9-TBG-*hFGF21*-HA-IRES-EGFP-WPRE | VectorBuilder | N/A |
| pAAV9-TBG-shRNA-*Scramble* | VectorBuilder | N/A |
| pAAV9-cTnT-shRNA-*Scramble* | VectorBuilder | N/A |
| 3. Chemicals, peptides, and recombinant proteins | | |
| Recombinant human FGF21 | This paper | N/A |
| Picro-Sirius red | Leagene Biotechnology | DC0041 |
| Polyethylenimine (PEI) | Polysciences | 23966 |
| PrimeSTAR^®^ HS DNA Polymerase | Takara | R010A |
| DAPI | Solarbio | C0065 |
| TRIzol | Invitrogen | 15596026CN |
| Hieff Trans^®^ Liposomal Transfection Reagent | YEASEN | 40802ES03 |
| 1,3-butanediol | MedChemExpress | HY-77490A |
| Sodium palmitate | Sigma-Aldrich | P9767 |
| Atglistatin | MedChemExpress | HY-15859 |
| AICAR | MedChemExpress | HY-13417 |
| L-Leucine | MedChemExpress | HY-N0486 |
| Torin 1 | MedChemExpress | HY-13003 |
| Devimistat | MedChemExpress | HY-15453 |
| iFluor 488-Wheat Germ Agglutinin (WGA) Conjugate | Solarbio | I3300 |
| Glucose monohydrate | Sigma-Aldrich | Y0001745 |
| 4. Critical Commercial Assays | | |
| Modified Masson's Trichrome Stain kit | Solarbio | G1346 |
| EndoFree Maxi Plasmid kit | TIANGEN | DP117 |
| HiScript III 1st Strand cDNA Synthesis Kit | Vazyme | R312 |
| ChamQ Universal SYBR qPCR Master Mix | Vazyme | Q711 |
| CheKine™ Micro Mitochondrial complex I Activity Assay Kit | Abbkine | KTB1850 |
| CheKine™ Micro Mitochondrial complexⅡ Activity Assay Kit | Abbkine | KTB1860 |
| CheKine™ Micro Mitochondrial complex Ⅲ Activity Assay Kit | Abbkine | KTB1870 |
| CheKine™ Micro Mitochondrial complex Ⅳ Activity Assay Kit | Abbkine | KTB1880 |
| Enhanced BCA Protein Assay Kit | Beyotime | P0010 |
| Triglyceride assay kit | Jiancheng Bioengineering | A110-1-1 |
| Nonesterified Free fatty acids assay kit | Jiancheng Bioengineering | A042-2-1 |
| β-Hydroxybutyric acid (β-HB) Content Assay Kit | Solarbio | BC5085 |
| Mouse phosphocreatine ELISA KIT | Biotopped | TOPEL30254 |
| Phospholipid Assay Kit | CELL BIOLABS, INC | MET-5085 |
| Liver/Muscle glycogen assay kit | Jiancheng Bioengineering | A043-1-1 |
| Triglyceride assay kit | Jiancheng Bioengineering | A110-1-1 |
| Mouse Fibroblast Growth Factor 21 (FGF21) ELISA Kit | JONLNBIO | JL22924 |
| Human Fibroblast Growth Factor 21 (FGF-21) ELISA Kit | JONLNBIO | JL19322 |
| 5. Experimental models: Organisms/strains | | |
| Mouse: C57BL/6J | Beijing Vital River Laboratory Animal Technology | Strain code: 219 |
| Mouse: *Fgf21^-/-^* | Original gift from Dr. Steven Kliewer | N/A |
| Mouse: *Fgfr1^flox/flox^* | Gift from Dr. Zhihfeng Huang | S-CKO-02414 |
| Mouse: *Stk11^flox/flox^* | Cyagen Biosciences (Suzhou) | S-CKO-05368 |
| Mouse: *Myh6^C^*^re^ | Cyagen Biosciences (Suzhou) | C001009 |
| Mouse: A*dipoQ^Cre^* | Cyagen Biosciences (Suzhou) | C001186 |
| 6. Recombinant DNA | | |
| Oligonucleotides | | |
| shRNA targeting sequence for mouse *Hmgcs2:* AGCCATGTCTGTCTACACGAAA | This paper | N/A |
| shRNA targeting sequence for mouse *Oxct1*: ACGGAAGGATGTCAGTAATCAA | This paper | N/A |
| Primers sequences for mouse genotyping | Table S4 | N/A |
| Primers sequences for qRT-PCR | Table S5 | N/A |
| 7. Deposited data | | |
| The raw data of cardiac RNA-Seq generated | This paper | PRJNA1189445 |
| The raw of serum metabolomics generated | This paper | Table S1, MTBLS11726 |
| The raw of cardiac energy metabolomics generated | This paper | Table S2, MTBLS11725 |
| 8. Software and algorithms | | |
| GraphPad Prism 9 | GraphPad | https://www.graphpad.com/scientific-software/prism/ |
| ImageJ | NIH | https://imagej.net/Fiji |
| R package (v4.2.2) | The R Foundation | https://www.r-project.org |
| 9. Other | | |
| Standard rodent chow | Dyets | D100000 |
| Rodent Diet With 60 kcal% Fat | Research diets | D12492 |
