# Supplementary material for: A two-strata energy flux system driven by a stress hormone prioritizes cardiac energetics
Source: Signal Transduct Target Ther. 2025 Sep 26;10:315. doi: 10.1038/s41392-025-02402-9 (PMC12464335; doi:10.1038/s41392-025-02402-9)
Supplement: Supplementary file 2 — Data S1 [file 41392_2025_2402_MOESM2_ESM.docx]

Data S1. Uncropped western blots for

A two-strata energy flux system driven by a stress hormone prioritizes cardiac energetics

Zhiheng Rao^1,2,☨^, Zhichao Chen^1,☨^, Yuxuan Bao^1,☨^, Zhenzhen Lu^1,☨^, Yuli Tang^1^, Jiamei Zhu^1^, Jianjia Ma^1^, Siyang Dong^1^, Jiawei Shi^1^, Suhui Sheng^1^, Yajing Chen^1^, Jiaojiao Wang^1^, Alan Vengai Mukondiwa^1^, Ziyue Li^1^, Xulan Wang^1^, Zibo Huang^1^, Chi Li^1^, Wumengwei Ding^1^, Mengjie Chen^1^, Ziyi Han^1^, Cong Wang^1^, Xuebo Pang^1^, Xiaojie Wang^1,3^, Hong Zhu^4^, Li Lin^1,3^, Zhifeng Huang^1,3^, Weiqin Lu^5^, Xiaokun Li^1,3,^*, Yongde Luo^1,3,4,6,^*

*Correspondence to: yongdeluo08@wmu.edu.cn (Y.L.), xiaokunli@wmu.edu.cn (X.L.)

**This file includes:**

Data S1. Uncropped western blots

**Data S1. Uncropped western blots**

**Figure 7:**

**Figure S16:**

**Figure S25:**

**Figure S27:**
